# Simultaneous Fe^2+^/Fe^3+^ imaging shows Fe^3+^ over Fe^2+^ enrichment in Alzheimer’s disease mouse brain

**DOI:** 10.1126/sciadv.ade7622

**Published:** 2023-04-19

**Authors:** Yuting Wu, Seyed-Fakhreddin Torabi, Ryan J. Lake, Shanni Hong, Zhengxin Yu, Peiwen Wu, Zhenglin Yang, Kevin Nelson, Weijie Guo, Gregory T. Pawel, Jacqueline Van Stappen, Xiangli Shao, Liviu M. Mirica, Yi Lu

**Affiliations:** ^1^ Department of Chemistry, University of Illinois at Urbana-Champaign, Urbana, IL 61801, USA.; ^2^ Department of Chemistry, University of Texas at Austin, Austin, TX 78712, USA.; ^3^ Department of Biochemistry, University of Illinois at Urbana-Champaign, Urbana, IL 61801, USA.; ^4^ Department of Molecular Bioscience, University of Texas at Austin, Austin, TX 78712, USA.

## Abstract

Visualizing redox-active metal ions, such as Fe^2+^ and Fe^3+^ ions, are essential for understanding their roles in biological processes and human diseases. Despite the development of imaging probes and techniques, imaging both Fe^2+^ and Fe^3+^ simultaneously in living cells with high selectivity and sensitivity has not been reported. Here, we selected and developed DNAzyme-based fluorescent turn-on sensors that are selective for either Fe^2+^ or Fe^3+^, revealing a decreased Fe^3+^/Fe^2+^ ratio during ferroptosis and an increased Fe^3+^/Fe^2+^ ratio in Alzheimer’s disease mouse brain. The elevated Fe^3+^/Fe^2+^ ratio was mainly observed in amyloid plaque regions, suggesting a correlation between amyloid plaques and the accumulation of Fe^3+^ and/or conversion of Fe^2+^ to Fe^3+^. Our sensors can provide deep insights into the biological roles of labile iron redox cycling.

## Teaser

Selective Fe^2+^/Fe^3+^ DNAzyme sensors are created to reveal an increased Fe^3+^/Fe^2+^ ratio in Alzheimer’s disease mouse brain.

## INTRODUCTION

Redox-active metal ions play key roles in many biological processes such as oxygen transport, energy production, and oxidative stress–related neurodegenerative diseases (*1*, *2*). A primary example is iron (Fe), which is mostly present in ferrous [Fe(II)/Fe^2+^] or ferric [Fe(III)/Fe^3+^] states in living organisms (*3*, *4*). Dyshomeostasis of iron and its abnormal redox cycling can lead to ferroptosis, an iron-dependent programmed cell death pathway, which is a key process in many neurodegenerative diseases including Alzheimer’s disease (AD) (*5*–*9*). In addition, ferroptosis has emerged as a promising therapeutic approach to cancers (*10*, *11*). However, how redox equilibrium and dynamic speciation are involved in ferroptosis and related to AD or cancer remains poorly understood, partly because of a lack of highly selective sensors that allow for simultaneous monitoring of both Fe^2+^ and Fe^3+^. Such sensors require detection of either Fe^2+^ or Fe^3+^ without cross-reactivity with the opposite Fe oxidation state. Because of the chemical and physical similarities between these oxidation states, it has been quite challenging to develop sensors to detect both Fe^2+^ and Fe^3+^ simultaneously with high selectivity and sensitivity.

To achieve sensing of different redox states of iron, various methods have been explored but with limited success either in living cells or in vivo. For example, laboratory techniques, such as inductively coupled plasma mass spectrometry (*12*, *13*), electron paramagnetic resonance (*14*), x-ray fluorescence (*15*), and magnetic resonance imaging (MRI) (*16*–*18*) have been developed but cannot readily provide spatial or temporal information in vivo because of their restrictive requirements for sample pretreatment or excessive time needed for data collection. It has been shown that “labile” iron pools, which comprise only a small portion of total iron, play critical roles in many cellular processes, including lipid oxidation during ferroptosis and generating free radicals in AD (*19*–*21*), and all of the above methods can measure only the total iron without differentiating labile iron pools. To visualize these labile iron pools, histochemical methods based on potassium ferricyanide or potassium ferrocyanide was developed to distinguish Fe^2+^ from Fe^3+^ and acquire spatial information (*22*), but this method can only detect Fe^2+^ or Fe^3+^ separately on fixed tissue slices, not in living cells or in vivo, because the detection is based on forming insoluble blue pigments. In addition, ferricyanide can react with many other metal ions, such as Zn^2+^ and Cu^2+^, and thus is vulnerable to interference from these metal ions.

Fluorescence sensors have also been developed to visualize labile Fe^2+^ and Fe^3+^ simultaneously in vivo and provide spatiotemporal information in living cells. However, most of these methods either have low selectivity for Fe^2+^ and Fe^3+^ over other metal ions, require organic solvents, or cannot be adapted readily for in vivo sensing applications. Recently, some Fe^2+^ sensors based on organic molecules and fluorophores have achieved sufficient selectivity and sensitivity for imaging in cells (*23*–*26*) and in mouse models (*27*, *28*). To image two different oxidation states of the same metal ions, such as Fe^2+^ and Fe^3+^ simultaneously, we need two sensors that are not only specific for the respective Fe^2+^ or Fe^3+^ but also two fluorophores that do not have much overlapping excitation and emission spectra to avoid interference in the detection. Because the target recognition and fluorescent readouts of the organic molecule sensors are coupled together, it is difficult to replace the fluorophore with one that has a different fluorescence emission spectrum to avoid overlap of fluorescent signals. Changing fluorophore moieties for these sensors normally requires redesign of the sensors, which can adversely affect their other properties, such as loss of brightness of fluorescence, reduced selectivity, or change of subcellular localization of the sensor (*29*). Therefore, to the best of our knowledge, the simultaneous monitoring of two oxidation states of the same metal ion in living cells or in vivo has not yet been reported. As a result, the redox equilibrium and dynamic distribution of Fe^2+^ and Fe^3+^ have not been investigated, although they have been hypothesized to play important roles on many biological processes including ferroptosis in neurodegenerative diseases such as AD.

**Fig. 1.**
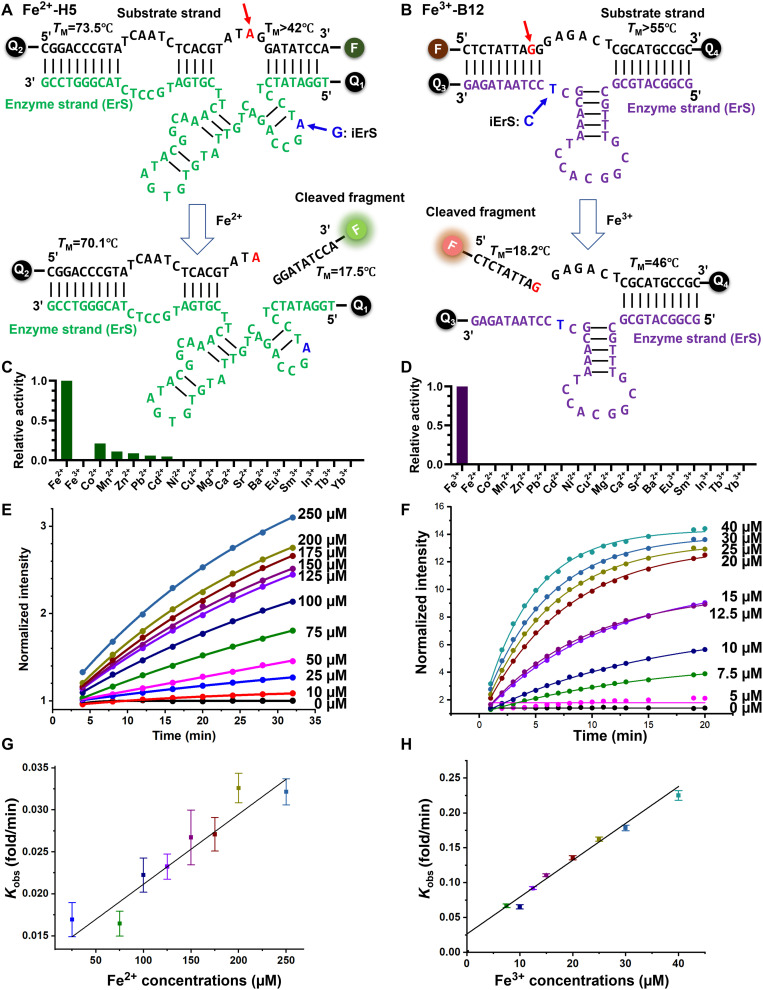
The sequences of the Fe^2+^- and Fe^3+^-specific DNAzymes and the design, sensitivity, and selectivity of their fluorescent sensors. (**A**) Secondary structure of the trans-cleaving Fe^2+^-specific DNAzyme [Fe(II)-H5], consisting of an enzyme strand shown in green with an Iowa Black FQ quencher (Q1) at the 5′ end, and a substrate strand shown in black with the same quencher (Q2) at the 5′ end and an Alexa Fluor 488 fluorophore (F1) at the 3′ end. (**B**) Secondary structure of the trans-cleaving Fe^3+^-specific DNAzyme [Fe(III)-B12], consisting of an enzyme strand shown in purple with an Iowa Black RQ quencher (Q3) at the 3′ end, and a substrate strand shown in black with a 5′ Alexa Fluor 647 fluorophore (F2) and a second Iowa Black RQ quencher (Q4) at the 3′ end. Both DNAzymes contain a ribonucleotide cleavage site (red, arrow). In addition, a mutation in the enzyme strand shown in blue [A to G in the Fe(II)-H5 DNAzyme and T to C in the Fe(III)-B12 DNAzyme] rendered the respective DNAzymes inactive and thus served as negative controls. (**C** and **D**) Selectivity of the trans-cleaving Fe(II)-H5 (C) and Fe(III)-B12 (D) DNAzymes. Fraction of the cleaved substrate in the presence of different metal ions in 20 mM acetate buffer (pH 6.0), 5 mM bis-tris, and 200 mM NaCl analyzed via denaturing polyacrylamide gel electrophoresis (PAGE). (**E** and **F**) Normalized fluorescence intensity of the Fe(II)-H5 DNAzyme (E) and the Fe(III)-B12 DNAzyme (F) sensors in response to different concentrations of Fe^2+^ or Fe^3+^, respectively. (**G** and **H**) The initial fluorescence turn-on rates (*k*_obs_) of the sensors at different iron concentrations are shown in (G) for the Fe(II)-H5 DNAzyme and in (H) for the Fe(III)-B12 DNAzyme.

To overcome the technical barrier for simultaneous monitoring Fe^2+^ and Fe^3+^ in vivo and to fill a major knowledge gap of redox equilibrium, dynamic distribution of Fe^2+^/Fe^3+^, and their roles in neurodegenerative diseases, we take advantage of DNAzyme-based “catalytic beacon” sensors. DNAzymes, also called deoxyribozymes, are DNA molecules that display enzymatic activities, such as protein enzymes and ribozymes, in the presence of a cofactor such as metal ions (*30*–*36*). DNAzymes are typically isolated from a large DNA library of up to 10^15^ different sequences through a combinatorial process called in vitro selection (*37*). Among them, RNA-cleaving DNAzymes are of particular interest for sensing metal ions because these DNAzymes are often specific for a certain metal ion cofactor (*30*, *32*). By conjugating a fluorophore at the end of the enzyme strand, two quenchers at the opposite termini of the enzyme strand and complementary substrate strand, respectively, we take the advantage of melting temperature differences before and after DNAzyme-catalyzed cleavage of the substrate strand and have developed a catalytic beacon approach (*38*–*48*) that produces metal ion–specific fluorescent turn-on sensors (Fig. 1, A and B). Because the fluorophore is physically separated from the metal-binding site and fluorescent signal arises from the release of the fluorophore-labeled substrate upon cleavage, this approach can be used to sense metal ions using any fluorophore. Therefore, it is possible to use DNAzyme beacons labeled with different fluorophores that have distinct emission wavelengths for simultaneous monitoring of two or more targets. Through efforts from many laboratories, including our laboratory, DNAzymes highly selective for different metal ions have been obtained, including Cr^3+^ (*49*), Ca^2+^ (*50*), Cd^2+^ (*51*), Co^2+^ (*52*), Cu^2+^ (*53*), Mg^2+^ (*54*), Pb^2+^ (*30*), UO_2_^2+^ (*40*), Zn^2+^ (*32*, *55*, *56*), Ag^+^ (*57*), Li^+^ (*58*), and Na^+^ (*59*, *60*). Despite decades of success using this approach, no in cellulo or in vivo DNAzyme sensor that can differentiate different oxidization states of the same metal ion has been reported.

In this study, we report in vitro selection and development of DNAzyme sensors with high specificity for either Fe^2+^ or Fe^3+^, which allows us to visualize both Fe^2+^ and Fe^3+^ simultaneously in living cells and brain slices of AD mice models. Correlated signal changes were observed with the regulation of iron levels by addition of transferrin (Tf), an iron transport protein (*61*), or deferoxamine (DFO), an iron chelator (*62*). We further apply these sensors to detect iron changes during ferroptosis in living cells and observed a decrease of Fe^3+^/Fe^2+^ redox ratio over time, which suggests that iron is a potential source related to the oxidative stress accumulation in this cell death pathway (*7*). The sensors provided spatial distributions of Fe^2+^ and Fe^3+^, as well as iron redox ratios, revealing a statistically significant increase in the Fe^3+^/Fe^2+^ ratio surrounding amyloid plaque regions but not in other brain regions and suggesting that not only total iron but also iron redox cycling play a key role in the progression of AD. These finding also suggests a correlation between amyloid plaques and the accumulation of Fe^3+^ and/or the conversion from Fe^2+^ into Fe^3+^, which provides potential direction for further functional study to understand metal redox in AD progression. These results demonstrate that simultaneous monitoring of Fe^2+^ and Fe^3+^ using iron-specific DNAzyme-based sensors provides deeper insight into the roles of redox cycling of labile iron in neurodegenerative diseases.

## RESULTS

### In vitro selection of Fe^2+^ and Fe^3+^-specific DNAzymes

To obtain Fe^2+^-specific DNAzymes, in vitro selection was conducted in an anaerobic glove box because Fe^2+^ is readily oxidized to Fe^3+^ in the presence of oxygen (fig. S1). Parallel selections were carried out in the presence or absence of 1 mM glutathione, a highly abundant cytosolic metabolite predicted to form a complex with Fe^2+^ intracellularly (*63*). A negative selection against the selection buffer without Fe^2+^ was incorporated before the first round, and counter selections against Mn^2+^, Co^2+^, Zn^2+^, Cd^2+^, and Pb^2+^ were introduced starting from the third round of the selections to remove DNAzymes that catalyzed the cleavage of the substrate in the presence of other components in the selection buffer or other divalent metal ions. These additional steps were necessary to improve the selectivity of the isolated DNAzymes (fig. S2) (*59*). The selection was monitored by measuring the percent cleavage activity in the presence of Fe^2+^ in each round and was continued for nine rounds (fig. S3). Enrichment of highly Fe^2+^-specific DNAzymes and effectiveness of counter selection was confirmed by evaluating cleavage activity of the selected pools in the presence of Fe^2+^ or Fe^3+^ or several other divalent metal ions (figs. S4 and S5). From cloning and sequencing, a total of 149 sequences were identified from different conditions (figs. S6 to S9 and table S1). Upon testing representative individual sequences for their Fe^2+^-dependent activity, the most active and selective sequence (fig. S10), named Fe(II)-H5, was chosen for sensor development. First, on the basis of the predicted secondary structures of the Fe(II)-H5 cis-acting DNAzyme, we generated six trans-acting DNAzymes consisting of a separate enzyme (E) and substrate (S) strands by truncating sequences at different sites and testing their cleavage activity (fig. S11). The truncation studies resulted in an active trans-cleaving DNAzyme with minimal catalytic sequence (truncation-2b in fig. S11). Such a trans-cleaving DNAzyme is suitable for conjugating to fluorophore and quenchers and compatible with the catalytic beacon sensing strategy (Fig. 1A).

With the goal of selecting Fe^3+^-specific DNAzymes, several in vitro selection conditions and strategies were tested. We found that the choice of in vitro selection condition including components to solubilize Fe^3+^ while keeping it accessible to the DNA molecules in the initial library is critical to the successful isolation of Fe^3+^-specific DNAzymes. As a result of these tests, in vitro selection was conducted in the presence of 5 mM bis-tris in an acetate buffer at pH 5.5, which we found was able to stabilize Fe^3+^ in a soluble form (fig. S12) because Fe^3+^ is known to readily form insoluble iron hydroxide complexes at pH > 2.6 if not stabilized with a weakly chelating agent such as bis-tris (fig. S13). Inclusion of strong chelators such as citrate ions that can solubilize Fe^3+^ resulted in failure in isolating Fe^3+^-specific DNAzymes, perhaps because of the inability of DNA molecules to compete with such chelators in binding to Fe^3+^. Negative selections against the same buffer without Fe^3+^ were incorporated starting from round 1 before each positive selection to decrease the chance of isolating Fe^3+^-independent DNAzymes. The in vitro selection process was continued until the ratio of Fe^3+^-specific activity over background cleavage started to drop after round 9 (fig. S14). The enriched pools did not show substantial cleavage activity in the presence of several other metal ions at 50 μM, such as Fe^2+^, Mg^2+^, Ca^2+^, Sr^2+^, Ba^2+^, Mn^2+^, Co^2+^, Ni^2+^, Cu^2+^, Zn^2+^, Cd^2+^, Pb^2+^, Eu^3+^, Sm^3+^, In^3+^, Tb^3+^, and Yb^3+^ (fig. S15). From cloning and sequencing, a total of 157 DNAzymes were isolated from different selection conditions (table S2). The resulting sequences were aligned on the basis of their primary sequence similarities (figs. S16 to S19). The activity of several representative DNAzymes were tested, and one of the most active DNAzymes, called Fe(III)-B12 (fig. S20), was converted into trans-cleaving DNAzymes, by truncation studies. The truncation study was based on predicted secondary structures of the cis-acting Fe(III)-B12 DNAzyme (fig. S21). The cleavage activities of three trans-acting DNAzymes generated by truncating B12 sequence at different sites were tested. The secondary structure of trans-acting Fe(III)-B12 DNAzyme with minimal catalytic region (fig. S21) was used to design the Fe^3+^-dependent DNAzyme-based fluorescent sensor (Fig. 1B). To confirm the selectivity of the trans-cleaving iron DNAzymes, we evaluated the cleavage activity of the Fe(II)-H5 and Fe(III)-B12 DNAzymes in the presence of different metal ions at 100 μM including Fe^2+^, Fe^3+^, Co^2+^, Mn^2+^, Zn^2+^, Pb^2+^, Cd^2+^, Ni^2+^, Cu^2+^, Mg^2+^, Ca^2+^, Sr^2+^, Ba^2+^, Eu^3+^, Sm^3+^, In^3+^, Tb^3+^, and Yb^3+^. Both DNAzymes exhibit excellent selectivity for their respective iron oxidation state (Fig. 1, C and D).

### Conversion and characterization of fluorescent DNAzyme sensors

To convert the Fe^2+^- and Fe^3+^-specific DNAzymes into fluorescent sensors, we applied the catalytic beacon design (Fig. 1, A and B) by incorporating a fluorophore (F) on one end of the substrate strand and an intermolecular quencher (Q1 or Q3) on opposite termini of the enzyme strand. The enzyme strand can bind to the substrate strand through DNA-DNA hybridization of the two binding arms. In addition, the substrate strand, which contains a ribonucleotide cleavage site, is labeled with an intramolecular quencher (Q2 or Q4) to suppress background fluorescence without substrate strand being cleaved (*39*). The binding arms are designed to be long enough that the melting temperature of the entire complex is higher than the desired ambient temperature (e.g., 37°C for cellular studies). Therefore, the enzyme and uncleaved substrate strands can stably hybridize to each other under assay conditions. Upon metal-induced cleavage of the substrate at the internal ribonucleotide site, the resulting fluorophore-labeled fragment has a much lower melting temperature (<20°C). This decrease in melting temperature allows the fluorophore-labeled fragment to dissociate from the complex, resulting in the release of the fluorophore from both inter- and intramolecular quenchers, followed by a substantial increase in fluorescent signal. Because this design decouples the fluorophores from metal-dependent DNAzyme-based cleavage of the substrate, any fluorophore and quencher pairs can be readily adapted to the sensors to achieve sensing with different excitation and emission wavelengths. Moreover, both Fe^2+^ and Fe^3+^ are known to quench fluorescence of fluorophores nonspecifically. Separating the Fe^2+^- and Fe^3+^-binding site away from the fluorophore helps minimize this issue, allowing to build Fe^2+^- and Fe^3+^-specific DNAzyme sensors with turn-on signals.

Taking advantage of the above design, we incorporated Alexa Fluor 488 in the Fe^2+^-selective DNAzyme [Fe(II)-H5] and Alexa Fluor 647 in the Fe^3+^-selective DNAzyme [Fe(III)-B12] for simultaneous detection of both oxidation states of iron using two different fluorescent channels. Furthermore, to eliminate the artifact of fluorescent signals due to anything other than Fe^2+^- and Fe^3+^-specific activity of the DNAzymes (e.g., sensor degradation under intracellular conditions), inactive DNAzymes (iErS) that contain single-nucleotide mutations that abolish DNAzyme cleavage activity [labeled in blue in Fig. 1 (A and B)] were used. Increase in the fluorescent signal of the Fe(II)-H5 (Fig. 1E) and the Fe(III)-B12 (Fig. 1F) DNAzyme sensors were measured in the presence of different concentrations of Fe^2+^ or Fe^3+^, respectively. Both sensors showed linear signal increase in response to increasing concentrations of their respective metal ions in a physiologically relevant range (Fig. 1, G and H, and fig. S22). In addition, our DNAzyme-based sensors show excellent selectivity for their cognate metal ion target over the different oxidation state of the same metal ion, as well as other biologically relevant metal ions (figs. S23 and S24).

### DNAzyme sensors monitor Fe^2+^ and Fe^3+^ simultaneously in living cells

**Fig. 2.**
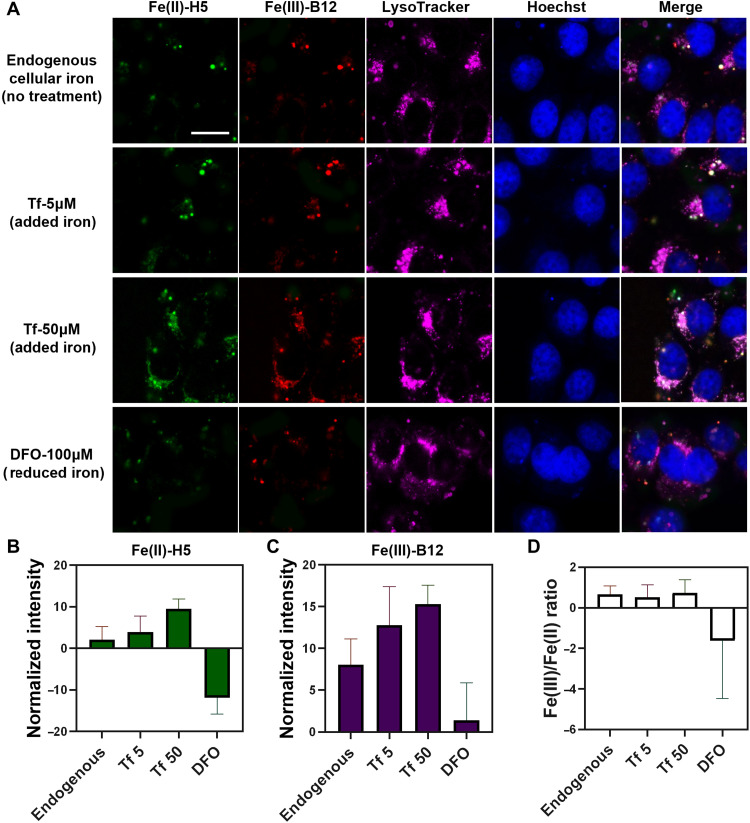
Simultaneous imaging of labile Fe^2+^ and Fe^3+^ in HepG2 cells. (**A**) HepG2 cells transfected with the Fe(II)-H5 (green) and the Fe(III)-B12 (red) DNAzyme sensors using PEI. Lysosomes are stained with LysoTracker Red and are shown in magenta. Cells were without any additional treatment to image endogenous (endo) iron, treated with 5 or 50 μM Transferrin (Tf) to increase lysosomal iron, or with 100 μM iron chelator DFO to decrease intracellular iron. (**B**) Statistical analysis of mean fluorescence intensity in LysoTracker-labeled region in (A) reveals that Fe^2+^ increased 1.9-fold when treated with 5 μM Tf and increased 4.6-fold when treated with 50 μM Tf, while Fe^2+^ decreased to barely detectable when treated with 100 μM DFO. (**C**) Statistical analysis of mean fluorescence intensity in (A) reveal that Fe^3+^ increased 1.6-fold when treated with 5 μM Tf and increased 1.9-fold when treated with 50 μM Tf and decreased around 5.7-fold when treated with 100 μM DFO. Cells were cultured in iron-deficient medium during the treatments. Error bars represent SEMs of the LysoTracker-labeled region in 20 different cells in each sample. Fluorescence intensity was normalized with ErS-iErS. Scale bar, 20 μm. (**D**) The Fe^3+^/Fe^2+^ ratio was calculated based on the mean fluorescence intensity of Fe^2+^ (B) and Fe^3+^ (C) in the Lysotracker region. When comparing with endogenous Fe^3+^/Fe^2+^ ratio in cells that were in the iron deficient media, no significant change in the Fe^3+^/Fe^2+^ ratio was observed in cells that were treated with different concentrations of Tf, and a 2.4-fold decrease of the Fe^3+^/Fe^2+^ ratio was observed in cells that were treated with 100 µM DFO.

Having demonstrated high selectivity for either Fe^2+^ or Fe^3+^ by our DNAzyme sensors under physiologically relevant concentrations, we explored imaging Fe^2+^ and Fe^3+^ in the endosomal-lysosomal system within living cells. This system is the entry point of the iron transport protein, Tf, which is the major iron import pathway in mammalian cells by binding to and importing extracellular Fe^3+^. In addition, the endosomal-lysosomal system is known to contain both Fe^2+^ and Fe^3+^ labile pools due to the presence of metalloreductase enzymes such as six-transmembrane epithelial antigen of the prostate 3 (Metalloreductase STEAP3), which reduces Fe^3+^ to Fe^2+^, thus enabling its further transport into the cytosol (*64*, *65*). To deliver the DNAzyme sensors into the endosomal-lysosomal system, we used polyethylenimine (PEI), which has previously been used to deliver nanosensors into the endosomal-lysosomal system successfully (*66*–*68*). The colocalization of the signal from DNAzyme sensors and LysoTracker Red, a lysosome marker (*69*), confirms efficient delivery of our sensors into lysosomes of the cells (Fig. 2A and fig. S25). The Pearson’s correlation coefficient between the iron pools and LysoTracker was around 50 to 60% (table S3), suggesting that although most of the iron signal was localized in lysosome, it was not evenly distributed, as not all the LysoTracker-positive regions showed iron signals. It is also possible that a small portion of Fe^2+^ or Fe^3+^ may be present in other parts of the cells. To introduce different iron concentrations in endo-lysosomes, the cells were incubated in an iron-deficient medium with increasing concentrations of Tf (*61*) or with DFO as an iron chelator to decrease the level of labile iron (*62*). Both the Fe(II)-H5 and Fe(III)-B12 DNAzyme sensors showed increase in fluorescent signal within lysosomes in response to increasing concentrations of Tf (Fig. 2). This observation is expected even for Fe^2+^ because of the reduction of Fe^3+^ to Fe^2+^ by endogenous metalloreductases in the endosomal-lysosomal system (*64*, *65*). However, the increase in the 5 μM Tf group was only 1.9-fold for Fe^2+^ and 1.6-fold for Fe^3+^, which may be due to the limited iron source in iron-deficient medium. To support this explanation, cells were also incubated in normal cell media, and around 4-fold increase was observed for both Fe^3+^ and Fe^2+^. In contrast, treating cells with DFO resulted in a 5.7- to 5.8-fold decrease of iron, as indicated by both DNAzyme sensors in iron-deficient media but only a 1.4- to 1.8-fold decrease of iron in normal media (Fig. 2 and figs. S26 to S28). Together, these results demonstrate that the Fe(II)-H5 and Fe(III)-B12 DNAzyme sensors can be used to image labile Fe^2+^ and Fe^3+^ in endo-lysosomes simultaneously. To the best of our knowledge, this is the first time that simultaneous imaging of Fe^2+^ and Fe^3+^ within the same location of a live cell has been demonstrated.

To quantify iron redox changes using our sensors, we calculated the Fe^3+^/Fe^2+^ ratio when treating the cells with Tf or DFO. As shown in Fig. 2D, we observed no significant change of the Fe^3+^/ Fe^2+^ ratio. This is probably because the Fe^3+^ imported by Tf is readily converted into Fe^2+^ because of the reducing environment inside the endosomal-lysosomal system (*65*). Moreover, when treating the cells with DFO, the Fe^3+^/Fe^2+^ ratio decreased 2.4-fold. This observation is consistent with previous report that DFO is a Fe^3+^ chelator (*62*), which can bind to Fe^3+^ and decrease its concentration.

The above results illustrated that Fe^2+^ and Fe^3+^ detection in an endo-lysosomal system is a result of guided delivery of the sensors using PEI. When we delivered the sensors using TurboFect, a delivery agent that has less subcellular localization effect, the sensor distributed more evenly inside of the cell and showed the presence of Fe^2+^ and Fe^3+^ in other parts of the cell, such as nucleus and cytoplasm (figs. S27 and S28). Thus, the delivery agents used for the sensors could influence the subcellular localization of the sensors. To calibrate our sensors with other iron sensors, we costained the Fe(II)-H5 DNAzyme sensor with FerroOrange, a commercially available Fe^2+^ sensor, and observed a similar increase in fluorescence intensity when adding excess amounts of iron (100 μM ferric ammonium citrate; fig. S29). However, less change in fluorescence intensity was observed in the presence of the iron chelator DFO. This difference suggests that FerroOrange is probably less sensitive in the presence of low concentrations of iron. We also found that the pattern of FerroOrange was similar but not the same as the signal from our Fe^2+^ sensor (fig. S29). Specifically, both sensors showed similar fluorescence distribution in cytoplasm in general, but our sensors showed an increased intensity for some of the cytoplasm regions. In addition, our sensor detected iron signal in the nucleus, which was not the case for FerroOrange. These differences are expected, as each sensor has its own cellular delivery efficiency, subcellular localization preference, sensitivity, and selectivity toward Fe^2+^. For example, FerroOrange was known to favor a localization in the Golgi and endoplasmic reticulum (*70*). To find out whether the difference in subcellular localization is responsible for the observed differences, we delivered the FerroOrange with PEI to help concentrate the sensor in the endosomal-lysosomal system and observed an enriched FerroOrange signal in the endosomal-lysosomal system (fig. S30). These results indicate that our sensor can show a similar trend as FerroOrange, yet it is more sensitive to iron.

### Increase in both Fe^3+^ and Fe^2+^ levels but decrease in Fe^3+^/Fe^2+^ ratio in ferroptotic cells

**Fig. 3.**
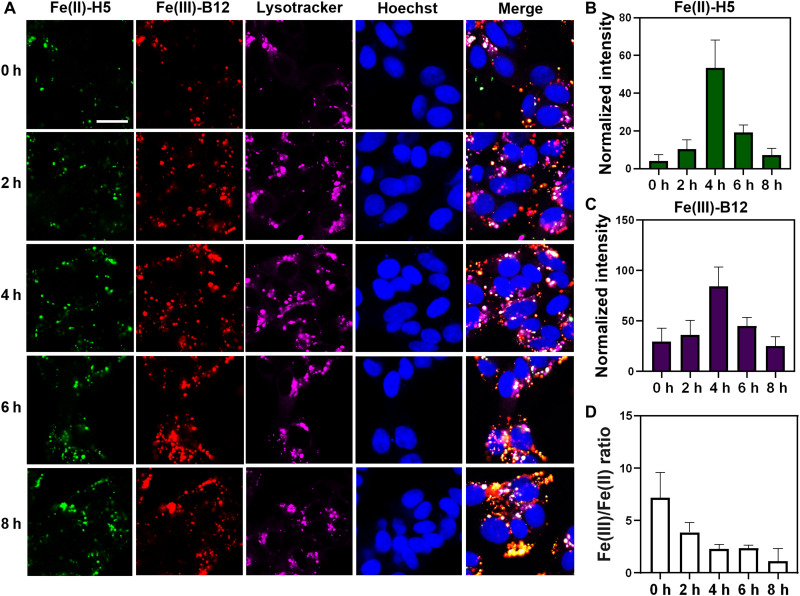
Ferroptosis triggers an elevation in the intracellular Fe^2+^ and Fe^3+^ pools with a decreased Fe^3+^/Fe^2+^ ratio. (**A**) Fe(II)-H5 (green) and Fe(III)-B12 (red) DNAzyme sensors detecting labile pools of Fe^2+^ and Fe^3+^ simultaneously during RSL3-induced ferroptosis at different time points. LysoTracker Red and Hoechst 33258 were used to label lysosomes (magenta) and nucleus (blue), respectively. (**B** and **C**) Statistical analysis of normalized fluorescence intensity in the LysoTracker-labeled region reveals an increase of Fe^2+^ (B) and Fe^3+^ (C) within the first 4 hours and a decrease of Fe^2+^ and Fe^3+^ within 4 to 8 hours of RSL3-induced ferroptosis in the endosomal-lysosomal system. (**D**) The Fe^3+^/Fe^2+^ ratio decreases during ferroptosis. Scale bar, 20 μm. h, hours.

Next, we investigated whether our DNAzyme-based sensors can detect changes in iron redox states by using the Fe(II)-H5 and Fe(III)-B12 sensors to monitor Fe^2+^ and Fe^3+^ and their conversion during ferroptosis, which can be triggered by lipid peroxidation related to excess amounts of iron and its resulting Fenton reaction (*5*, *71*). To induce ferroptosis in our model system, we incubated HepG2 cells with RAS-selective lethal 3 (RSL3) (*72*, *73*), which is a well-known ferroptosis inducer. RSL3 inhibits glutathione peroxidase 4 (GPX4), which eliminates phospholipid peroxides, and thus allows lipid peroxides to accumulate because of iron-mediated Fenton chemistry, triggering ferroptosis-induced cell death without directly modulating total iron levels (*74*). The 3-(4,5-dimethylthiazol-2-yl)-2,5-diphenyl-2*H*-tetrazolium bromide (MTT) assay (*75*) showed RSL3-induced cell death over 8 hours (fig. S31). By monitoring the cells in this 8-hour window of ferroptosis, we observed a rapid increase of both Fe^2+^ (Fig. 3, A and B, and figs. S32 and S33A) and Fe^3+^ (Fig. 3, A and C, and figs. S32 and S33B) levels in the LysoTracker-labeled region within the first 4 hours of ferroptosis. These results indicate a rapid generation of labile Fe^2+^ and Fe^3+^ within lysosomes during the initiation stage of the ferroptosis. We observed a larger increase of Fe^2+^ (2.3-fold increase from 0 to 2 hours and 4.3-fold increase from 2 to 4 hours) than that of Fe^3+^ (2.8-fold increase of from 0 to 4 hours). As a result, we observed a continuous decrease of the Fe^3+^/Fe^2+^ ratio during ferroptosis (Fig. 3D), which indicates that the reduction of Fe^3+^ to Fe^2+^ could serve as a source for labile Fe^2+^ in endo-lysosomes. After 4 hours, both iron levels decreased, indicating that, in the later stage of ferroptosis, the labile iron pools are being depleted from the endo-lysosome. These results demonstrate that simultaneous monitoring of Fe^2+^ and Fe^3+^ levels by our DNAzyme-based sensors can provide insights into unexplored roles of labile Fe^2+^ and Fe^3+^ during ferroptosis.

### Elevated iron redox levels accumulate in amyloid β plaque regions in AD mouse brain

**Fig. 4.**
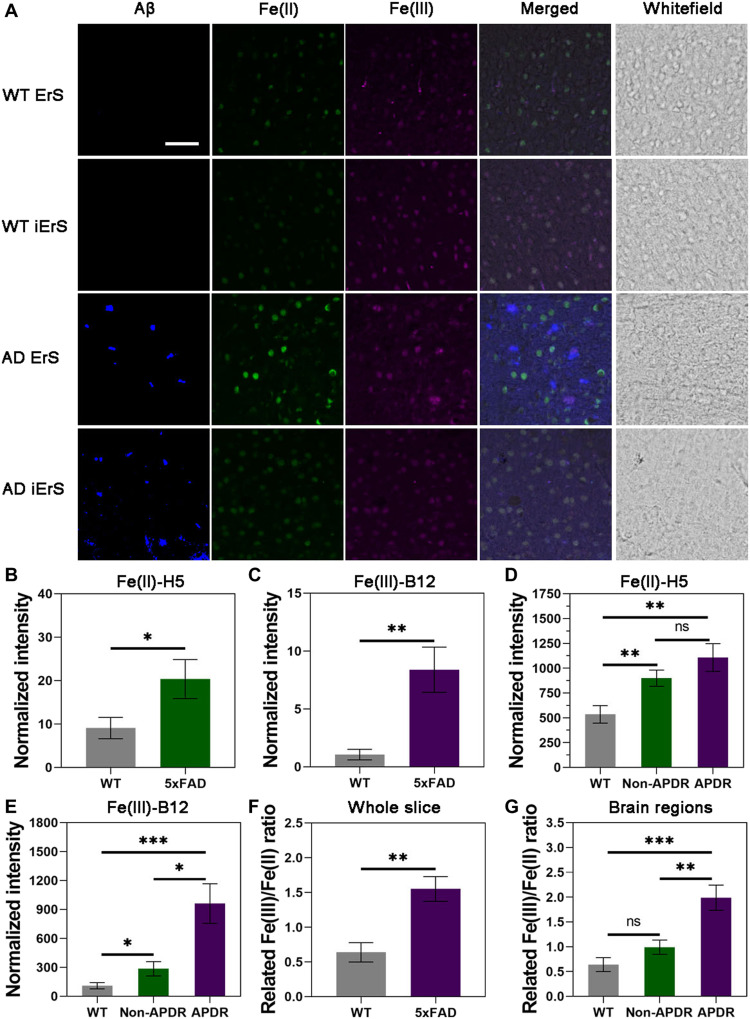
Fe^3+^/Fe^2+^ ratio increases in brain regions containing amyloid plaques. (**A**) Both Fe^2+^ and Fe^3+^ were monitored in 11-month-old mouse brain slices that immunostained with the HJ3.4 antibody, which labels immunoreactive Aβ plaques, to trace the colocalization between iron and Aβ plaques in mouse brains. ErS, the active version of iron sensors containing the enzyme (E) strand and the substrate strand with the ribonucleotide as the cleavage site (rS); iErS, the inactive DNAzyme sensor control, which contains a point mutation in the enzyme strand that renders the DNAzyme inactive in the presence of Fe^2+^ or Fe^3+^. The signal from iErS is considered as background signal that is caused by auto fluorescence or Fe^2+^- or Fe^3+^-independent cleavages (**B** and **C**) Statistical analysis of fluorescence intensity in (A). Fluorescent signals were normalized by subtracting the background detected with inactive DNAzyme sensors. Both Fe^2+^ (B) and Fe^3+^ (C) levels were elevated in 5xFAD mice when compared with wild-type (WT) controls. (**D**) When monitoring the distribution of Fe^2+^ and Fe^3+^ and comparing with WT mouse brains, Fe^2+^ level was elevated 2.1-fold in the cortex regions with Aβ plaques (APDR) and was elevated 1.7-fold in the surrounding cortex regions that did not show Aβ plaque deposition signal (non-APDR). (**E**) Fe^3+^ increased more (8.7-fold) in APDR than the non-APDR (2.6-fold). (**F**) The Fe^3+^/Fe^2+^ ratio showed increase in whole-brain slices of WT and 5xFAD mice. (**G**) Most of the increased Fe^3+^/Fe^2+^ ratio was in the APDR but not non-APDR in 5xFAD mouse cortex. Scale bar, 50 μm. Analyzed with paired Student’s *t* test. *P < 0.05, **P < 0.01, and ***P < 0.001. ns, not significant.

We next investigated the ability of our DNAzyme-based sensors to monitor labile iron levels in a more complicated biological model. Increasing evidence has linked ferroptosis to AD because elevated iron levels were also observed in AD patients’ brains, and thus, excessive accumulation of iron is considered as a risk factor in the development of AD (*76*). However, limited information on spatial distribution of Fe^2+^ and Fe^3+^ and their redox dynamics in AD pathogenesis is available, partly because of the limitations of existing iron sensors. To address this unmet need, we further applied our sensors to image labile iron in brain slices of AD mice to gain insights into the relationship between iron redox activity and AD progression. Fe^2+^ and Fe^3+^ were detected simultaneously in the cortex region and compared between 11-month-old wild-type (WT) mice and 5xFAD mice that express mutant humanized amyloid precursor protein and mutant human presenilin 1 (PSEN1) transgenes, which are commonly used as an AD model (Fig. 4A) (*77*). A 2.3-fold increase in the Fe^2+^ level (Fig. 4B) and a 7.9-fold increase in the Fe^3+^ level (Fig. 4C) was observed in whole-brain slices from 5xFAD mice when compared to the WT controls, indicating that Fe^3+^ accumulates in the AD mouse brains more than Fe^2+^. To understand the involvement of iron redox in the AD pathogenesis, we further investigated the distribution of iron oxidization states in relation to amyloid β (Aβ) formation, which accumulates in AD brain and contributes to neurotoxicity (*77*), by comparing the fluorescence intensity of our Fe^2+^ and Fe^3+^ DNAzyme-based sensors in the cortex regions that have either Aβ plaques or not. Both iron sensors were costained with HJ3.4 antibody, which labels immunoreactive Aβ plaques (*78*). Fe^2+^ was increased by 2.1-fold in and surrounding the cortex regions that have Aβ plaque deposition [called Aβ plaque deposition region (APDR)], while Fe^2+^ was elevated by 1.7-fold in the surrounding cortex regions that did not show Aβ plaque deposition (called non-APDR; Fig. 4D). The statistical analysis showed that the increase in the level of Fe^2+^ in both APDR and non-APDR was significant when compared with the WT controls. However, there was no significant difference between APDR and non-APDR Fe^2+^ levels, which suggests a similar elevation of Fe^2+^ levels in the cortex regions of AD mouse. In contrast, we observed a 2.6-fold and an 8.7-fold increase in the levels of Fe^3+^ in non-APDR and APDR, respectively, when compared with their WT controls (Fig. 4E). These observations suggest that the accumulation of the oxidized state of iron in AD brain is preferentially enriched in APDR. Because of the nonhomogenous distribution, such a closer observation and analysis instead of a brief comparison on whole-brain slices is necessary to understand how iron redox correlates with AD models.

To understand the role of iron redox cycling in AD progression, we further used our sensors to compare the relative Fe^3+^/Fe^2+^ ratios based on the fluorescence difference between 5xFAD mice and WT mouse brains. When performing the comparison with whole-brain slices, we observed a 2.4-fold increase of Fe^3+^/Fe^2+^ ratio in AD mouse cortex when compared with its WT controls (Fig. 4F). We then analyzed the spatial information of these redox changes in detail and found a 1.6-fold increase in the relative Fe^3+^/Fe^2+^ ratio in non-APDR and a 3.1-fold elevation in the relative Fe^3+^/Fe^2+^ in APDR of 5xFAD mouse brains when compared with WT mouse brains (Fig. 4G). This observation reveals that oxidative stress in AD brains is associated with aggregation of Aβ. Although Fe^3+^ aggregates with Aβ plaques and could reduce to Fe^2+^ to serve as one of the sources for generating reactive oxygen species (*79*–*81*), the Fe^3+^/Fe^2+^ level is still high around Aβ plaque regions, suggesting the potential existence of a continuous source of Fe^3+^ diffusing out from surrounding cells and/or proteins, or a different mechanism of Aβ plaque/ferric ion–related reactive oxygen species (ROS) generation. From these results, we have validated that both the Fe(II)-H5 and Fe(III)-B12 DNAzyme sensors can monitor the dynamic of Fe^2+^ and Fe^3+^ interconversion and provide spatiotemporal information that will gain deeper insights into iron-related diseases such as AD.

## DISCUSSION

Visualizing different oxidation states of redox-active metals provides valuable information for understanding the role of metal redox in regulating biological processes and human health. Despite this importance, current imaging tools are limited, and simultaneous imaging of different oxidation states of the same metal ion has yet to have been reported. To overcome these limitations, we have isolated DNAzymes that are highly specific for either Fe^2+^ or Fe^3+^ through in vitro selection. Upon further characterization and conversion into fluorescent sensors using the catalytic beacon strategy, we demonstrate the ability to use these DNAzyme-based turn-on fluorescent sensors for simultaneous detection of two oxidation states of iron in living cells and in brain slices of transgenic AD mice.

Recently, it has been demonstrated that ferroptosis increases the total labile iron pool that contains both Fe^2+^ and Fe^3+^ due to degradation of the Fe^3+^-storage protein ferritin in lysosomes, causing labile iron overload (*82*, *83*). We monitored the iron distribution during ferroptosis and observed a similar increase of total iron. Our sensors provided the first piece of information for iron redox changes during ferroptosis (Fig. 3). An increase in the overall labile iron pool was observed within 4 hours when inducing ferroptosis with RSL3. After 4 hours of ferroptosis, a decrease of both Fe^2+^ and Fe^3+^ was observed, which suggests a depletion of labile iron from the endosomal-lysosomal system in the later stage of ferroptosis. This reduction could be induced by STEAP3 or other endogenous metalloreductases, while the initial increase of Fe^3+^ is possibly from the release of ferritin-bound Fe^3+^ (*65*). In addition, a decrease in the Fe^3+^/Fe^2+^ ratio was observed over time, and such an observation reveals a potential role of iron redox cycling, which may serve as a source of oxidative stress, during ferroptosis. This conclusion is consistent with oxidative stress accumulation during ferroptosis, an iron-dependent cell death pathway (*84*).

As a key player in redox biology, iron is also involved in the generation of reactive oxygen species from Aβ (*79*). By using our DNAzyme-based sensors, we observed elevated iron signals that colocalized with aggregated Aβ plaques (Fig. 4, A, D, and E). The observations on both total iron increase and the distribution patterns are consistent with earlier reports demonstrating increase in the level of iron in AD brain slices stained with potassium ferricyanide/ferrocyanide or as detected by magnetic resonance imaging (MRI) (*18*, *22*, *85*). Although these previous works reported similar increases in cerebral iron accumulation around Aβ, here, we are able to visualize both Fe^2+^ and Fe^3+^ simultaneously in single brain slices, allowing observation of the spatial relationship between the two oxidation states. In addition, our iron-specific DNAzyme-based sensors allow us to obtain spatial information about iron redox ratios, revealing a significant increase in the Fe^3+^/Fe^2+^ ratio surrounding amyloid plaque regions but not in other brain regions (Fig. 4G). Our data suggest that not only total iron but also iron redox cycling is involved in the progression of AD. Combining these data with our observation that both Fe^2+^ and Fe^3+^ levels increased around Aβ plaque regions and suggests a potential role of Aβ plaques in accumulating Fe^3+^ over Fe^2+^ from surrounding cells and/or proteins in AD mouse brains. The elevated levels of iron surrounding Aβ plaques might be derived from labile iron pools that contribute to the formation of iron-Aβ adducts or transchelation of iron from ferritin by Aβ plaques during AD progression due to the potential interaction between iron and Aβ plaques, instead of simply changing the oxidation states between different forms of iron (*86*, *87*). However, it is unknown whether the dysregulated iron is involved in amyloid plaque formation, or this is a secondary effect of amyloid plaque formation in this mouse model. Overall, our data demonstrate that our DNAzyme-based iron sensors can provide unique and powerful tools for studying the intracellular dynamics of iron redox states and have the potential to open new avenues to further investigate different biological processes that involve redox metal ions and understand their roles in several neurodegenerative diseases such as AD.

## MATERIALS AND METHODS

### Materials

#### DNA sequences

All DNA was ordered from Integrated DNA Technologies (IDT). Modifications are indicated with IDT’s modification codes (see table S4).

#### Buffers

Fe^2+^ buffer contains 50 mM bis-tris and 400 mM NaCl at pH 7.0 (adjusted with HCl). Fe^3+^ buffer contains 40 mM sodium acetate, 5 mM bis-tris, and 200 mM NaCl at pH 5.5 (adjusted with HNO_3_). Universal Fe buffer contains 20 mM sodium acetate, 5 mM bis-tris, and 200 mM NaCl at pH 6.0.

### In vitro selection of Fe^2+^-dependent DNAzymes

The initial pool used for all four different selection conditions was identical, using a randomized N50 region flanked by two primer-binding regions, of which one contained the single riboadenosine to serve as a cleavage site. A P2-iSp primer contained a hexaethylene glycol spacer (Spacer-C18, from IDT) modification, which stops Taq polymerization reaction from further extension, was used during the polymerase chain reaction (PCR) amplification step. The internal C18 spacer is followed by AACAACAACAACAAC, which results in production of the antisense strand with 15 nucleotides longer than the sense strand (DNA random pool). Therefore, single-stranded DNA random pools were separated from the antisense strand using denaturing polyacrylamide gel electrophoresis (PAGE).

The initial random pool for the first selection round was generated in two steps using a PCR thermocycler (C1000 Touch Thermal Cycler from Bio-Rad Laboratories Inc.). PCR1 was carried out in 96 PCR tubes with 0.1 μM IDT DNA template in three steps. In the first step, 0.1 μM primer P2-iSp was added to the PCR mixture containing 0.1 μM DNA template to undergo two cycles of extension. The second step was carried out by the addition of 0.15 μM primer P1 and two more extension cycles. Last, in the third step, 0.9 μM primer P2-iSp was added, and 10 cycles of extension were carried out. PCR2 was conducted by the addition of 1 μM primer P3 (for incorporation of the RNA base) and 0.1 μM primer P2-iSp, followed by 10 cycles of amplification. Before the PCR2 reaction was performed, 2 μl of [α-^32^P]-dATP (PerkinElmer) was added to label the DNA strands. The PCR products were then precipitated with 10% of a 3 M sodium acetate solution, at pH 5.2, and 2.7× volume of cold ethanol. The samples were stored at −80°C for at least 1 hour and then centrifuged, washed, and lyophilized. Note that subsequent PCR amplifications, which were used to amplify selected DNA at the end of each selection round, were slightly different. In those reactions, PCR1 was carried out in a single step with 1 μM P2-iSp and P2 primers, followed by PCR2 using 5% of the PCR1 reaction as DNA template with 0.1 μM primer P2-iSp and 1 μM P3.

Ethanol-precipitated PCR products were dissolved in water, and an equal amount of stop buffer was added before loading samples on the gel. The stop buffer contained 8 M urea, 50 mM EDTA, and 1× TBE (tris, boric acid, and EDTA). The reaction products were purified using a 10% denaturing PAGE gel, with the use of 1× TBE as the running buffer. The PCR product was run on the PAGE gel alongside DNA size markers corresponding to the cleaved (87-mer) and intact (110-mer) pool. The gel was then covered with a plastic wrap; a radioactive triangle location marker was placed on top, and the gel was exposed to a phosphorimager cassette. After imaging the exposed film, bands that corresponded to the 110-mer marker on the gel was excised, crushed, and extracted with a solution containing 10 mM tris, 0.1 mM EDTA, and 300 mM sodium chloride (extraction buffer). Gel particles were frozen over 10 min at −80°C and thawed in a room temperature water bath for at least 5 min to improve the extraction process. The solution was centrifuged at 10,000*g* for 1 min to obtain a gel-free DNA in the extraction buffer. DNA samples were ethanol-precipitated using the aforementioned procedure.

The dried pools acquired from initial pool generation for the first selection round (and for PCR amplifications of subsequent selection rounds) were redissolved in 1× selection buffer and incubated with a desired concentration of Fe^2+^ for positive selections or a mixture of competing divalent metal ions (for counter selections) for 18 hours. Fe^2+^ concentration and incubation time at each round of the selection are indicated in tables S5 and S6. An initial negative selection was carried out before round 1 by incubating the DNA pool in selection buffer in the absence of divalent metal ions for 24 hours. Counter selections were carried out by incubating the DNA pools with 1 mM Mn^2+^, Cd^2+^, Zn^2+^, and Co^2+^ and 0.2 mM Pb^2+^ in selection buffer over 18 hours. Overall, four different selection conditions were carried out and each condition named with a letter (E to H; see table S1). After negative and counter selection steps, uncleaved DNA pools were PAGE-purified and used for the subsequent positive selection. Cleaved DNA produced in each positive selection was PAGE-purified and used as a PCR template for a subsequent round of the selection. The stringency of positive selections was gradually increased by decreasing the reaction time and decreasing Fe^2+^ concentration as the selection progressed (tables S5 and S6). All selection reactions were quenched by the addition of the stop buffer (50% final volume). All PAGE purifications were carried out using a 10% denaturing gel alongside the DNA size markers used earlier.

### In vitro selection of Fe^3+^-dependent DNAzymes

Similar to the selection of Fe^2+^-DNAzymes, in vitro selection for Fe^3+^-dependent DNAzymes was carried out using a denaturing PAGE-based purification method to separate cleavage products from uncleaved pools based on their size difference. In vitro selection was performed at pH 5.5 using rG as the cleavage site. Moreover, two different DNA pools with random region size of 35 or 50 nucleotides were used. The initial selection pools were generated through single linear PCR amplification. The PCR templates, synthesized by IDT, were designed with the goal of eliminating the need for multiple PCR amplifications to generate the initial sequence pools. DNA pools were generated by linear amplification of 360 pmol of the template mixed with 3.6 nmol of P3 primer in 90 PCR tubes (40 μl each), followed by 10 thermal cycles to complete the pool generation (40 s at 94°C, 1.25 min at 53°C, and then 1.1 min at 72°C). In the generation of each pool, 4 μl of [α-^32^P]-dATP was used to internally label PCR products with ^32^P. Amplification of the selected pools after each positive selection round was carried out through two PCR reactions. In addition to the template and primers, each PCR reaction included Taq DNA polymerase (0.1 U/μl; NEB), 1.5 mM MgCl_2_, 50 mM KCl, 10 mM tris-HCl (pH 8.3 at 25°C), and each deoxynucleoside triphosphate (dNTP) at 0.2 mM. Note that all parameters of different PCR reactions were optimized before the initiation of the in vitro selection. This optimization process was required to minimize production of side products, obtain clean products, and increase yield of the correct PCR product. DNA pools were PAGE-purified using the same protocol described in the “In vitro selection of Fe^2+^-dependent DNAzymes” section.

Dried pools were dissolved in 1× selection buffer and incubated for 24 hours (negative selection). This step was carried out before all positive selection steps unless stated. Negative selection steps were carried out to remove nonspecific cleavage that may occur in an Fe^3+^-independent manner. After the negative selection, uncleaved pools were PAGE-purified, extracted from gel, ethanol-precipitated, and dried to be used for positive selection. Dried pools were dissolved in 1× selection buffer and mixed with desired concentration of Fe^3+^ (for positive selections) for a certain period of time (table S7). To initiate selection, Fe^3+^ was dissolved in selection buffer to make 2× Fe^3+^ stock solution. Then, equal volume of DNA samples were mixed with the 2× Fe^3+^ stock solution. The 2× Fe^3+^ solutions were prepared right before the positive selection. All positive selections were carried out in the dark by covering tubes with aluminum foil to prevent unwanted light-induced DNA cleavage by Fe^3+^. After round 4, each of the in vitro selection experiments carried out with the rG cleavage site at pH 5.5 were branched into two conditions by continuing or not continuing negative selection steps (see table S7). Cleaved DNA obtained in each positive selection was PAGE-purified and used as template for PCR amplification reactions to generate DNA pools for the next round of the selection. Stringency of positive selections was gradually increased by decreasing both the reaction time and Fe^3+^ concentration (table S7). All selection reactions were quenched with an equal volume of stop buffer. All PAGE purifications were carried out using a 10% gel alongside the DNA size markers used earlier.

### Cloning and sequencing

On the basis of (i) the cleavage activity of the selected pools, (ii) results obtained in control experiments, and (iii) the activity assays carried out with different Fe^2+^ or Fe^3+^ concentrations, the most active pools with lowest background activity were chosen for cloning and sequencing. Cloning was carried out using PCR products with primers with no ribonucleotide cleavage site or Taq stopper. The same PCR reactions were carried out using selection primers to control activity of the species used for cloning. Negative PCR controls, with no template, were performed to assure that the observed amplifications were not caused by a contamination.

DNA sequences obtained from sequencing aligned on the basis of their sequence similarity for each individual pool. Sequence identity of the thermodynamically stable DNA tetraloop, which was engineered in the design of the random pools, remained intact in more than 96% of the obtained clones for the Fe^3+^-dependent selection. Among 149 individual sequences obtained from Fe^2+^-dependent selections, only 38 of them contain the intact tetraloop, with the rest of the clones having at least one mutation in this region. Few sequences were identified with mutations in their primer regions. Conservation of the stable tetraloop suggests that formation of this stable structure did not interfere with catalytic activity of the evolved sequences, while relatively high variation in the tetraloop region might imply that formation of this stable structure was not in favor of forming catalytically active structures. Because this region in the Fe^2+^ pool was not part of the PCR primers and considering the error rate of Taq DNA polymerase, a potential selection pressure might have caused evolution of species without the stable tetraloop over several selection rounds. The presence of the tetraloop in the active sequences can help in the prediction of active secondary structures. The sequence similarity of obtained sequences was represented using sequence similarity networks, originally used for organizing sequence similarity of protein sequences.

### In vitro activity assays of Fe(II)-H5 and Fe(III)-B12 DNAzymes

In vitro activity assays were performed by incubating a solution of the DNAzymes with the indicated amount of metal solution (typically in a 1:1 volume ratio) and measuring the fluorescence change over time with a fluorometer. Metal stocks were prepared from ferric nitrate [Fe(NO_3_)_3_] and ferrous chloride (FeCl_2_) dissolved in HNO_3_ or HCl, respectively, which were then diluted into the relevant buffer. Both the metal solutions and the DNAzyme solutions were prepared in the relevant buffers so that there was no effect from different buffers mixing during the reaction, and their pH was checked to make sure that the solutions remained at the desired pH (especially important for the higher metal concentrations). For aerobic conditions, a SpectraMax M2 multidetection reader was used from the Roy J. Carver Biotechnology Center at the Metabolomics Center. For anaerobic conditions, a DeNovix QFX portable fluorometer was used inside of an anaerobic glove box. In addition, the metal selectivity tests were all performed with the DeNovix QFX fluorometer for consistency. For the SpectraMax M2, excitation at 633 nm and emission at 665 to 750 nm were used. For the DeNovix QFX, excitation at 470 nm and emission at 514 to 567 nm were used for the Fe(II)-H5 DNAzyme, and excitation at 635 nm and emission at 665 to 740 nm were used for the Fe(III)-B12 DNAzyme. Before the assay, the enzyme and substrate strands were annealed together using a water bath >65°C for 5 min and then cooled to room temperature for 30 min. The fluorescence intensity in the presence of different metal ions was normalized to the fluorescent signal without addition of divalent or trivalent metal ions (i.e., just buffer) at the 30-min time point, for a direct comparison.

### Cell culture, sensor delivery, and colocalization study

Iron-deficient buffer was prepared by S. McMasters of the University of Illinois School of Chemical Sciences Cell Media Facility as a standard preparation of minimum essential medium (MEM) but without fetal bovine serum (FBS) or any added iron (from Tf) to remove any potential contaminating iron species. HepG2 cells were purchased from the American Type Culture Collection (HB-8065), and was cultured in Dulbecco’s modified Eagle’s medium (DMEM) with adding 10% FBS, penicillin (100 U/ml), and streptomycin (100 U/ml), and in a 5% CO_2_, 37°C incubator. A hemocytometer was used to determine cell density.

For delivery of DNAzyme sensors using PEI (*66*, *67*), the corresponding Fe(II)-H5-ErS, Fe(II)-H5-iErS, Fe(III)-B12-ErS, and Fe(III)-B12-iErS (final concentration of 10 μM) were mixed in the Fe^2+^ buffer and Fe^3+^ buffer separately. The mixtures were annealed at 95°C for 5 min and stored at room temperature to allow full hybridization. PEI (25 kDa) was dissolved in water at 1 mg/ml. For the active enzyme group, 2 μl of PEI was mixed and incubated with 2 μl of 10 μM Fe(II)-H5-ErS and Fe(III)-B12-ErS for 30 min in iron-deficient medium to allow the formation of PEI-DNA complexes (PEI-ErS) with the optimal N/P ratio (moles of amine, N, from the cationic polymer to moles of phosphate, P, from the DNA) as suggested previously (*66*). For the inactive enzyme group, 2 μl of PEI was mixed and incubated with 2 μl of 10 μM Fe(II)-H5-iErS and Fe(III)-B12-iErS in iron-deficient medium for 30 min to allow the formation of PEI-DNA complexes (PEI-iErS). The normal cell medium was replaced with iron-deficient medium before the PEI-ErS or PEI-iErS was added to the cells grown in the plates.

tAfter incubating HepG2 cells with PEI-ErS or PEI-iErS complex for 4 hours, cells were washed with phosphate-buffered saline (PBS) twice to remove excess amounts of complexes in medium. Then, the cells were stained by Hoechst 33258 for 15 min. Images were obtained using a Zeiss LSM 880 confocal microscope at 63× oil objective lens with numerical aperture 1.40 and immersion medium Immersol 518 F (Zeiss) at the UIUC IGB Core Facilities. Florescence emission of Hoechst 33258 was measured over 450 to 500 nm with excitation at 401 nm. Fe(II)-H5 was excited by 488 nm and measured over 500 to 550 nm, and Fe(III)-B12 was excited at 633 nm and measured over 640 to 700 nm. A laser power of 67% and pinhole size of 1 atomic unit (AU) were used for the cellular images. Acquired images were analyzed by ImageJ using JACoP (*88*, *89*). No background subtraction, *Z*-stacks, Gaussian blur filter, or change thresholds were performed for visualizing the figures. For cell fluorescence quantification, we quantified the average fluorescence signals intensity in five cells (for TurboFect transfected cells) or five LysoTracker-labeled regions in different cells (for PEI transfected cells), per picture and three pictures per group with Image J (*88*), for the statistical comparison between groups.

FerroOrange was used at the final concentration of 1 μM, following the standard protocol from Sigma-Aldrich. When delivering FerroOrange with PEI, the same final concentration of FerroOrange was mixed with 2 μl of PEI and incubated at room temperature for 30 min before transfecting the cells. When delivering the DNAzymes with TurboFect, 100 pmol of DNAzyme S strand was annealed with 110 pmol of E strand in cells with 5 mM bis-tris, 40 mM sodium acetate, and 200 mM sodium chloride (pH 5.5) and then diluted with 98 μl of Opti-MEM and 1 μl of TurboFect transfection reagent. The mixture was incubated at room temperature for 20 min and incubated with cells for 4 hours. Afterward, the cells were washed with 1× Hanks’ balanced salt solution (HBSS) and stained with Hoechst 33342 before imaging.

### Simultaneous imaging of labile Fe^2+^ and Fe^3+^ in living cells

HepG2 or HeLa cells were cultured in glass-bottom dishes until about 70% confluence. The cells were pretreated with iron-deficient MEM containing either 100 μM DFO, 100 μM ferric ammonium citrate, 5 μM holo-Tf, or 50 μM Tf for 4 hours, and then, the medium was replaced with an iron-deficient MEM containing PEI-ErS or PEI-iErS complexes or Opti-MEM containing 1 μl of TurboFect transfection reagent as described in the “Cell culture, sensor delivery, and colocalization study” section for 4 hours. Before imaging, the cells were stained by LysoTracker Red and Hoechst 33258 for 15 min. After washing with PBS, the cells were incubated in iron-deficient MEM or regular DMEM, respectively, during the imaging.

### Ferroptosis-induced fluctuations of labile Fe^2+^ and Fe^3+^ pools

HepG2 cells were treated with 1 μM RSL3 in normal MEM at different time points (0, 2, 4, 6, 8, and10 hours). Then, the cell medium was replaced by iron-deficient MEM containing PEI-ErS or PEI-iErS and incubated in a 37°C, 5% CO_2_ incubator for another 4 hours. Before imaging, the cells were stained by LysoTracker Red and Hoechst 33258 for 15 min. After washing with PBS, the cells were incubated in iron-deficient MEM during the imaging.

To measure the cytotoxicity of RSL3 to HepG2 cells, a standard MTT assay was used. HepG2 cells were seeded at a density of 15,000 cells per well in 96-well plates. When the cells grew to 80% confluence, the medium was replaced by the normal MEM containing 1 μM RSL3 at different time points (0, 2, 4, 6, 8, and 10 hours). Afterward, the cell medium was replaced by the iron-deficient MEM containing PEI-ErS or PEI-iErS, incubated in a 37°C, 5% CO_2_ incubator for another 4 hours. The absorbance at 570 nm was measured with a SpectraMax M2 microplate reader to obtain the MTT assay readings. The untreated cells were set as a blank control for normalization.

### Fe^2+^ and Fe^3+^ detection in mouse brain slices

All animal studies were performed with the approval of the Institutional Animal Care and Use Committee (IACUC) of the University of Illinois at Urbana-Champaign (protocol number 22094) and the IACUC of the University of Texas at Austin (protocol number AUP-2021-00295). Eleven-month-old 5xFAD mice (RRID: MMRRC_034840-JAX) and WT mice (B6SJLF1/J) of the same sex were used and compared. To obtain the brain slices, after deep anesthesia, the mice were perfused with saline, followed by paraformaldehyde fixation. The mouse brain was isolated and fixed in 4% paraformaldehyde for 24 hours at 4°C and then embedded in 30% sucrose in PBS for another 3 days. Coronal brain sections (section thickness, 50 μm) were obtained by microtome section and stored in cryoprotectant at −20°C before staining. Ten brain slices from three individuals were stained with technical repeats for each group. The brain sections were rinsed with PBS three times for 5 min each and then blocked with 2% bovine serum albumin (BSA) in PBS for 10 min. To visualize Aβ(1-13), brain slices were incubated with CF350-conjugated HJ3.4 antibody (*78*), which was prelabeled with the Mix-n-Stain Antibody Labeling Kit (Sigma-Aldrich), with 1:500 dilution in blocking solution for 1 hour and then washed with 2% BSA in PBS for 4 min, followed by another wash in PBS for 5 min to remove extra antibody and nonspecific signaling. To image Fe^2+^ and Fe^3+^ simultaneously, 2 μM DNAzyme enzyme strand and 2.2 μM DNAzyme substrate strand for both iron oxidization states were annealed separately in 5 mM bis-tris (pH 5.5), 40 mM sodium acetate, and 200 mM NaCl buffer (bis-tris-acetate buffer) by incubation at 95°C for 5 min and then slowly decreased to room temperature. After annealing, the Fe(II)-H5 and Fe(III)-B12 sensors were mixed in a 1:1 ratio to generate the sensor mix. The mouse brain sections prestained with HJ3.4 antibody were rinsed with the bis-tris-acetate buffer twice for 5 min each and then incubated in the sensor mix for 30 min. After incubation, the brain slices were rinsed once in the bis-tris-acetate buffer and mounted with Fluoromount-G mounting medium (SouthernBiotech, Birmingham, AL, USA). Images were taken with a Nikon spinning disk confocal or Zeiss LSM 710 confocal microscope with a 20× objective with 405-, 488-, and 640-nm channels. Afterward, images were processed and quantified with ImageJ as follows: To enable a direct comparison between groups without interference from a potential difference in background signaling between treatments, as indicated by the point-mutated inactive sensor (iErS), we normalized the fluorescent intensity of our sensor with iErS groups by subtracting the average fluorescence intensity in the imaging area or the regions of interest (APDR or non-APDR regions) in the pictures of correlated iErS group from the ErS group with the same treatment. Colocalization study and quantification of fluorescence intensity for APDR and non-APDR regions were performed by marking the region of interests on the basis of HJ3.4 staining. Five spots including four corners and the center of each image were analyzed. Three images were taken for each group.

### Data processing and statistical analysis

To have a direct comparison of the iron amount between groups, we normalized the background signals by subtracting the fluorescence signals in iErS groups from the ErS groups (*F* − *F*_0_). No *Z*-stacks or change thresholds were used for statistical analysis. Graphs were plotted with GraphPad (GraphPad Software Inc.) or Origin (OriginLabs Corporation) and correspond to a single experiment. Bars represent means ± SEM. Two-tailed distribution and two-sample equal variance *t* test were used for analyzing significance. Dixon’s *Q* tests were performed to identify and exclude outliners. *P < 0.05, **P < 0.01, and ***P < 0.001. All experiments have three or more biological replicates, which showed the same conclusion. The presented data described the quantification from single representative replicates from those biological replicates.

## Supplementary Materials

### This PDF file includes:

Figs. S1 to S33 Tables S1 to S7 References

## Notes

View/request a protocol for this paper from *Bio-protocol*.

## References

[1] D. Galaris, A. Barbouti, K. Pantopoulos, Iron homeostasis and oxidative stress: An intimate relationship. Biochim. Biophys. Acta Mol. Cell Res. 1866, 118535 (2019). 31446062 10.1016/j.bbamcr.2019.118535

[2] N. Kim, H. J. Lee, Redox-active metal ions and amyloid-degrading enzymes in Alzheimer’s disease. Int. J. Mol. Sci. 22, 7697 (2021). 34299316 10.3390/ijms22147697 PMC8307724

[3] E. L. Que, D. W. Domaille, C. J. Chang, Metals in neurobiology: Probing their chemistry and biology with molecular imaging. Chem. Rev. 108, 1517–1549 (2008). 18426241 10.1021/cr078203u

[4] K. P. Carter, A. M. Young, A. E. Palmer, Fluorescent sensors for measuring metal ions in living systems. Chem. Rev. 114, 4564–4601 (2014). 24588137 10.1021/cr400546e PMC4096685

[5] J. M. Braughler, L. A. Duncan, R. L. Chase, The involvement of iron in lipid peroxidation. Importance of ferric to ferrous ratios in initiation. J. Biol. Chem. 261, 10282–10289 (1986). 3015924

[6] S. J. Dixon, K. M. Lemberg, M. R. Lamprecht, R. Skouta, E. M. Zaitsev, C. E. Gleason, D. N. Patel, A. J. Bauer, A. M. Cantley, W. S. Yang, B. Morrison, B. R. Stockwell, Ferroptosis: An iron-dependent form of nonapoptotic cell death. Cell 149, 1060–1072 (2012). 22632970 10.1016/j.cell.2012.03.042 PMC3367386

[7] B. R. Stockwell, J. P. Friedmann Angeli, H. Bayir, A. I. Bush, M. Conrad, S. J. Dixon, S. Fulda, S. Gascón, S. K. Hatzios, V. E. Kagan, K. Noel, X. Jiang, A. Linkermann, M. E. Murphy, M. Overholtzer, A. Oyagi, G. C. Pagnussat, J. Park, Q. Ran, C. S. Rosenfeld, K. Salnikow, D. Tang, F. M. Torti, S. V. Torti, S. Toyokuni, K. A. Woerpel, D. D. Zhang, Ferroptosis: A regulated cell death nexus linking metabolism, redox biology, and disease. Cell 171, 273–285 (2017). 28985560 10.1016/j.cell.2017.09.021 PMC5685180

[8] A. Ashraf, J. Jeandriens, H. G. Parkes, P.-W. So, Iron dyshomeostasis, lipid peroxidation and perturbed expression of cystine/glutamate antiporter in Alzheimer’s disease: Evidence of ferroptosis. Redox Biol. 32, 101494 (2020). 32199332 10.1016/j.redox.2020.101494 PMC7083890

[9] N. Yan, J. Zhang, Iron metabolism, ferroptosis, and the links with Alzheimer’s disease. Front. Neurosci. 13, 1443 (2020). 32063824 10.3389/fnins.2019.01443 PMC7000453

[10] Z. Shen, J. Song, B. C. Yung, Z. Zhou, A. Wu, X. Chen, Emerging strategies of cancer therapy based on ferroptosis. Adv. Mater. 30, e1704007 (2018). 29356212 10.1002/adma.201704007 PMC6377162

[11] T. Xu, W. Ding, X. Ji, X. Ao, Y. Liu, W. Yu, J. Wang, Molecular mechanisms of ferroptosis and its role in cancer therapy. J. Cell. Mol. Med. 23, 4900–4912 (2019). 31232522 10.1111/jcmm.14511 PMC6653007

[12] A. Spolaor, P. Vallelonga, J. Gabrieli, G. Cozzi, C. Boutron, C. Barbante, Determination of Fe^2+^ and Fe^3+^ species by FIA-CRC-ICP-MS in Antarctic ice samples. J. Anal. At. Spectrom. 27, 310–317 (2012).

[13] Q. Hu, Simultaneous separation and quantification of iron and transition species using LC-ICP-MS. Am. J. Anal. Chem. 2, 675–682 (2011).

[14] N. Mironova-Ulmane, M. Polakov, A. Pavlenko, T. Zvagule, M. Eglite, E. Churbakova, N. Kurjane, T. Kärner, "The Optical And Epr Spectra of Fe2+ and Fe3+ Ions in the Blood of the Chernobyl Clean-Up Worker" in *World Congress on Medical Physics and Biomedical Engineering 2006*, R. Magjarevic, J. H. Nagel, Eds. (Springer, 2007), *IFMBE Proceedings*, pp. 2096–2098.

[15] S. Sasaki, Fe^2+^ and Fe^3+^ ions distinguishable by x-ray anomalous scattering: Method and its application to magnetite. Rev. Sci. Instrum. 66, 1573 (1995).

[16] R. J. Ogg, J. W. Langston, E. M. Haacke, R. G. Steen, J. S. Taylor, The correlation between phase shifts in gradient-echo MR images and regional brain iron concentration. Magn. Reson. Imaging 17, 1141–1148 (1999). 10499676 10.1016/s0730-725x(99)00017-x

[17] O. Dietrich, J. Levin, S.-A. Ahmadi, A. Plate, M. F. Reiser, K. Bötzel, A. Giese, B. Ertl-Wagner, MR imaging differentiation of Fe^2+^ and Fe^3+^ based on relaxation and magnetic susceptibility properties. Neuroradiology 59, 403–409 (2017). 28324122 10.1007/s00234-017-1813-3

[18] L. H. P. Vroegindeweij, L. Bossoni, A. J. W. Boon, J. H. P. Wilson, M. Bulk, J. Labra-Muñoz, M. Huber, A. Webb, L. van der Weerd, J. G. Langendonk, Quantification of different iron forms in the aceruloplasminemia brain to explore iron-related neurodegeneration. NeuroImage Clin. 30, 102657 (2021). 33839643 10.1016/j.nicl.2021.102657 PMC8055714

[19] W. Breuer, S. Epsztejn, Z. I. Cabantchik, Iron acquired from transferrin by K562 cells is delivered into a cytoplasmic pool of chelatable iron(II). J. Biol. Chem. 270, 24209–24215 (1995). 7592626 10.1074/jbc.270.41.24209

[20] O. Kakhlon, Z. I. Cabantchik, The labile iron pool: Characterization, measurement, and participation in cellular processes1 1This article is part of a series of reviews on “Iron and Cellular Redox Status.” The full list of papers may be found on the homepage of the journal. Free Radic. Biol. Med. 33, 1037–1046 (2002). 12374615 10.1016/s0891-5849(02)01006-7

[21] M. Kruszewski, Labile iron pool: The main determinant of cellular response to oxidative stress. Mutat. Res. 531, 81–92 (2003). 14637247 10.1016/j.mrfmmm.2003.08.004

[22] M. A. Smith, P. L. R. Harris, L. M. Sayre, G. Perry, Iron accumulation in Alzheimer disease is a source of redox-generated free radicals. Proc. Natl. Acad. Sci. U.S.A. 94, 9866–9868 (1997). 9275217 10.1073/pnas.94.18.9866 PMC23283

[23] H. Y. Au-Yeung, J. Chan, T. Chantarojsiri, C. J. Chang, Molecular imaging of labile iron(II) pools in living cells with a turn-on fluorescent probe. J. Am. Chem. Soc. 135, 15165–15173 (2013). 24063668 10.1021/ja4072964 PMC3838104

[24] A. T. Aron, M. O. Loehr, J. Bogena, C. J. Chang, An endoperoxide reactivity-based FRET probe for ratiometric fluorescence imaging of labile iron pools in living cells. J. Am. Chem. Soc. 138, 14338–14346 (2016). 27768321 10.1021/jacs.6b08016 PMC5749882

[25] T. Hirayama, H. Tsuboi, M. Niwa, A. Miki, S. Kadota, Y. Ikeshita, K. Okuda, H. Nagasawa, A universal fluorogenic switch for Fe(II) ion based on N-oxide chemistry permits the visualization of intracellular redox equilibrium shift towards labile iron in hypoxic tumor cells. Chem. Sci. 8, 4858–4866 (2017). 28959409 10.1039/c6sc05457a PMC5603896

[26] T. Hirayama, M. Niwa, S. Hirosawa, H. Nagasawa, High-throughput screening for the discovery of iron homeostasis modulators using an extremely sensitive fluorescent probe. ACS Sens. 5, 2950–2958 (2020). 32885952 10.1021/acssensors.0c01445

[27] A. T. Aron, M. C. Heffern, Z. R. Lonergan, M. N. Vander Wal, B. R. Blank, B. Spangler, Y. Zhang, H. M. Park, A. Stahl, A. R. Renslo, E. P. Skaar, C. J. Chang, In vivo bioluminescence imaging of labile iron accumulation in a murine model of *Acinetobacter baumannii* infection. Proc. Natl. Acad. Sci. U.S.A. 114, 12669–12674 (2017). 29138321 10.1073/pnas.1708747114 PMC5715752

[28] R. K. Muir, N. Zhao, J. Wei, Y.-H. Wang, A. Moroz, Y. Huang, Y.-C. Chen, R. Sriram, J. Kurhanewicz, D. Ruggero, A. R. Renslo, M. J. Evans, Measuring dynamic changes in the labile iron pool in vivo with a reactivity-based probe for positron emission tomography. ACS Cent. Sci. 5, 727–736 (2019). 31041393 10.1021/acscentsci.9b00240 PMC6487455

[29] T. Hirayama, A. Miki, H. Nagasawa, Organelle-specific analysis of labile Fe(II) during ferroptosis by using a cocktail of various colour organelle-targeted fluorescent probes. Metallomics 11, 111–117 (2019). 30215439 10.1039/c8mt00212f

[30] R. R. Breaker, G. F. Joyce, A DNA enzyme that cleaves RNA. Chem.Biol. 1, 223–229 (1994). 9383394 10.1016/1074-5521(94)90014-0

[31] Y. Li, R. R. Breaker, Deoxyribozymes: New players in the ancient game of biocatalysis. Curr. Opin. Struct. Biol. 9, 315–323 (1999). 10361095 10.1016/S0959-440X(99)80042-6

[32] J. Li, W. Zheng, A. H. Kwon, Y. Lu, In vitro selection and characterization of a highly efficient Zn(II)-dependent RNA-cleaving deoxyribozyme. Nucleic Acids Res. 28, 481–488 (2000). 10606646 10.1093/nar/28.2.481 PMC102519

[33] Y. Lu, New transition metal-dependent DNAzymes as efficient endonucleases and as selective metal biosensors. Chemistry 8, 4588–4596 (2002). 12362396 10.1002/1521-3765(20021018)8:20<4588::AID-CHEM4588>3.0.CO;2-Q

[34] M. Cepeda-Plaza, E. L. Null, Y. Lu, Metal ion as both a cofactor and a probe of metal-binding sites in a uranyl-specific DNAzyme: A uranyl photocleavage study. Nucleic Acids Res. 41, 9361–9370 (2013). 23939617 10.1093/nar/gkt694 PMC3814387

[35] K. Hwang, P. Hosseinzadeh, Y. Lu, Biochemical and biophysical understanding of metal ion selectivity of DNAzymes. Inorganica Chim. Acta. 452, 12–24 (2016). 27695134 10.1016/j.ica.2016.04.017 PMC5042331

[36] R. J. Lake, Z. Yang, J. Zhang, Y. Lu, DNAzymes as activity-based sensors for metal ions: Recent applications, demonstrated advantages, current challenges, and future directions. Acc. Chem. Res. 52, 3275–3286 (2019). 31721559 10.1021/acs.accounts.9b00419 PMC7103667

[37] H. E. Ihms, Y. Lu, "In Vitro Selection of Metal Ion-Selective DNAzymes" in *Ribozymes. Methods in Molecular Biology*, J. S. Hartig, Ed. (Humana Press, 2012), vol. 848, pp. 297–316. 10.1007/978-1-61779-545-9_18 PMC4162312 22315076

[38] J. Li, Y. Lu, A highly sensitive and selective catalytic DNA biosensor for lead ions. J. Am. Chem. Soc. 122, 10466–10467 (2000).

[39] J. Liu, Y. Lu, Improving fluorescent DNAzyme biosensors by combining inter- and intramolecular quenchers. Anal. Chem. 75, 6666–6672 (2003). 14640743 10.1021/ac034924r

[40] J. Liu, A. K. Brown, X. Meng, D. M. Cropek, J. D. Istok, D. B. Watson, Y. Lu, A catalytic beacon sensor for uranium with parts-per-trillion sensitivity and millionfold selectivity. Proc. Natl. Acad. Sci. U.S.A. 104, 2056–2061 (2007). 17284609 10.1073/pnas.0607875104 PMC1892917

[41] J. Liu, Y. Lu, A DNAzyme catalytic beacon sensor for paramagnetic Cu^2+^ ions in aqueous solution with high sensitivity and selectivity. J. Am. Chem. Soc. 129, 9838–9839 (2007). 17645334 10.1021/ja0717358

[42] J. Liu, Y. Lu, Rational design of “turn-on” allosteric DNAzyme catalytic beacons for aqueous mercury ions with ultrahigh sensitivity and selectivity. Angew. Chem. Int. Ed. Engl. 46, 7587–7590 (2007). 17722216 10.1002/anie.200702006

[43] T. Lan, K. Furuya, Y. Lu, A highly selective lead sensor based on a classic lead DNAzyme. Chem. Commun. (Camb) 46, 3896–3898 (2010). 20407665 10.1039/b926910j PMC3071848

[44] C. E. McGhee, K. Y. Loh, Y. Lu, DNAzyme sensors for detection of metal ions in the environment and imaging them in living cells. Curr. Opin. Biotechnol. 45, 191–201 (2017). 28458112 10.1016/j.copbio.2017.03.002 PMC5503749

[45] Z. Yang, K. Y. Loh, Y.-T. Chu, R. Feng, N. S. R. Satyavolu, M. Xiong, S. M. Nakamata Huynh, K. Hwang, L. Li, H. Xing, X. Zhang, Y. R. Chemla, M. Gruebele, Y. Lu, Optical control of metal ion probes in cells and zebrafish using highly selective DNAzymes conjugated to upconversion nanoparticles. J. Am. Chem. Soc. 140, 17656–17665 (2018). 30427666 10.1021/jacs.8b09867 PMC6473182

[46] Y. Lin, Z. Yang, R. J. Lake, C. Zheng, Y. Lu, Enzyme-mediated endogenous and bioorthogonal control of a DNAzyme fluorescent sensor for imaging metal ions in living cells. Angew. Chem. Int. Ed. Engl. 58, 17061–17067 (2019). 31529664 10.1002/anie.201910343 PMC7174831

[47] E. M. McConnell, I. Cozma, Q. Mou, J. D. Brennan, Y. Lu, Y. Li, Biosensing with DNAzymes. Chem. Soc. Rev. 50, 8954–8994 (2021). 34227631 10.1039/d1cs00240f PMC9136875

[48] S. Xing, Y. Lin, L. Cai, P. N. Basa, A. K. Shigemoto, C. Zheng, F. Zhang, S. C. Burdette, Y. Lu, Detection and quantification of tightly bound Zn^2+^ in blood serum using a photocaged chelator and a DNAzyme fluorescent sensor. Anal. Chem. 93, 5856–5861 (2021). 33787228 10.1021/acs.analchem.1c00140 PMC9169884

[49] W. Zhou, M. Vazin, T. Yu, J. Ding, J. Liu, In vitro selection of chromium-dependent DNAzymes for sensing chromium(III) and chromium(VI). Chemistry 22, 9835–9840 (2016). 27249536 10.1002/chem.201601426

[50] W. Zhou, R. Saran, P.-J. J. Huang, J. Ding, J. Liu, An exceptionally selective DNA cooperatively binding two Ca^2+^ ions. Chembiochem. 18, 518–522 (2017). 28087991 10.1002/cbic.201600708

[51] P.-J. J. Huang, J. Liu, Rational evolution of Cd^^2+^^-specific DNAzymes with phosphorothioate modified cleavage junction and Cd^2+^ sensing. Nucleic Acids Res. 43, 6125–6133 (2015). 25990730 10.1093/nar/gkv519 PMC4499143

[52] P. Bruesehoff, J. Li, A. J. Augustine 3rd, Y. Lu, Improving metal ion specificity during in vitro selection of catalytic DNA. Comb. Chem. High Throughput Screen. 5, 327–335 (2002). 12052183 10.2174/1386207023330264

[53] N. Carmi, L. A. Shultz, R. R. Breaker, In vitro selection of self-cleaving DNAs. Chem. Biol. 3, 1039–1046 (1996). 9000012 10.1016/s1074-5521(96)90170-2

[54] S. W. Santoro, G. F. Joyce, A general purpose RNA-cleaving DNA enzyme. Proc. Natl. Acad. Sci. U.S.A. 94, 4262–4266 (1997). 9113977 10.1073/pnas.94.9.4262 PMC20710

[55] S. W. Santoro, G. F. Joyce, K. Sakthivel, S. Gramatikova, C. F. Barbas 3rd, RNA cleavage by a DNA enzyme with extended chemical functionality. J. Am. Chem. Soc. 122, 2433–2439 (2000). 11543272 10.1021/ja993688s

[56] K. E. Nelson, P. J. Bruesehoff, Y. Lu, In vitro selection of high temperature Zn_2_^+^-dependent DNAzymes. J. Mol. Evol. 61, 216–225 (2005). 16096680 10.1007/s00239-004-0374-3

[57] R. Saran, J. Liu, A silver DNAzyme. Anal. Chem. 88, 4014–4020 (2016). 26977895 10.1021/acs.analchem.6b00327

[58] C. E. McGhee, Z. Yang, W. Guo, Y. Wu, M. Lyu, C. J. DeLong, S. Hong, Y. Ma, M. G. McInnis, K. S. O’Shea, Y. Lu, DNAzyme-based lithium-selective imaging reveals higher lithium accumulation in bipolar disorder patient-derived neurons. ACS Cent. Sci. 7, 1809–1820 (2021). 34841055 10.1021/acscentsci.1c00843 PMC8614110

[59] S.-F. Torabi, P. Wu, C. E. McGhee, L. Chen, K. Hwang, N. Zheng, J. Cheng, Y. Lu, In vitro selection of a sodium-specific DNAzyme and its application in intracellular sensing. Proc. Natl. Acad. Sci. U.S.A. 112, 5903–5908 (2015). 25918425 10.1073/pnas.1420361112 PMC4434688

[60] S.-F. Torabi, Y. Lu, Identification of the same Na^+^-specific DNAzyme motif from two in vitro selections under different conditions. J. Mol. Evol. 81, 225–234 (2015). 26577294 10.1007/s00239-015-9715-7

[61] D. J. R. Lane, A. M. Merlot, M. L.-H. Huang, D.-H. Bae, P. J. Jansson, S. Sahni, D. S. Kalinowski, D. R. Richardson, Cellular iron uptake, trafficking and metabolism: Key molecules and mechanisms and their roles in disease. Biochim. Biophys. Acta 1853, 1130–1144 (2015). 25661197 10.1016/j.bbamcr.2015.01.021

[62] D. Richardson, P. Ponka, E. Baker, The effect of the iron(III) chelator, desferrioxamine, on iron and transferrin uptake by the human malignant melanoma cell. Cancer Res. 54, 685–689 (1994). 8306330

[63] R. C. Hider, X. Kong, Iron speciation in the cytosol: An overview. Dalton Trans. 42, 3220–3229 (2013). 23232973 10.1039/c2dt32149a

[64] R. S. Ohgami, D. R. Campagna, A. McDonald, M. D. Fleming, The Steap proteins are metalloreductases. Blood 108, 1388–1394 (2006). 16609065 10.1182/blood-2006-02-003681 PMC1785011

[65] K. Pantopoulos, S. K. Porwal, A. Tartakoff, L. Devireddy, Mechanisms of mammalian iron homeostasis. Biochemistry 51, 5705–5724 (2012). 22703180 10.1021/bi300752r PMC3572738

[66] Q.-Q. Zhao, J.-L. Chen, T.-F. Lv, C.-X. He, G.-P. Tang, W.-Q. Liang, Y. Tabata, J.-Q. Gao, N/P ratio significantly influences the transfection efficiency and cytotoxicity of a polyethylenimine/chitosan/DNA complex. Biol. Pharm. Bull. 32, 706–710 (2009). 19336909 10.1248/bpb.32.706

[67] P. A. Longo, J. M. Kavran, M.-S. Kim, D. J. Leahy, Transient mammalian cell transfection with polyethylenimine (PEI). Methods Enzymol. 529, 227–240 (2013). 24011049 10.1016/B978-0-12-418687-3.00018-5 PMC4012321

[68] R. V. Benjaminsen, M. A. Mattebjerg, J. R. Henriksen, S. M. Moghimi, T. L. Andresen, The possible “proton sponge ” effect of polyethylenimine (PEI) does not include change in lysosomal pH. Mol. Ther. 21, 149–157 (2013). 23032976 10.1038/mt.2012.185 PMC3538306

[69] B. Chazotte, Labeling lysosomes in live cells with LysoTracker. Cold Spring Harb. Protoc. 2011, pdb.prot5571 (2011). 21285271 10.1101/pdb.prot5571

[70] FerroOrange Labile ferrous ion detecting probe | Goryo Chemical Inc. *FerroOrange Labile Ferr. Ion Detect. Probe Goryo Chem. Inc.*; https://goryochemical.com/en/research-reagent/4703/.

[71] H. Feng, B. R. Stockwell, Unsolved mysteries: How does lipid peroxidation cause ferroptosis? PLOS Biol. 16, e2006203 (2018). 29795546 10.1371/journal.pbio.2006203 PMC5991413

[72] W. S. Yang, B. R. Stockwell, Synthetic lethal screening identifies compounds activating iron-dependent, nonapoptotic cell death in oncogenic-RAS-harboring cancer cells. Chem. Biol. 15, 234–245 (2008). 18355723 10.1016/j.chembiol.2008.02.010 PMC2683762

[73] R. Shintoku, Y. Takigawa, K. Yamada, C. Kubota, Y. Yoshimoto, T. Takeuchi, I. Koshiishi, S. Torii, Lipoxygenase-mediated generation of lipid peroxides enhances ferroptosis induced by erastin and RSL3. Cancer Sci. 108, 2187–2194 (2017). 28837253 10.1111/cas.13380 PMC5666033

[74] X. Sui, R. Zhang, S. Liu, T. Duan, L. Zhai, M. Zhang, X. Han, Y. Xiang, X. Huang, H. Lin, T. Xie, RSL3 drives ferroptosis through GPX4 inactivation and ROS production in colorectal cancer. Front. Pharmacol. 9, 1371 (2018). 30524291 10.3389/fphar.2018.01371 PMC6262051

[75] T. Mosmann, Rapid colorimetric assay for cellular growth and survival: Application to proliferation and cytotoxicity assays. J. Immunol. Methods 65, 55–63 (1983). 6606682 10.1016/0022-1759(83)90303-4

[76] J.-L. Liu, Y.-G. Fan, Z.-S. Yang, Z.-Y. Wang, C. Guo, Iron and Alzheimer’s disease: From pathogenesis to therapeutic implications. Front. Neurosci. 12, 632 (2018). 30250423 10.3389/fnins.2018.00632 PMC6139360

[77] H. Oakley, S. L. Cole, S. Logan, E. Maus, P. Shao, J. Craft, A. Guillozet-Bongaarts, M. Ohno, J. Disterhoft, L. Van Eldik, R. Berry, R. Vassar, Intraneuronal β-amyloid aggregates, neurodegeneration, and neuron loss in transgenic mice with five familial Alzheimer’s disease mutations: potential factors in amyloid plaque formation. J. Neurosci. 26, 10129–10140 (2006). 17021169 10.1523/JNEUROSCI.1202-06.2006 PMC6674618

[78] J. H. Roh, Y. Huang, A. W. Bero, T. Kasten, F. R. Stewart, R. J. Bateman, D. M. Holtzman, Sleep-wake cycle and diurnal fluctuation of amyloid-β as biomarkers of brain amyloid pathology. Sci. Transl. Med. 4, 150ra122 (2012). 10.1126/scitranslmed.3004291 PMC3654377 22956200

[79] D. G. Smith, R. Cappai, K. J. Barnham, The redox chemistry of the Alzheimer’s disease amyloid β peptide. Biochim. Biophys. Acta 1768, 1976–1990 (2007). 17433250 10.1016/j.bbamem.2007.02.002

[80] J. Everett, E. Céspedes, L. R. Shelford, C. Exley, J. F. Collingwood, J. Dobson, G. van der Laan, C. A. Jenkins, E. Arenholz, N. D. Telling, Evidence of redox-active iron formation following aggregation of ferrihydrite and the Alzheimer’s disease peptide β-amyloid. Inorg. Chem. 53, 2803–2809 (2014). 24559299 10.1021/ic402406g

[81] J. Everett, E. Céspedes, L. R. Shelford, C. Exley, J. F. Collingwood, J. Dobson, G. van der Laan, C. A. Jenkins, E. Arenholz, N. D. Telling, Ferrous iron formation following the co-aggregation of ferric iron and the Alzheimer’s disease peptide β-amyloid (1–42). J. R. Soc. Interface 11, 20140165 (2014). 24671940 10.1098/rsif.2014.0165 PMC4006257

[82] M. Gao, P. Monian, Q. Pan, W. Zhang, J. Xiang, X. Jiang, Ferroptosis is an autophagic cell death process. Cell Res. 26, 1021–1032 (2016). 27514700 10.1038/cr.2016.95 PMC5034113

[83] G. O. Latunde-Dada, Ferroptosis: Role of lipid peroxidation, iron and ferritinophagy. Biochim. Biophys. Acta Gen. Subj. 1861, 1893–1900 (2017). 28552631 10.1016/j.bbagen.2017.05.019

[84] F. Kuang, J. Liu, D. Tang, R. Kang, Oxidative damage and antioxidant defense in ferroptosis. Front. Cell Dev. Biol. 8, 586578 (2020). 33043019 10.3389/fcell.2020.586578 PMC7527737

[85] J. M. G. van Bergen, X. Li, J. Hua, S. J. Schreiner, S. C. Steininger, F. C. Quevenco, M. Wyss, A. F. Gietl, V. Treyer, S. E. Leh, F. Buck, R. M. Nitsch, K. P. Pruessmann, P. C. M. van Zijl, C. Hock, P. G. Unschuld, Colocalization of cerebral iron with amyloid β in mild cognitive impairment. Sci. Rep. 6, 35514 (2016). 27748454 10.1038/srep35514 PMC5066274

[86] N. D. Telling, J. Everett, J. F. Collingwood, J. Dobson, G. van der Laan, J. J. Gallagher, J. Wang, A. P. Hitchcock, Iron biochemistry is correlated with amyloid plaque morphology in an established mouse model of Alzheimer’s disease. Cell Chem. Biol. 24, 1205–1215.e3 (2017). 28890316 10.1016/j.chembiol.2017.07.014

[87] J. Everett, J. Brooks, F. Lermyte, P. B. O’Connor, P. J. Sadler, J. Dobson, J. F. Collingwood, N. D. Telling, Iron stored in ferritin is chemically reduced in the presence of aggregating Aβ(1-42). Sci. Rep. 10, 10332 (2020). 32587293 10.1038/s41598-020-67117-z PMC7316746

[88] C. A. Schneider, W. S. Rasband, K. W. Eliceiri, NIH Image to ImageJ: 25 years of image analysis. Nat. Methods 9, 671–675 (2012). 22930834 10.1038/nmeth.2089 PMC5554542

[89] S. Bolte, F. P. Cordelières, A guided tour into subcellular colocalization analysis in light microscopy. J. Microsc. 224, 213–232 (2006). 17210054 10.1111/j.1365-2818.2006.01706.x

[90] N. R. Markham, M. Zuker, UNAFold: Software for nucleic acid folding and hybridization. Methods Mol. Biol. 453, 3–31 (2008). 18712296 10.1007/978-1-60327-429-6_1

[91] S. W. Taylor, D. B. Chase, M. H. Emptage, M. J. Nelson, J. H. Waite, Ferric ion complexes of a DOPA-containing adhesive protein from *Mytilus edulis*. Inorg. Chem. 35, 7572–7577 (1996).

[92] D. S. Hwang, H. Zeng, A. Masic, M. J. Harrington, J. N. Israelachvili, J. H. Waite, Protein- and metal-dependent interactions of a prominent protein in mussel adhesive plaques. J. Biol. Chem. 285, 25850–25858 (2010). 20566644 10.1074/jbc.M110.133157 PMC2919147

[93] H. Zeng, D. S. Hwang, J. N. Israelachvili, J. H. Waite, Strong reversible Fe^3+^-mediated bridging between dopa-containing protein films in water. Proc. Natl. Acad. Sci. U.S.A. 107, 12850–12853 (2010). 20615994 10.1073/pnas.1007416107 PMC2919964

