# Supplementary material for: Simultaneous Fe2+/Fe3+ imaging shows Fe3+ over Fe2+ enrichment in Alzheimer’s disease mouse brain
Source: Sci Adv. 2023 Apr 19;9(16):eade7622. doi: 10.1126/sciadv.ade7622 (PMC10115418; doi:10.1126/sciadv.ade7622)
Supplement: Supplementary file 1 — Figs. S1 to S33 Tables S1 to S7 References [file sciadv.ade7622_sm.pdf]

Science Advances
AAAS

Supplementary Materials for

Simultaneous Fe^{2+}/Fe^{3+} imaging shows Fe^{3+} over Fe^{2+} enrichment in Alzheimer's disease mouse brain

Yuting Wu *et al.*

Corresponding author: Yi Lu, yi.lu@utexas.edu

Sci. Adv. **9**, eade7622 (2023)
DOI: 10.1126/sciadv.ade7622

This PDF file includes:

Figs. S1 to S33
Tables S1 to S7
References

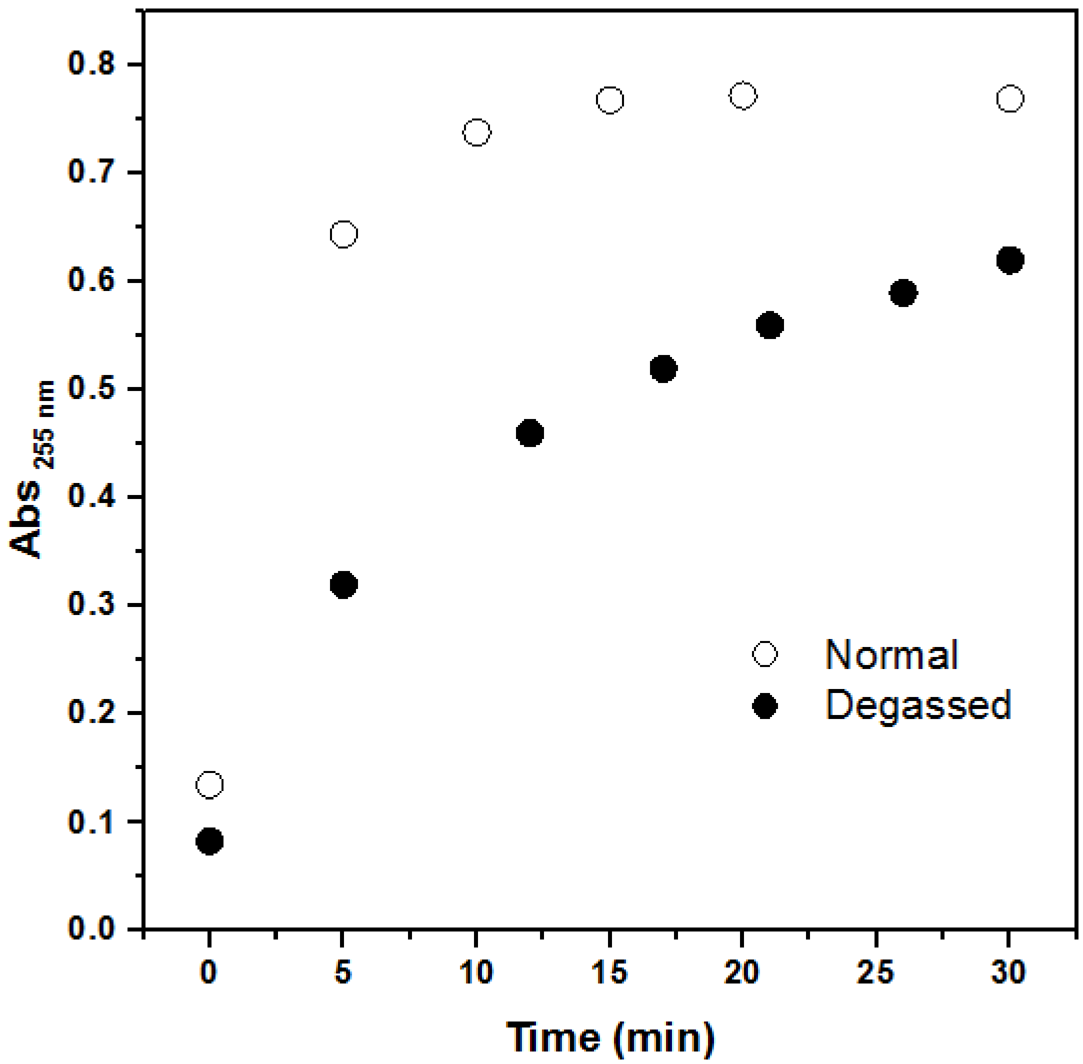

Fig. S1. Oxidation of Fe^{2+} solutions in degassed (filled circles) and air equilibrated buffer (open circles) in 10 mM Bis-Tris, pH 7.0 over 30 minutes.

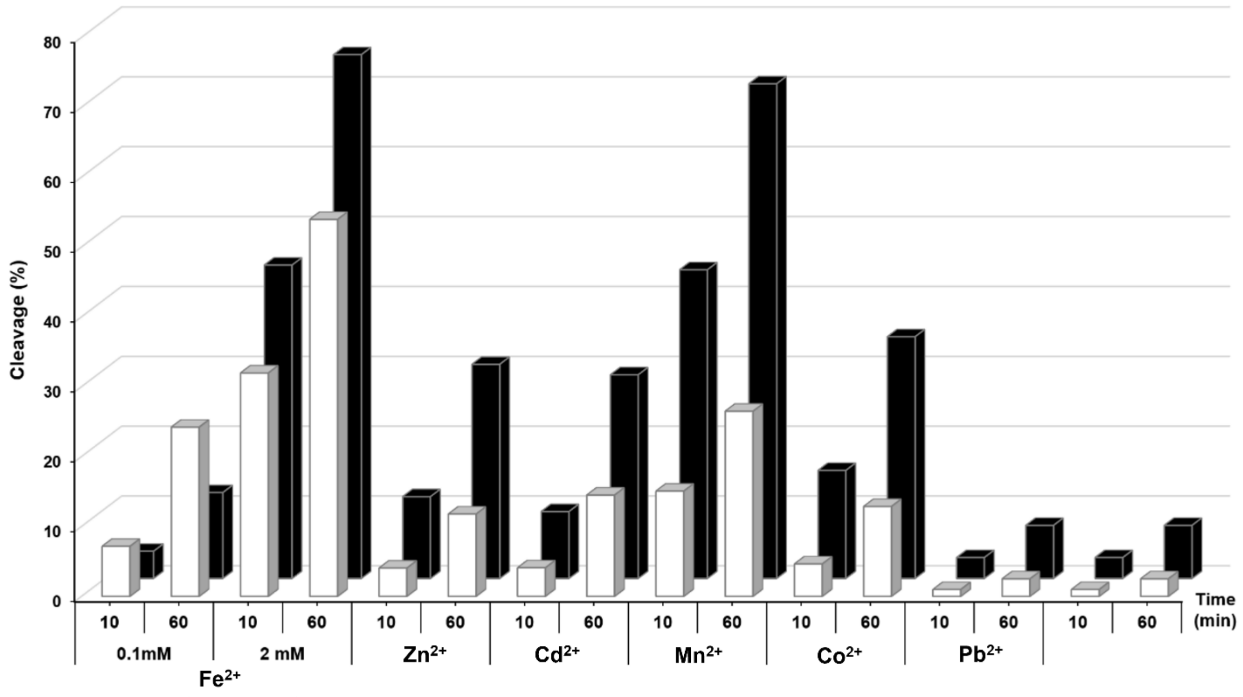

Fig. S2. Comparing the activity of selected pools in the presence of glutathione (GSH), with (white) and without (black) counter selection steps.

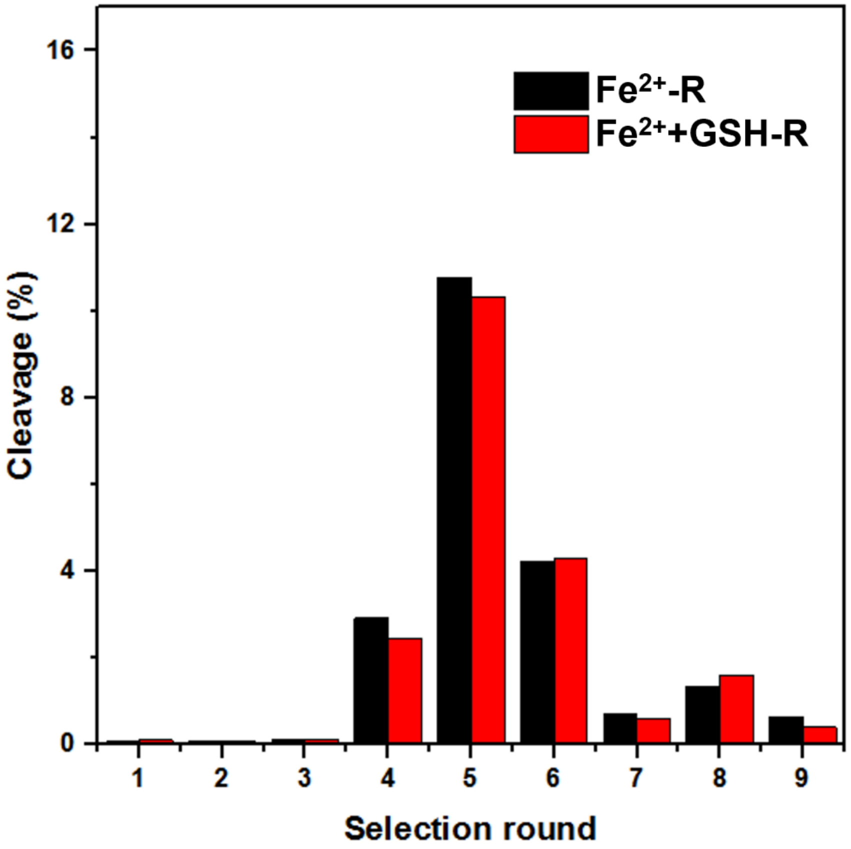

Fig. S3. In vitro selection progress in terms of cleavage products observed in different rounds. "R" in the legends represent incorporation of a counter selection before each positive selection, started from round 3.

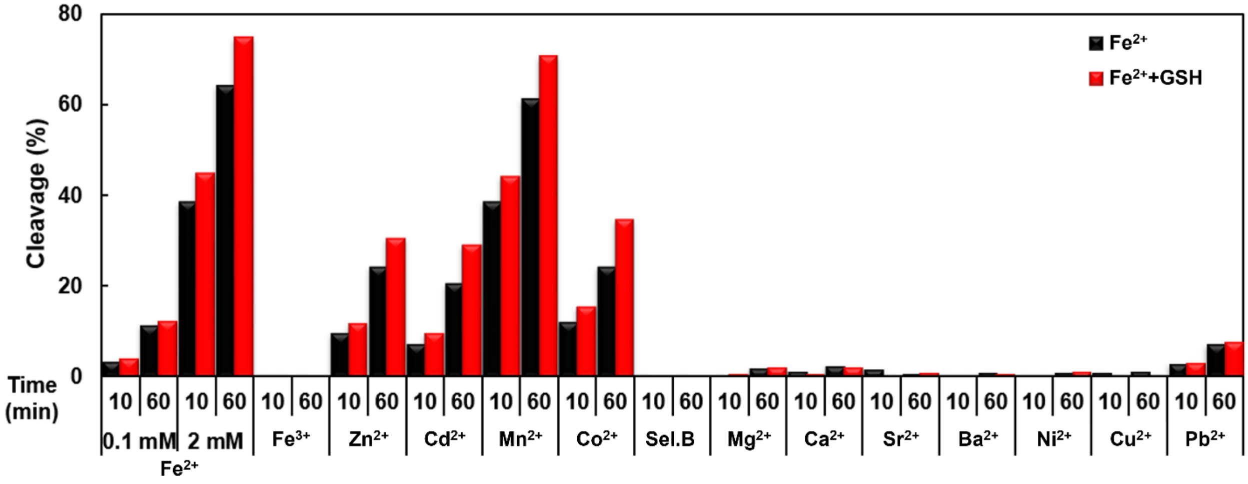

Fig. S4. Activity of selected pools from round 5. Percentages of cleavage product are provided for 10 and 60 min. Two different concentrations of Fe^{2+} were tested: 0.1 and 2 mM. Cleavage activity of the pools were tested in the presence of 0.25 mM Fe^{3+} or 0.1 mM Pb^{2+}. All other divalent metal ions were tested at 2 mM. "Sel. B." represents cleavage activity observed in selection buffer in the absence of Fe^{2+}.

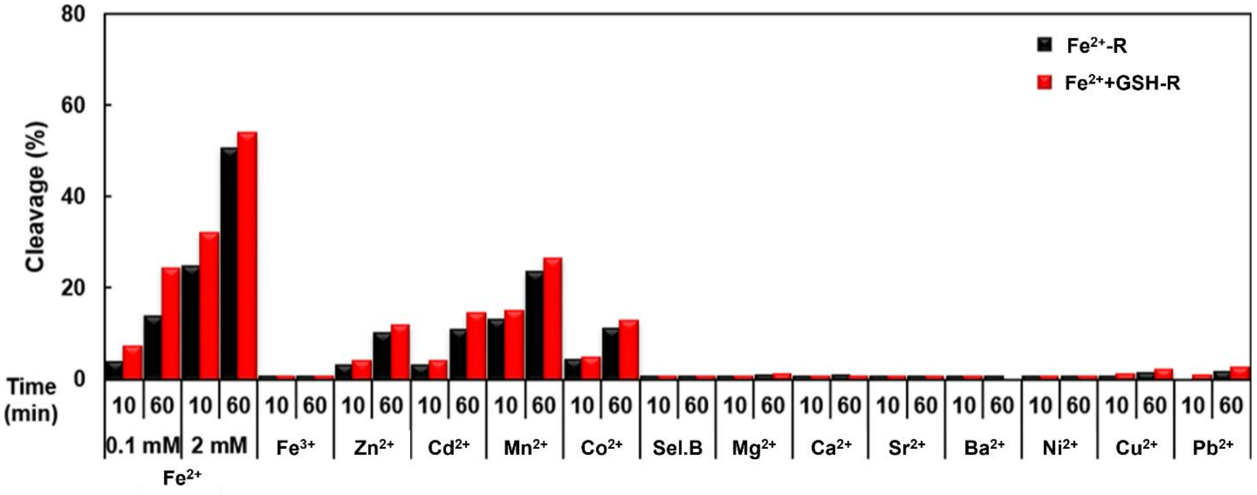

Fig. S5. Activity of selected pools from round 5 after introduction of 3 counter selection steps. Percentages of cleavage product are provided for 10 and 60 min. Two different concentrations of Fe^{2+} were tested: 0.1 and 2 mM. Cleavage activity of the pools were tested in the presence of 0.25 mM Fe^{3+} or 0.1 mM Pb^{2+}. All other divalent metal ions were tested at 2 mM. "Sel. B." represents cleavage activity observed in selection buffer in the absence of Fe^{2+}.

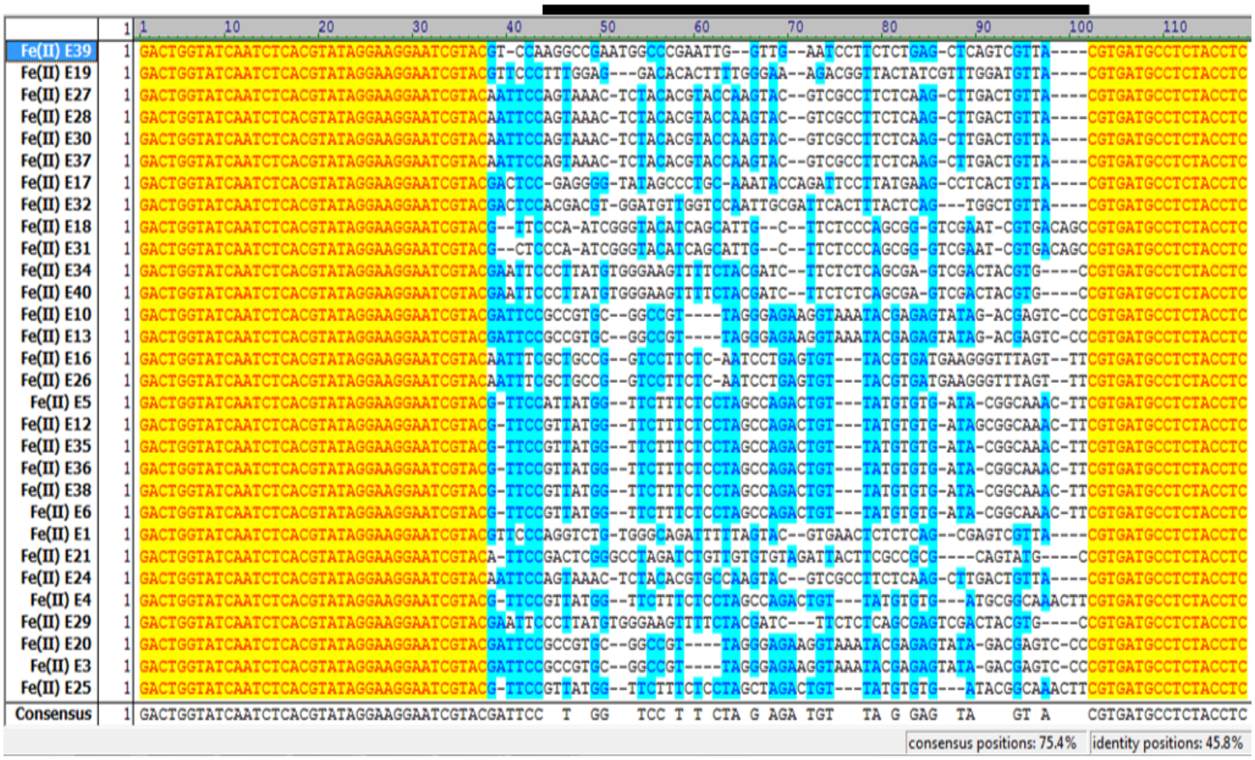

Fig. S6. Sequence alignment of 34 clones isolated from selection condition E showing the similarity and difference between sequences enriched under that selection condition. The location of the N50 random region is indicated with a black line. Red text with yellow background indicates identical regions, which mainly represent the primer binding sites. The dark blue text on a light blue background represents regions that are conserved between different isolated sequences. Conserved nucleotides might serve important roles in catalytic activity of the DNAzymes. Nucleotides showed in black on a white background define regions that are variant between different selected DNAzymes in each pool. Comprehensive biochemical characterization and mutational studies for each DNAzyme are required to reveal the significance of those regions on cleavage activity.

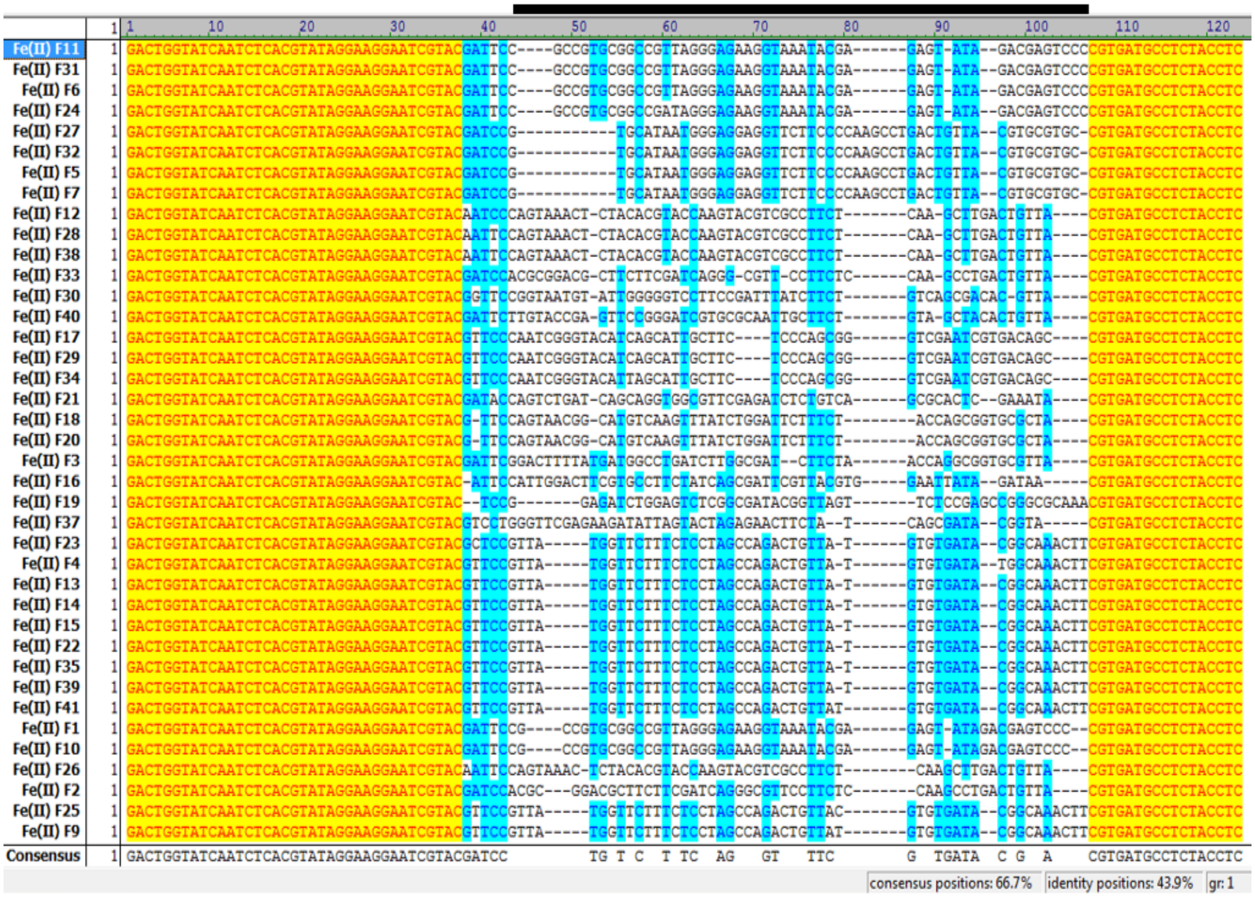

Fig. S7. Sequence alignment of 40 clones isolated from selection condition F showing the similarity and difference between sequences enriched under that selection condition. The location of the N50 random region is indicated with a black line. Red text with yellow background indicates identical regions, which mainly represent the primer binding sites. The dark blue text on a light blue background represents regions that are conserved between different isolated sequences. Conserved nucleotides might serve important roles in catalytic activity of the DNAzymes. Nucleotides showed in black on a white background define regions that are variant between different selected DNAzymes in each pool. Comprehensive biochemical characterization and mutational studies for each DNAzyme are required to reveal the significance of those regions on cleavage activity.

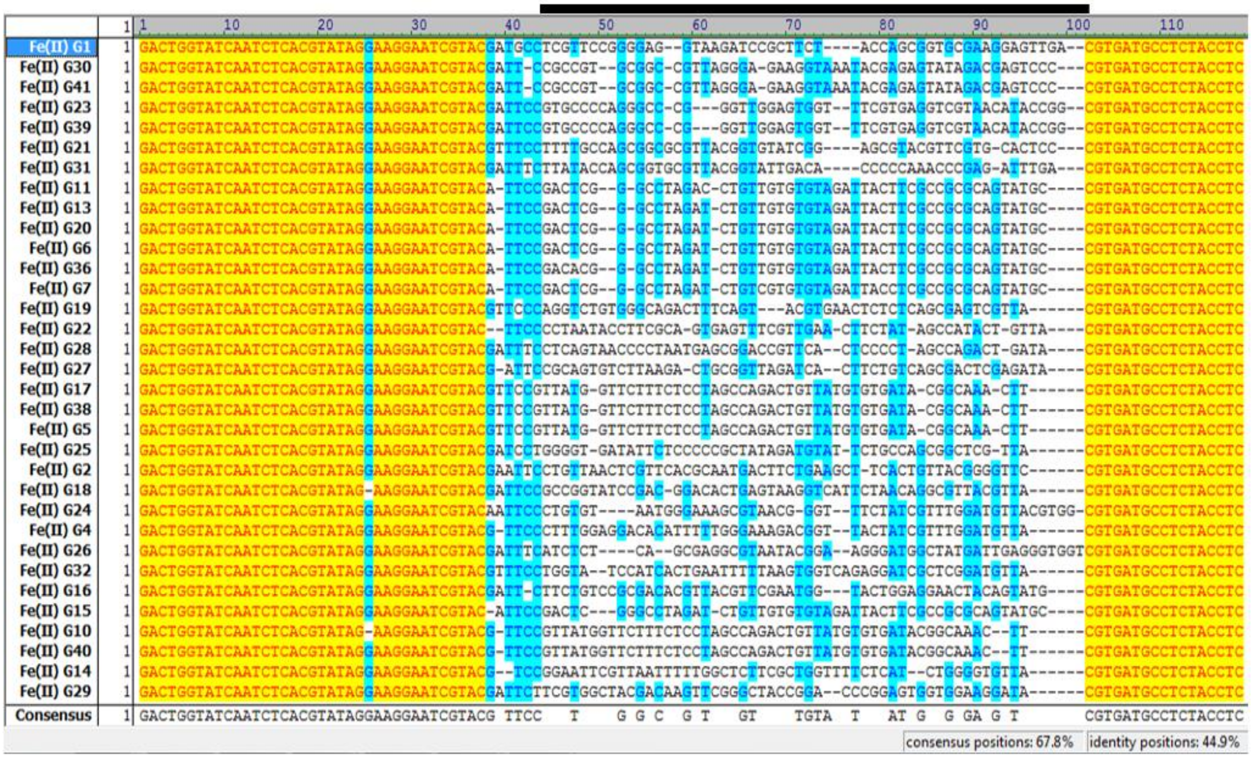

Fig. S8. Sequence alignment of 36 clones isolated from selection condition G showing the similarity and difference between sequences enriched under that selection condition. The location of the N50 random region is indicated with a black line. Red text with yellow background indicates identical regions, which mainly represent the primer binding sites. The dark blue text on a light blue background represents regions that are conserved between different isolated sequences. Conserved nucleotides might serve important roles in catalytic activity of the DNAzymes. Nucleotides showed in black on a white background define regions that are variant between different selected DNAzymes in each pool. Comprehensive biochemical characterization and mutational studies for each DNAzyme are required to reveal the significance of those regions on cleavage activity.

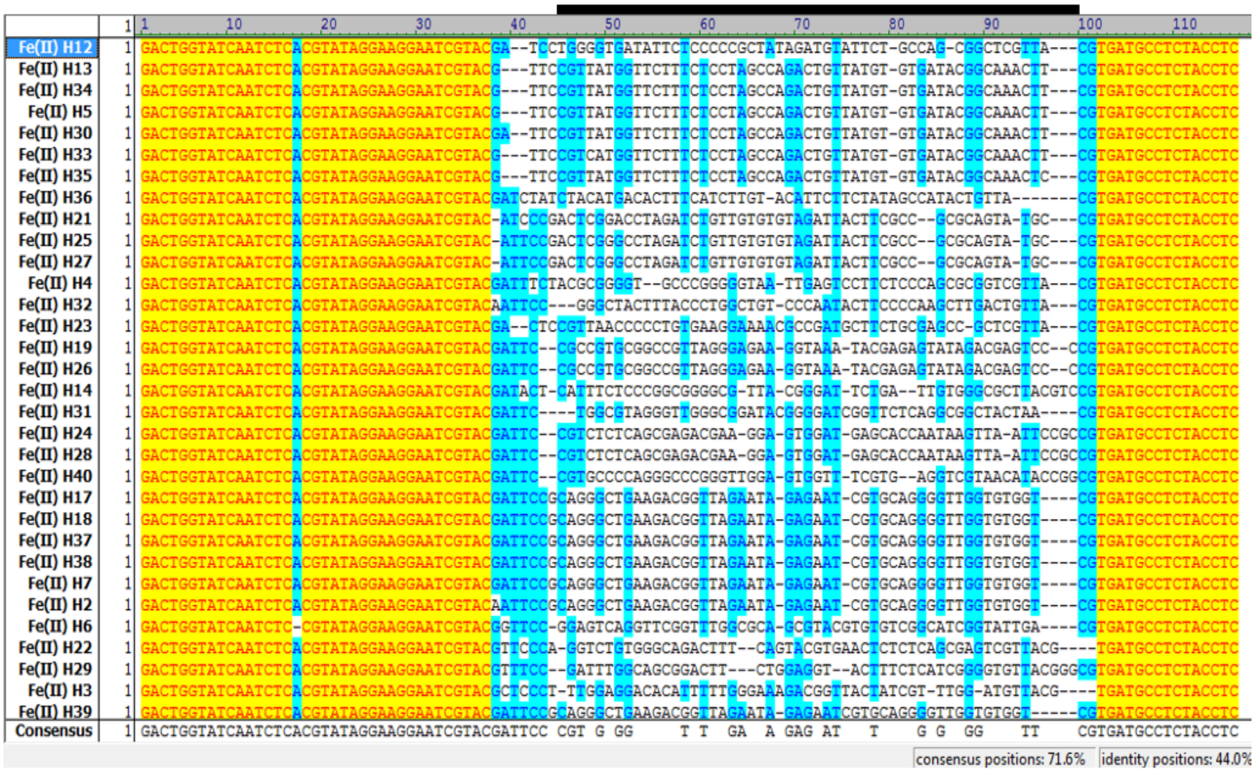

Fig. S9. Sequence alignment of 39 clones isolated from selection condition H showing the similarity and difference between sequences enriched under that selection condition. The location of the N50 random region is indicated with a black line. Red text with yellow background indicates identical regions, which mainly represent the primer binding sites. The dark blue text on a light blue background represents regions that are conserved between different isolated sequences. Conserved nucleotides might serve important roles in catalytic activity of the DNAzymes. Nucleotides showed in black on a white background define regions that are variant between different selected DNAzymes in each pool. Comprehensive biochemical characterization and mutational studies for each DNAzyme are required to reveal the significance of those regions on cleavage activity.

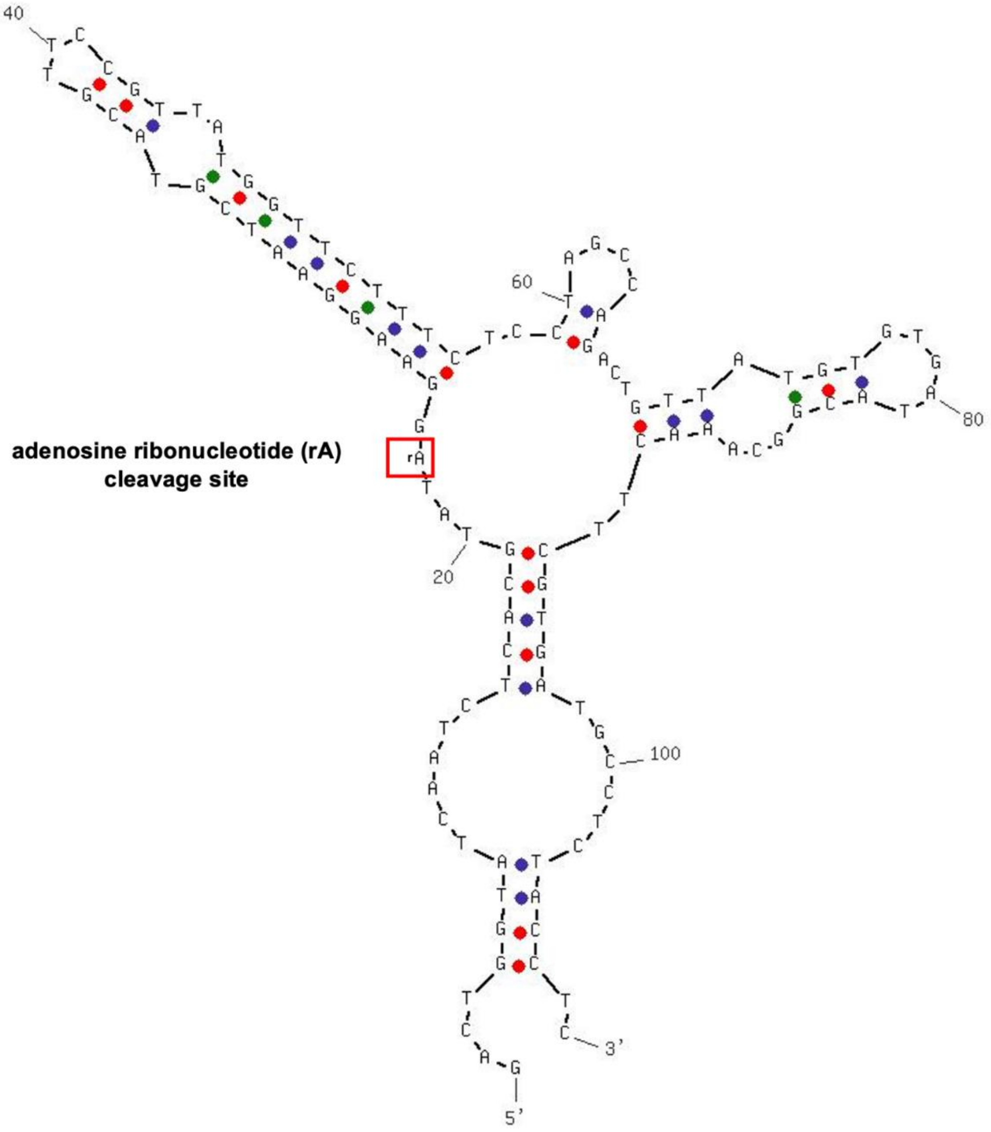

Fig. S10. Sequence and predicted secondary structure of the cis-acting Fe(II)-H5 DNAzyme. The structure prediction was performed with UNAfold (*90*) at 25 °C at 1 M Na^+.

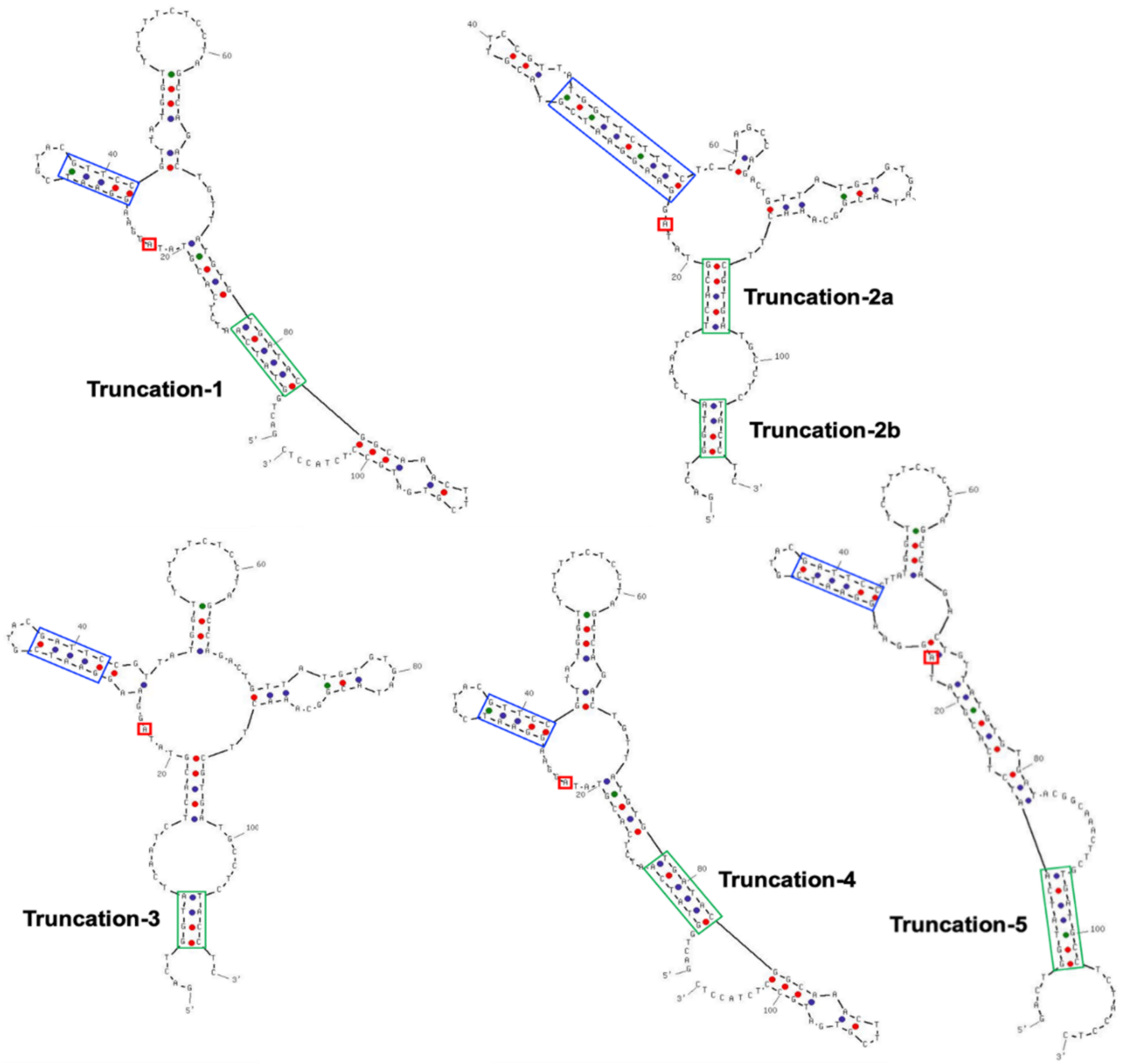

Fig. S11. Several predicted secondary structures of the cis-acting Fe(II)-H5 DNAzyme that represent different catalytic core conformations were selected for truncation studies. In each secondary structure, potential enzyme-substrate binding arms (boxed in green and blue) were identified. Regions outside the binding arms were removed by truncation. Removed regions include 1) 3'- and 5'-tails below binding arms boxed in green and 2) loop nucleotides on the left side of the binding arm boxed in blue. Binding arms in trans-acting DNAzymes were extended if their melting temperature was below 20 °C to support proper enzyme-substrate hybridization at room temperature. The intended cleavage site is a single adenosine ribonucleotide (rA), which is boxed in red. Activity of trans-acting DNAzymes were tested in the presence of Fe^{2+} to identify the minimal catalytic sequence that efficiently cleaves the substrate strand. The only catalytically active trans-acting DNAzyme was truncation-2b, which was used to design the Fe(II)-H5 fluorescent sensor (Figure 1A).

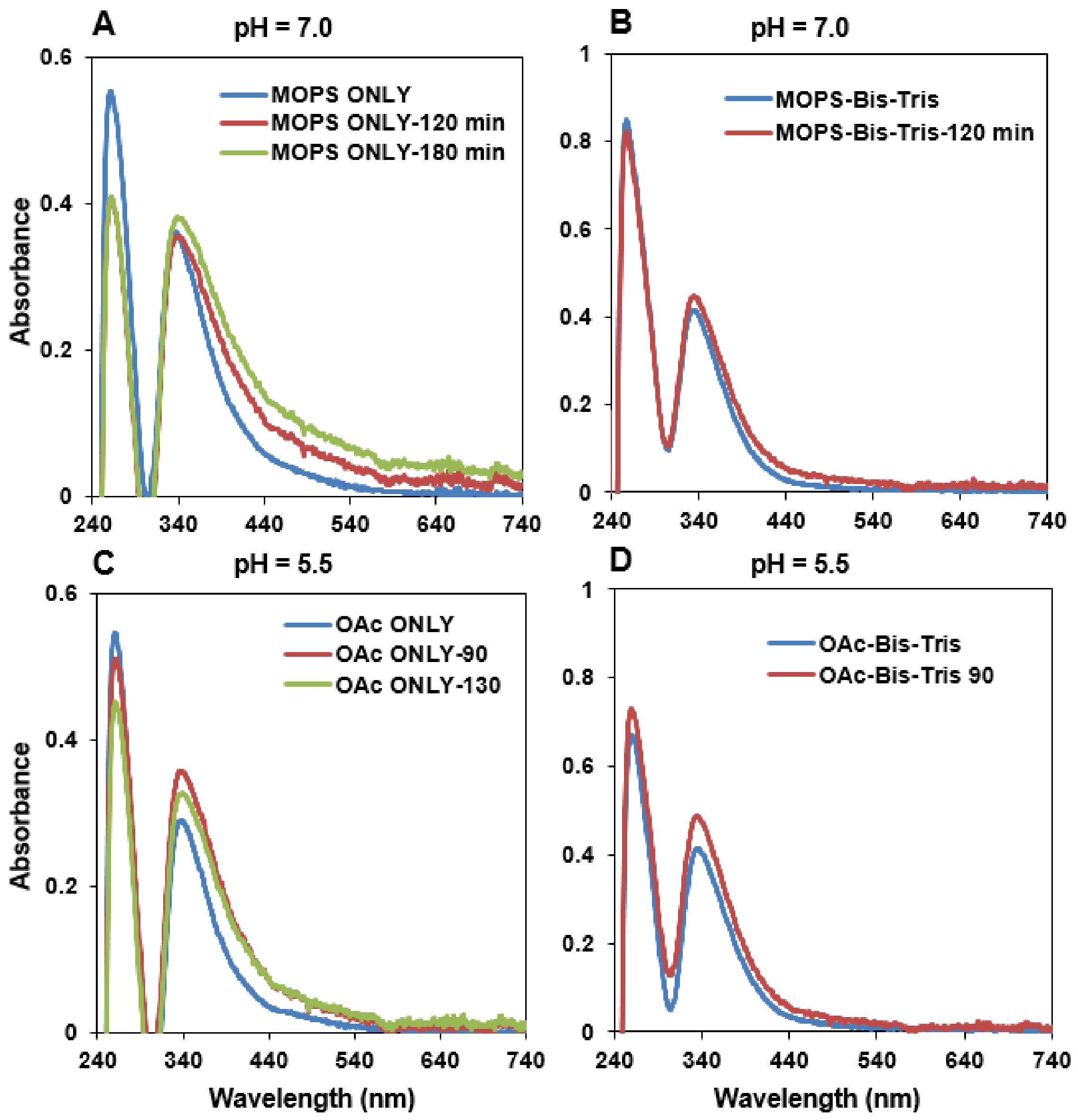

Fig. S12. Time-dependent (in minutes) UV-vis spectral changes of 0.1 mM Fe^{3+} in 20 mM MOPS at pH 7.0 (A), 20 mM MOPS + 5 mM Bis-Tris at pH 7.0 (B), 20 mM acetate at pH 5.5 (C), and 20 mM acetate + 5 mM Bis-Tris at pH 5.5 (D). A decrease of the peak at ~250 nm and an increase of the peak at ~340 nm indicates that Fe^{3+} is not stable in either MOPS at pH 7.0 or acetate at pH 5.5. Adding 5 mM Bis-Tris, a known ligand for Fe^{3+}, minimized the spectral changes at both pH conditions, indicating that inclusion of Bis-Tris improves the stability of the Fe^{3+} in solution. Our finding is consistent with earlier observation on the effect of Bis-Tris on Fe^{3+} solubilization at both pH 5.5 and 7.0 (*91–93*). Despite inclusion of highly stringent negative selections, our attempts in isolating Fe^{3+}-specific DNAzymes in pH 7.0 even in the presence of Bis-Tris failed, resulting in Fe^{3+}-independent cleavage activity. This observation suggests that, although Bis-Tris can stabilize Fe^{3+} at both pH 5.5 and 7.0, under our selection conditions, the “labile” Fe^{3+} is accessible to DNA and triggers cleavage only at pH 5.5, but not at pH 7.

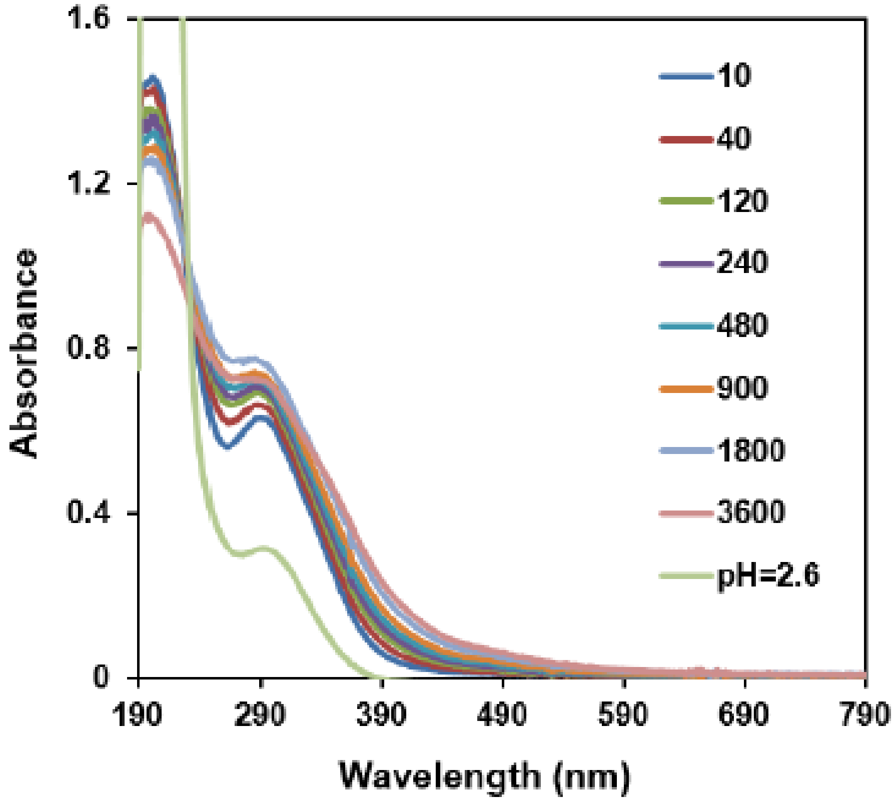

Fig. S13. UV-vis spectra of 0.2 mM Fe^{3+} in water at pH 4.0. As a control Fe^{3+} spectra at pH 2.6, in which Fe^{3+} is stable, is shown in light green. Spectra collected at different time points (seconds). Changes in the UV-vis spectra (e.g., a decrease of the peak at ~200 nm and an increase of the peak at ~290 nm) of Fe^{3+} solutions at pH 4.0 indicates that Fe^{3+} is not stable in aqueous solution in the absence of a stabilizing chelator.

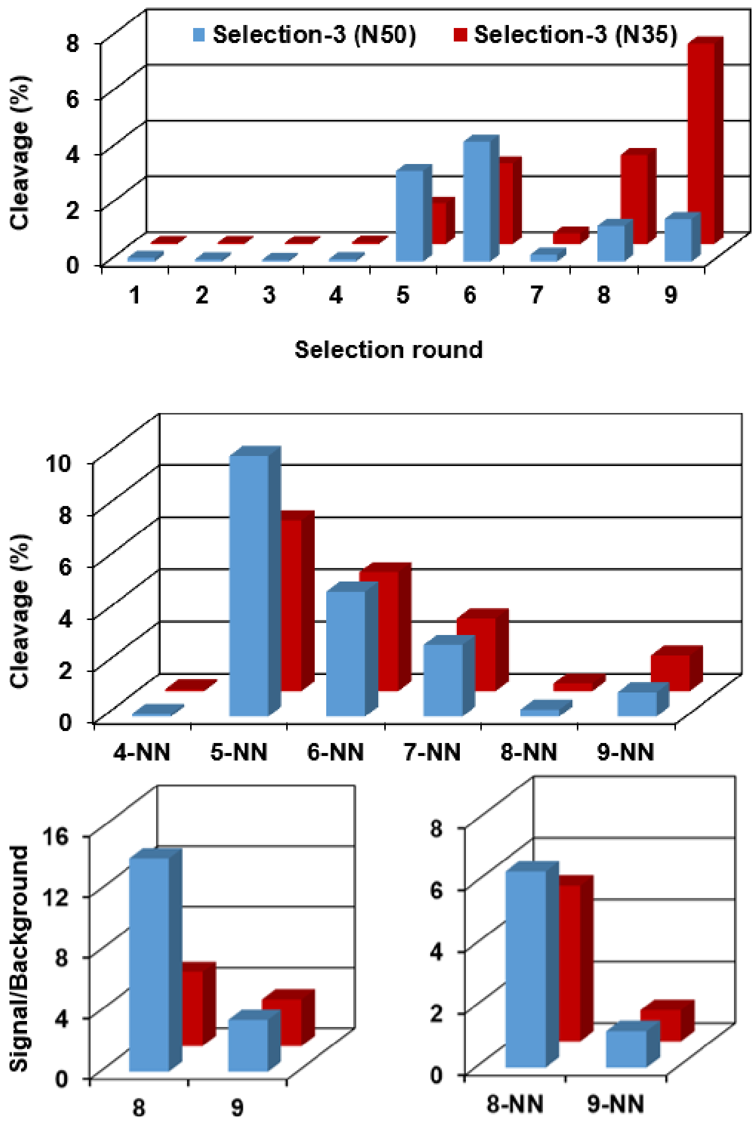

Fig. S14. Selection progress in terms of the percentage of cleavage product (cleavage %, top two figures) and the ratio of activity of the DNA pool over the nonspecific background cleavage for each round (signal/background; bottom two figures. Two different pools with different random regions, N35 and N50, are shown in blue and red, respectively. Selection rounds carried out without prior negative selection are shown with “NN”. Despite observing an increase in the cleavage activity (Cleavage (%)) of the selected pools at round 9 (see the top two figures), the ratio of the Fe^{3+}-dependent cleavage activity over nonspecific background cleavage (Signal/Background) decreases from round 8 to 9 (see the bottom two figures). These observations indicate that the higher activity observed in selection round 9 is mainly due to an increase in nonspecific (background) cleavage activity. The drop in the ratio of Fe^{3+}-specific activity over background cleavage is an indication that continuing selection process will not result in further enrichment of Fe^{3+}-specific DNAzymes.

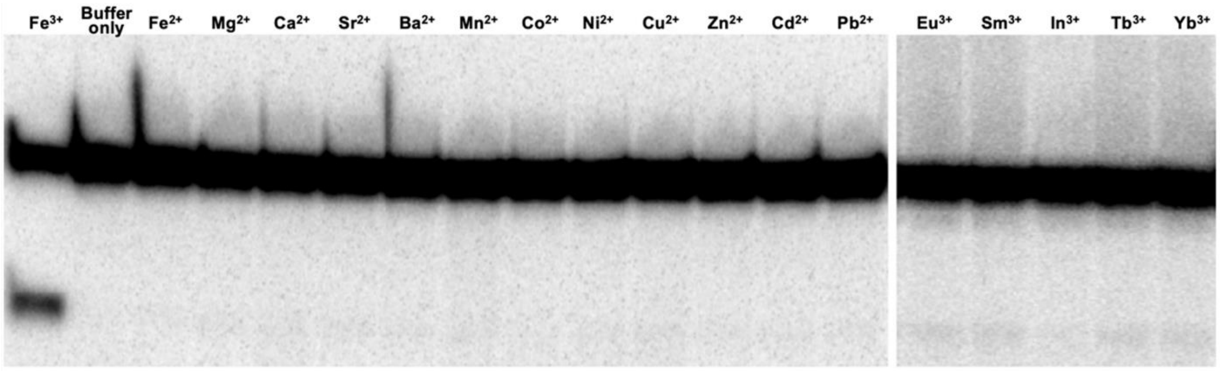

Fig. S15. Selectivity of the enriched pool under condition H is evaluated in the selection buffer in the presence of 50 μM of different metal ions. Only in the presence of Fe^{3+} formation of the cleavage product is observed (lower band on the left). The buffer-only condition (the second lane from left) represents cleavage activity in the absence of multivalent metal ions (background cleavage).

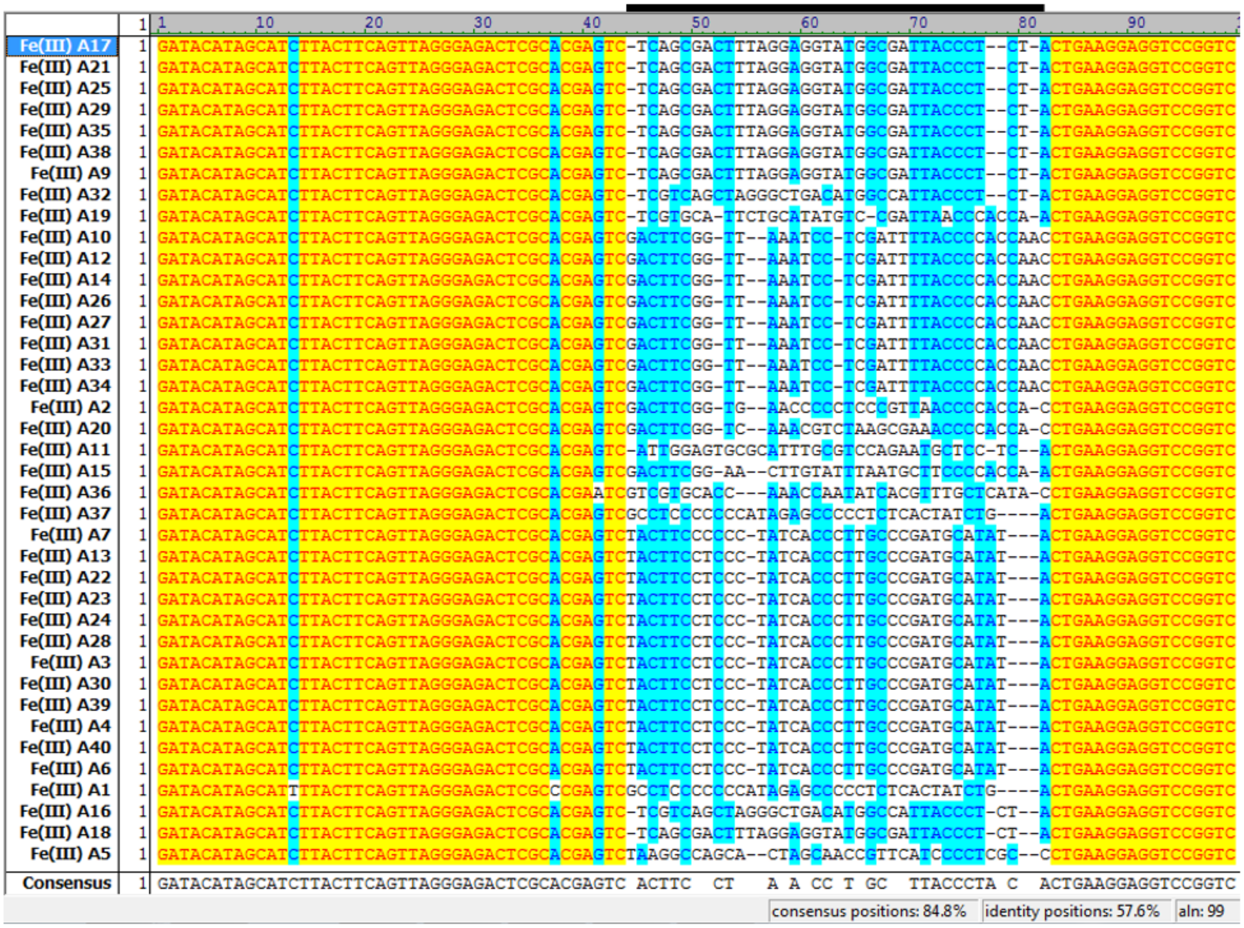

Fig. S16. Sequence alignment of 39 clones isolated from selection condition A showing the similarity and difference between sequences enriched under that selection condition. The location of the N50 random region is indicated with a black line. Red text with yellow background indicates identical regions, which mainly represent the primer binding sites. The dark blue text on a light blue background represents regions that are conserved between different isolated sequences. Conserved nucleotides might serve important roles in catalytic activity of the DNAzymes. Nucleotides showed in black on a white background define regions that are variant between different selected DNAzymes in each pool. Comprehensive biochemical characterization and mutational studies for each DNAzyme are required to reveal the significance of those regions on cleavage activity.

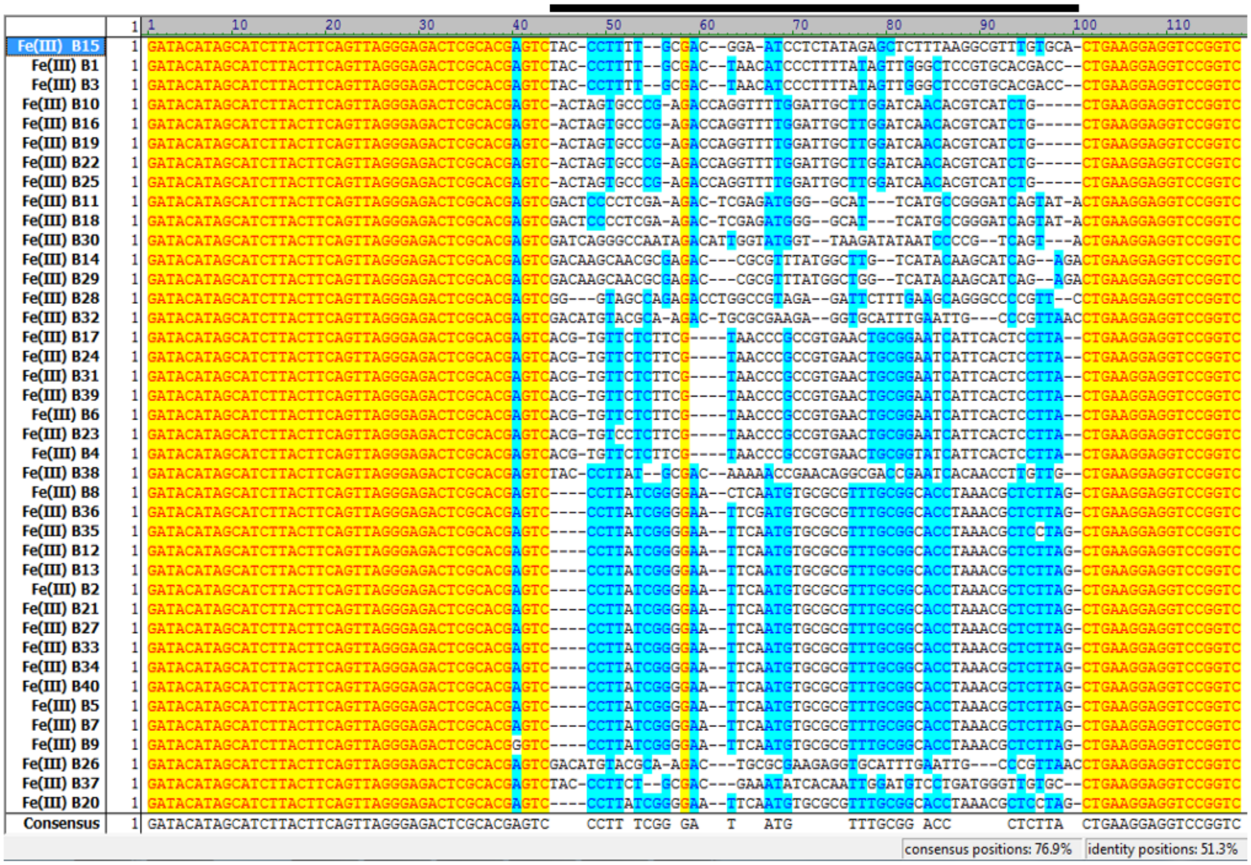

Fig. S17. Sequence alignment of 40 clones isolated from selection condition B showing the similarity and difference between sequences enriched under that selection condition. The location of the N50 random region is indicated with a black line. Red text with yellow background indicates identical regions, which mainly represent the primer binding sites. The dark blue text on a light blue background represents regions that are conserved between different isolated sequences. Conserved nucleotides might serve important roles in catalytic activity of the DNAzymes. Nucleotides showed in black on a white background define regions that are variant between different selected DNAzymes in each pool. Comprehensive biochemical characterization and mutational studies for each DNAzyme are required to reveal the significance of those regions on cleavage activity.

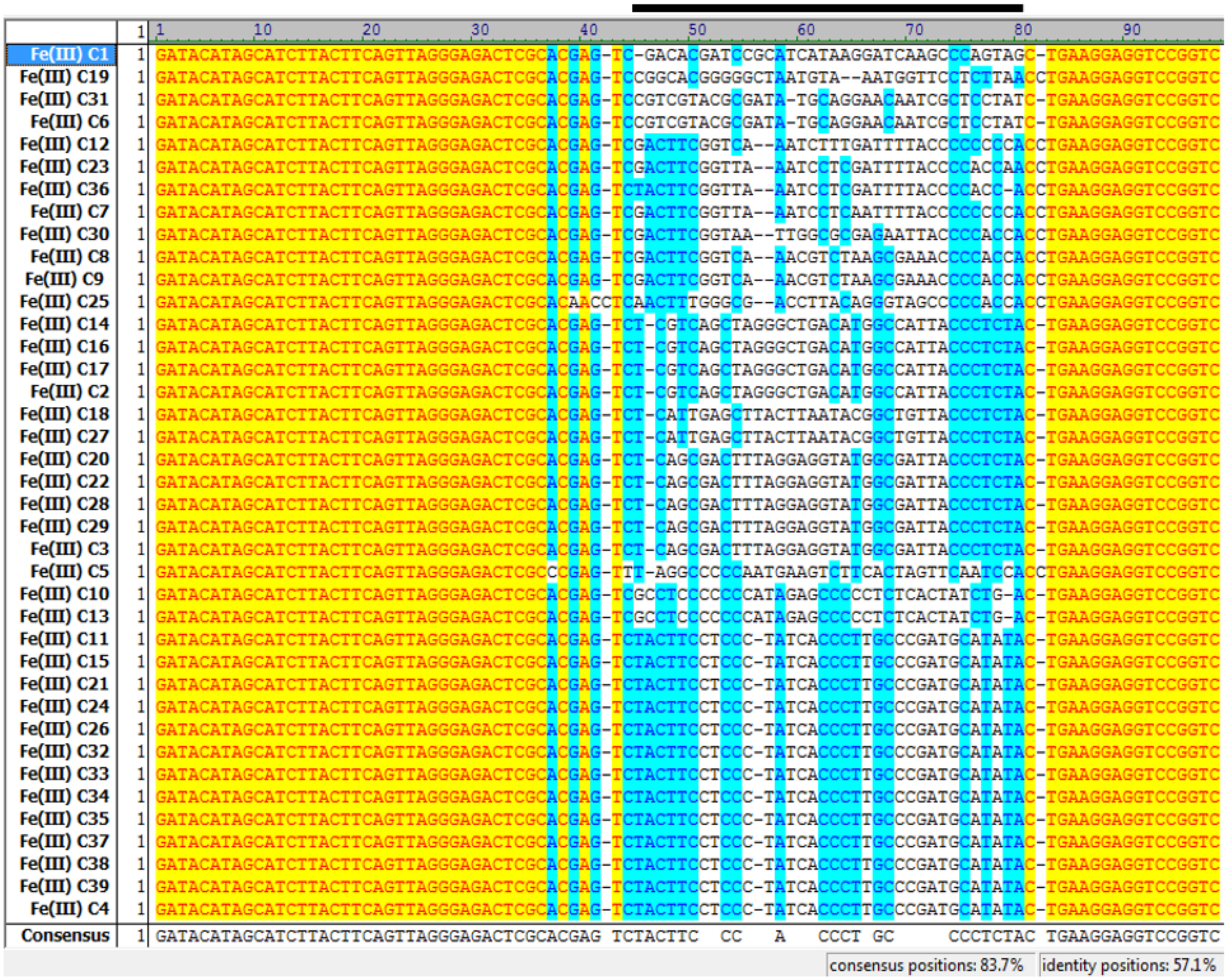

Fig. S18. Sequence alignment of 39 clones isolated from selection condition C showing the similarity and difference between sequences enriched under that selection condition. The location of the N50 random region is indicated with a black line. Red text with yellow background indicates identical regions, which mainly represent the primer binding sites. The dark blue text on a light blue background represents regions that are conserved between different isolated sequences. Conserved nucleotides might serve important roles in catalytic activity of the DNAzymes. Nucleotides showed in black on a white background define regions that are variant between different selected DNAzymes in each pool. Comprehensive biochemical characterization and mutational studies for each DNAzyme are required to reveal the significance of those regions on cleavage activity.

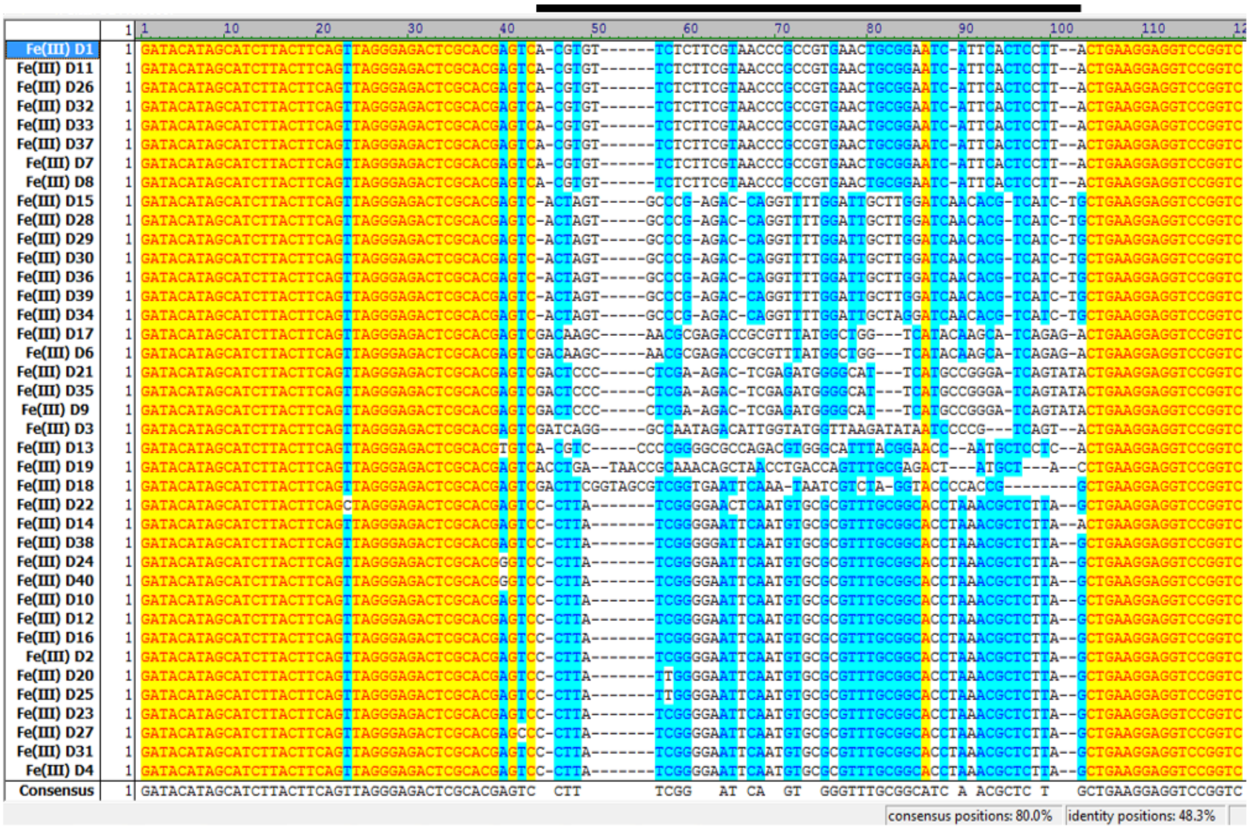

Fig. S19. Sequence alignment of 39 clones isolated from selection condition D showing the similarity and difference between sequences enriched under that selection condition. The location of the N50 random region is indicated with a black line. Red text with yellow background indicates identical regions, which mainly represent the primer binding sites. The dark blue text on a light blue background represents regions that are conserved between different isolated sequences. Conserved nucleotides might serve important roles in catalytic activity of the DNAzymes. Nucleotides showed in black on a white background define regions that are variant between different selected DNAzymes in each pool. Comprehensive biochemical characterization and mutational studies for each DNAzyme are required to reveal the significance of those regions on cleavage activity.

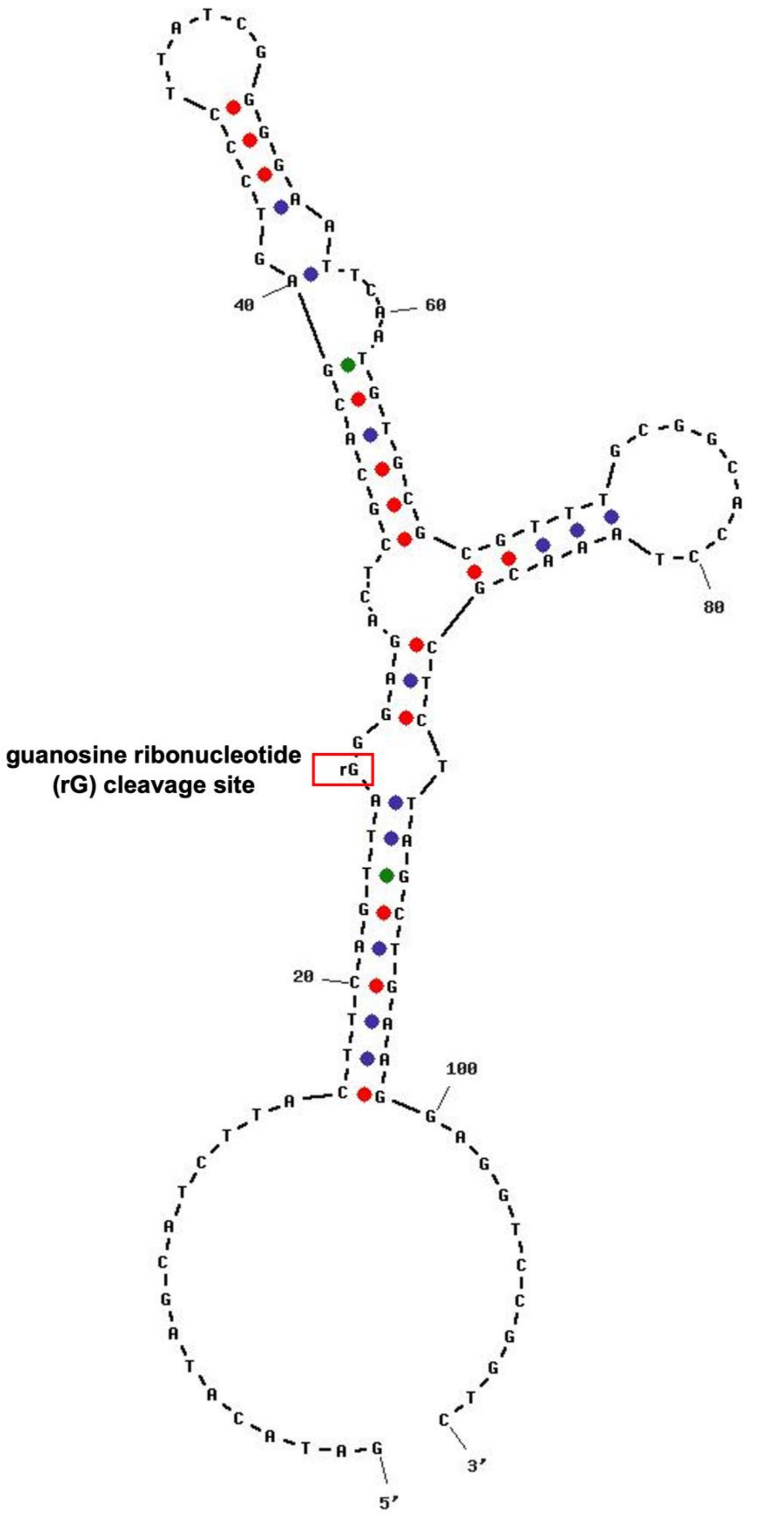

Fig. S20. Sequence and predicted secondary structure of the cis-acting Fe(III)-B12 DNAzyme. The structure prediction was performed with UNAfold (*90*) at 25 °C at 1 M Na^+.

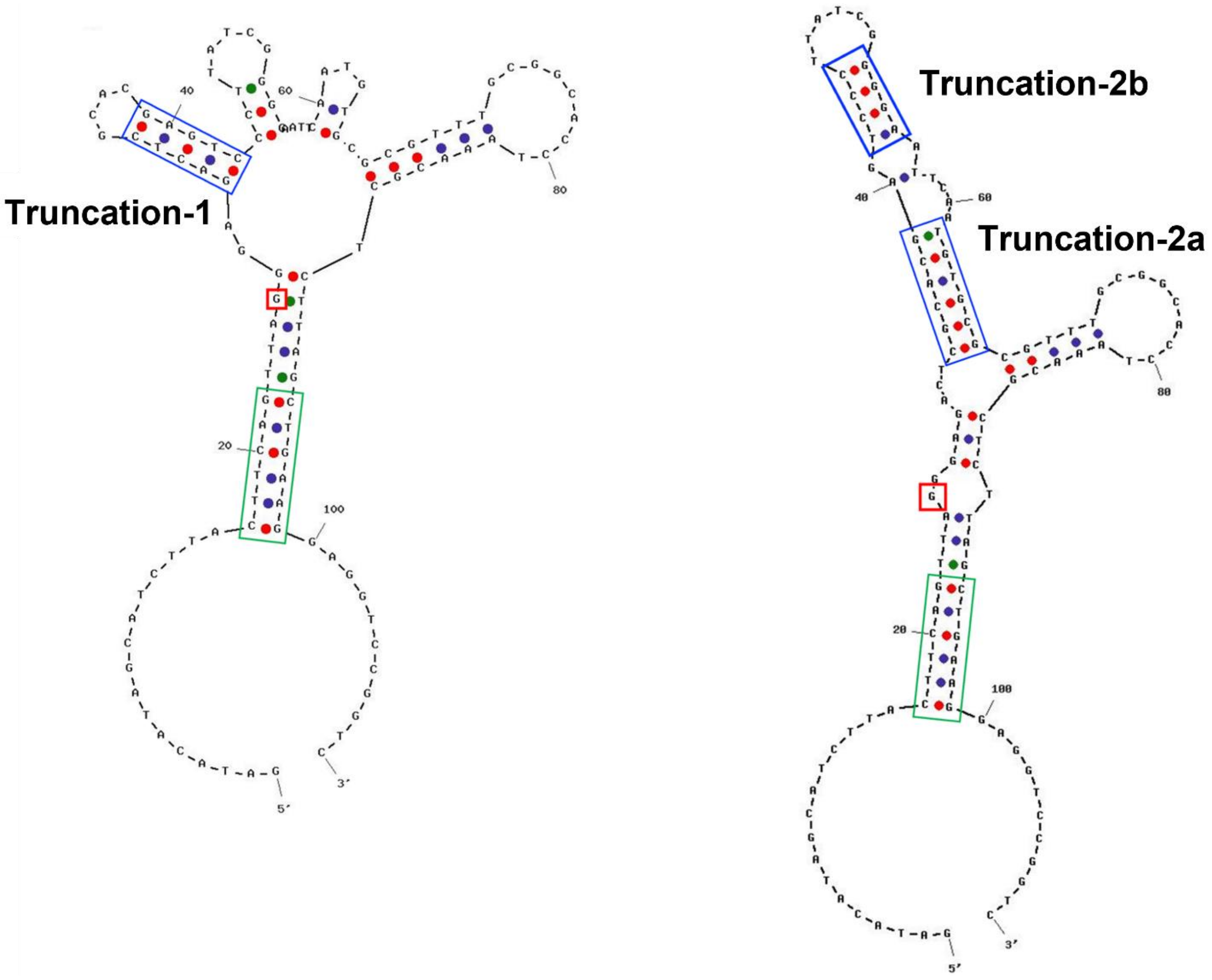

Fig. S21. Two predicted secondary structures of the cis-acting Fe(III)-B12 DNAzyme that represent different catalytic core conformations were selected for truncation studies. In each secondary structure, potential enzyme-substrate binding arms (boxed in green and blue) were identified. Regions outside the binding arms were removed by truncation. Removed regions include 1) 3'- and 5'-tails below binding arms boxed in green and 2) loop nucleotides on the left side of the binding arm boxed in blue. Binding arms in trans-acting DNAzymes were extended if their melting temperature was below 20 °C to support proper enzyme-substrate hybridization at room temperature. The intended cleavage site is a single guanosine ribonucleotide (rG), which is boxed in red. Activity of trans-acting DNAzymes were tested in the presence of Fe^{3+} to identify the minimal catalytic sequence that efficiently cleaves the substrate strand. Both versions of truncation-2 were equally active. Therefore, truncation-2a, which is the minimal catalytic region, was used to design the Fe(III)-B12 fluorescent sensor (Figure 1B).

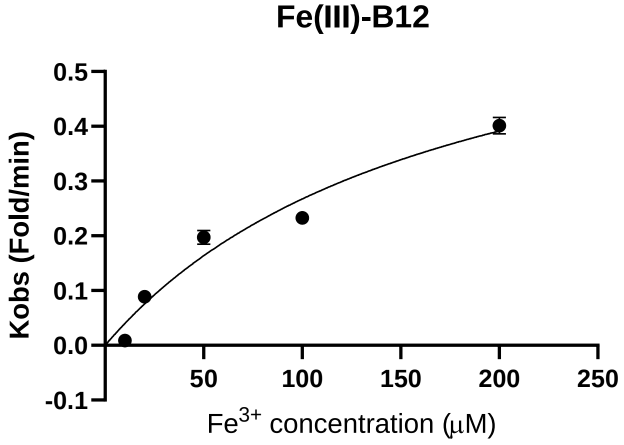

Fig. S22. K_{obs} of the Fe(III)-B12 sensor based on one-phase decay fitting with the fluorescence increase towards different concentrations of Fe^{3+}.

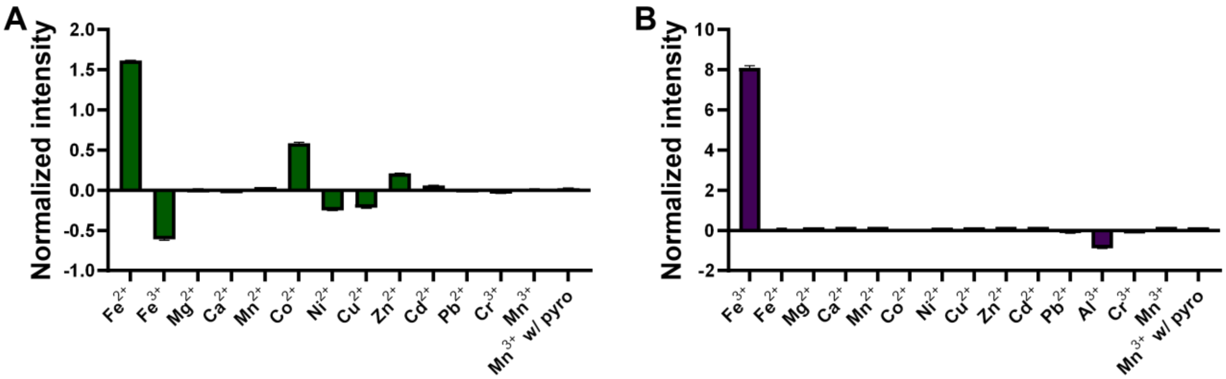

Fig. S23. Selectivity of Fe(II)-H5 and Fe(III)-B12 catalytic beacon sensors for alternative metal ions. Selectivity of the fluorescent catalytic beacon sensors of Fe(II)-H5 (A) and Fe(III)-B12 (B) against 200 µM of the relevant metal ion after 30 min incubation. Mn^{3+} was tested both with and without 5 mM pyruvate (w/ pyro) to help stabilize it in its oxidized form. Measurements were performed in 20 mM sodium acetate, 5 mM Bis-Tris, 200 mM NaCl at pH 6.0. The fluorescence intensity in the presence of different metal ions was normalized to the fluorescent signal without addition of divalent or trivalent metal ions (i.e., just buffer) at 30-minute time point, for a direct comparison. As a result, fluorescence quenching by the paramagnetic Fe^{3+}, Cu^{2+} and Ni^{2+} resulted in a negative response. The concentration (200 µM) used in this study is higher than that of Fe^{3+} under most physiological conditions.

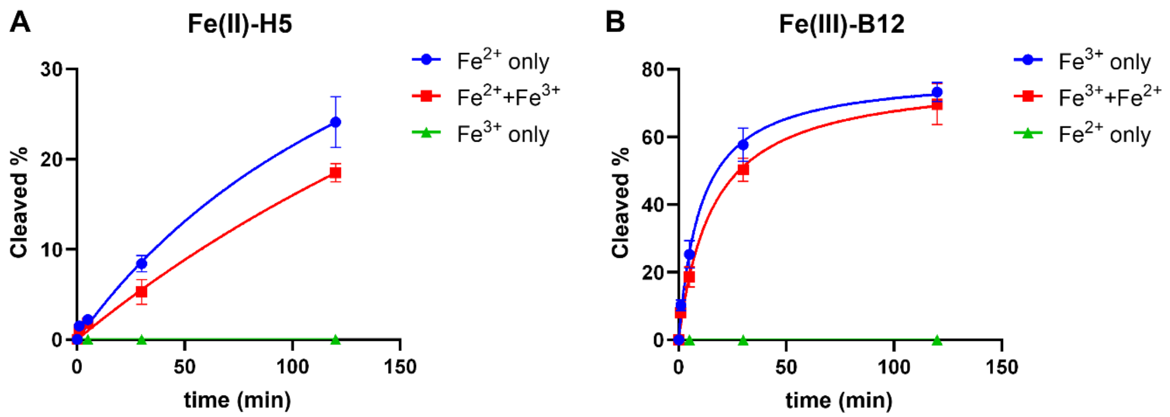

Fig. S24. Cleavage activity in the presence of Fe^{2+}, Fe^{3+}, or a mixture of both for the Fe(II)-H5 and Fe(III)-B12 DNAzymes. (A) DNAzyme activity for the Fe(II)-H5 DNAzyme was monitored with 100 µM Fe^{2+} in 20 mM acetate buffer pH 6.0, 5 mM Bis-Tris and 200 mM NaCl in the presence or absence of 20 µM Fe^{3+}. No activity was observed with only 20 µM Fe^{3+} (green triangles). (B) DNAzyme activity for the Fe(III)-B12 DNAzyme was monitored with 20 µM Fe^{3+} in 20 mM acetate buffer pH 6.0, 5 mM Bis-Tris and 200 mM NaCl in the presence or absence of 100 µM Fe^{2+}. No cleavage was noticed with 100 µM Fe^{2+} (blue circles).

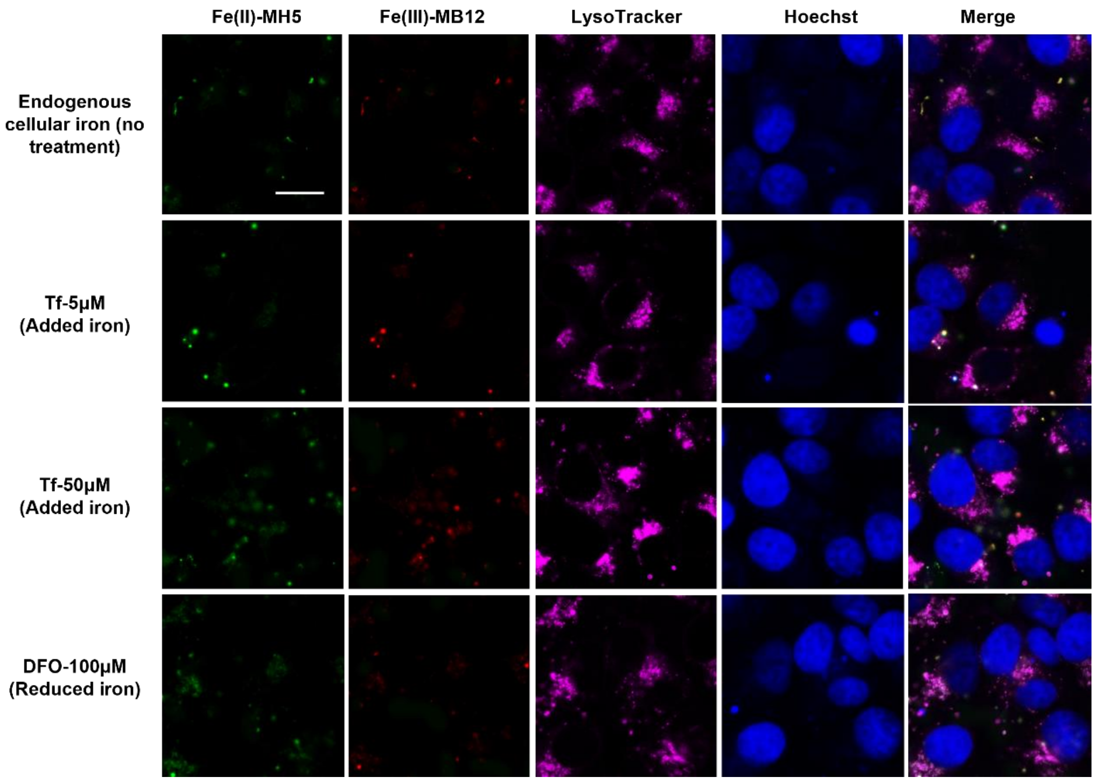

Fig. S25. Mutated DNAzyme sensor (iErS, Fe(II)-MH5, and Fe(III)-MB12), which cannot generate a signal due to the loss of activity, showed a low background signal in the endosomal-lysosomal system. Scale bar: 20 μm.

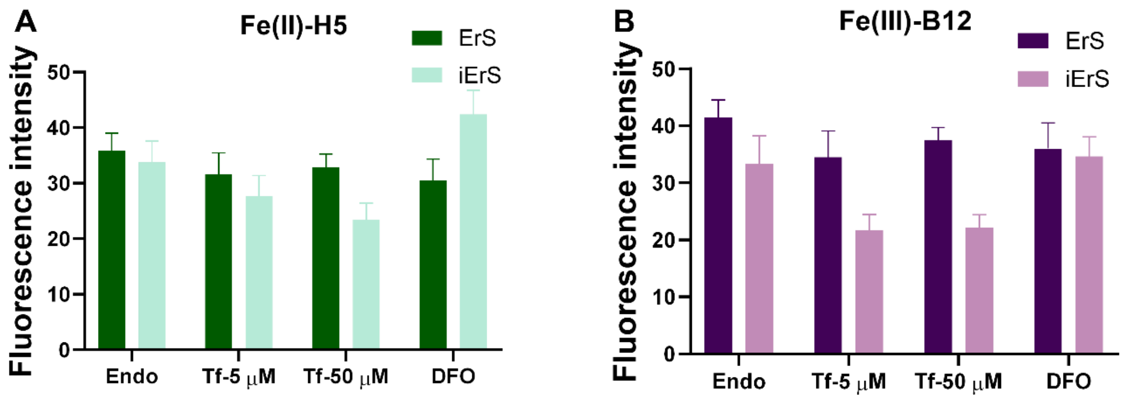

Fig. S26. Fluorescence signals from Fe^{2+} (A) and Fe^{3+} (B) sensors increased under transferrin (Tf) treatment while decreased under DFO treatment, indicating they are corelated to the changes in cellular iron concentration. Statistical analysis of mean fluorescence intensity in the LysoTracker labeled regions in Figure 2. One LysoTracker-labeled region per cell, five regions per picture, and four pictures were quantified for each group.

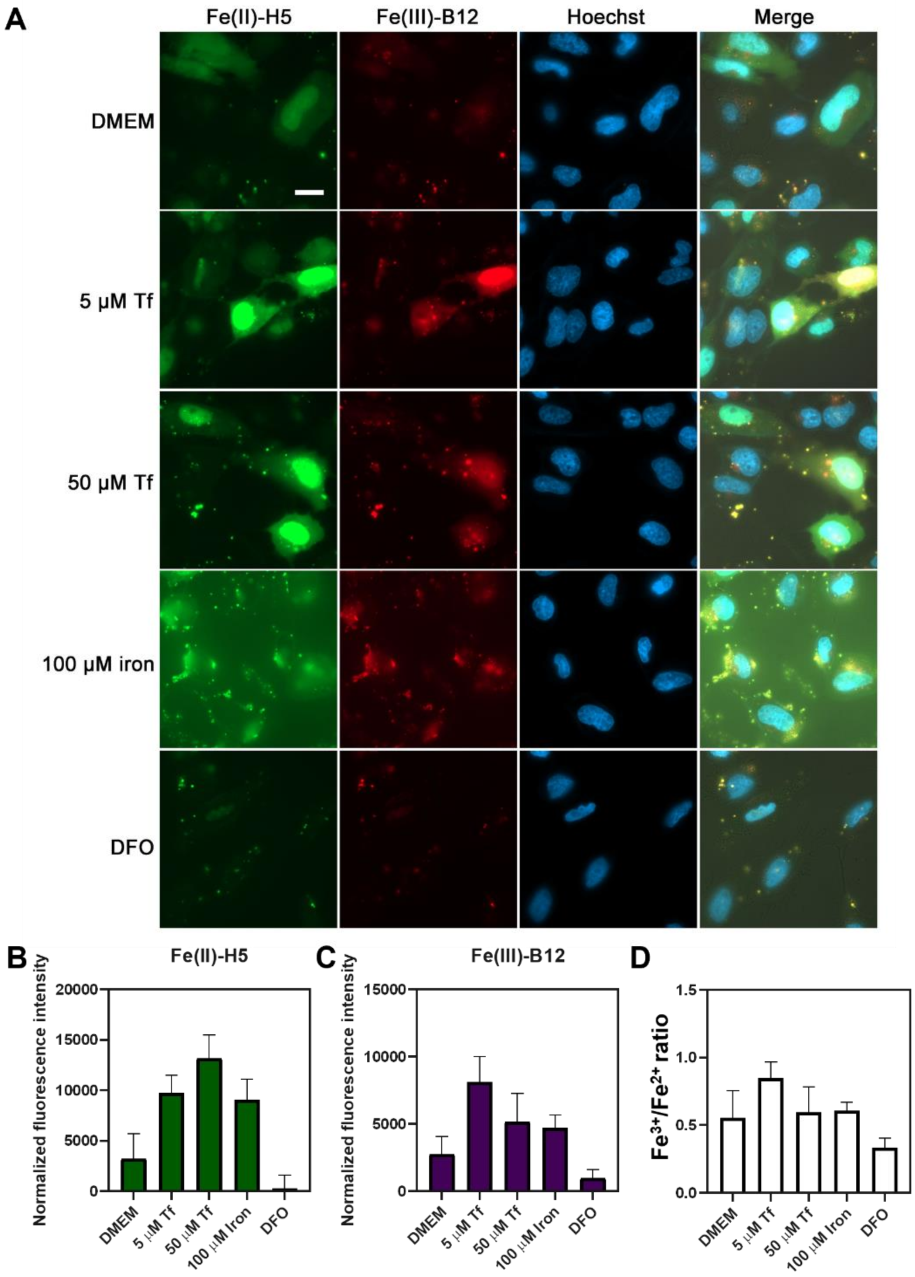

Fig. S27. Turbofect-delivered Fe(II)-H5 and Fe(III)-B12 DNAzyme sensors in Hela cells. (A) Without lysosomal targeted delivery, DNAzyme sensors (ErS) showed iron distribution in other subcellular locations such as nucleus and cytoplasm. Ferric ammonium citrate or transferrin (Tf) treatments increased fluorescence signaling, while DFO treatment decreased the fluorescence intensity. Cells were treated with normal cell media. Image was taken with Zeiss Axio Observer, 60x oil objective. Scale bar: 20 µm. (B-C) Statistical analysis for fluorescence intensity. The fluorescence intensity was quantified with image J, the mean fluorescence intensity per single cell was used, and 5 cells/picture and three pictures in total. The normalization was performed with the ErS-iErS.

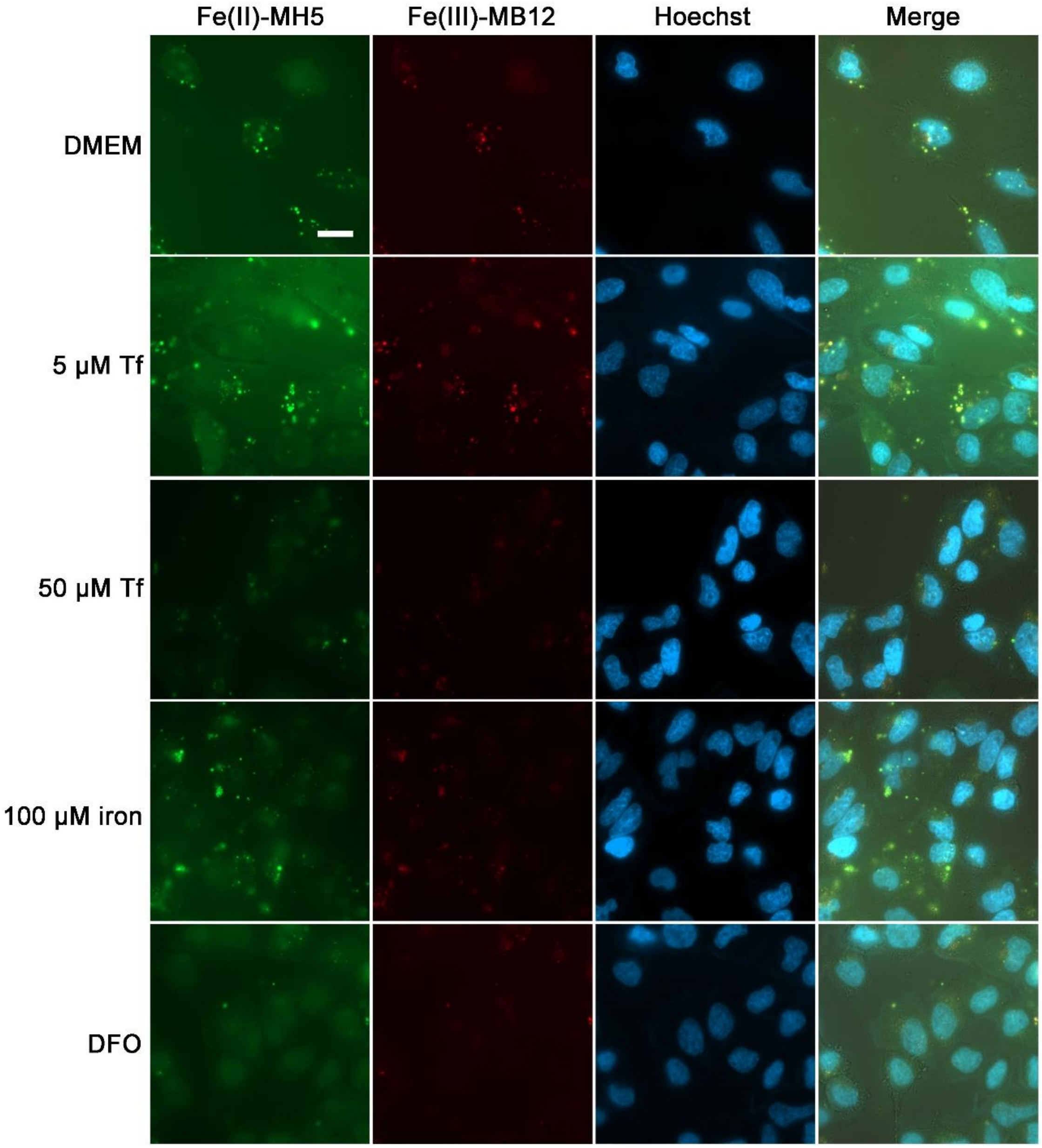

Fig. S28. Turbofect-delivered mutated DNAzyme sensor controls Fe(II)-MH5 and Fe(III)-MB12 in Hela cells. Low fluorescence background was observed with mutated DNAzyme sensor controls (iErS). In parallel experiments with the same experimental condition was used for Figs. S27 and S28. Image was taken with Zeiss Axio Observer, 60x oil objective. Scale bar: 20 μm.

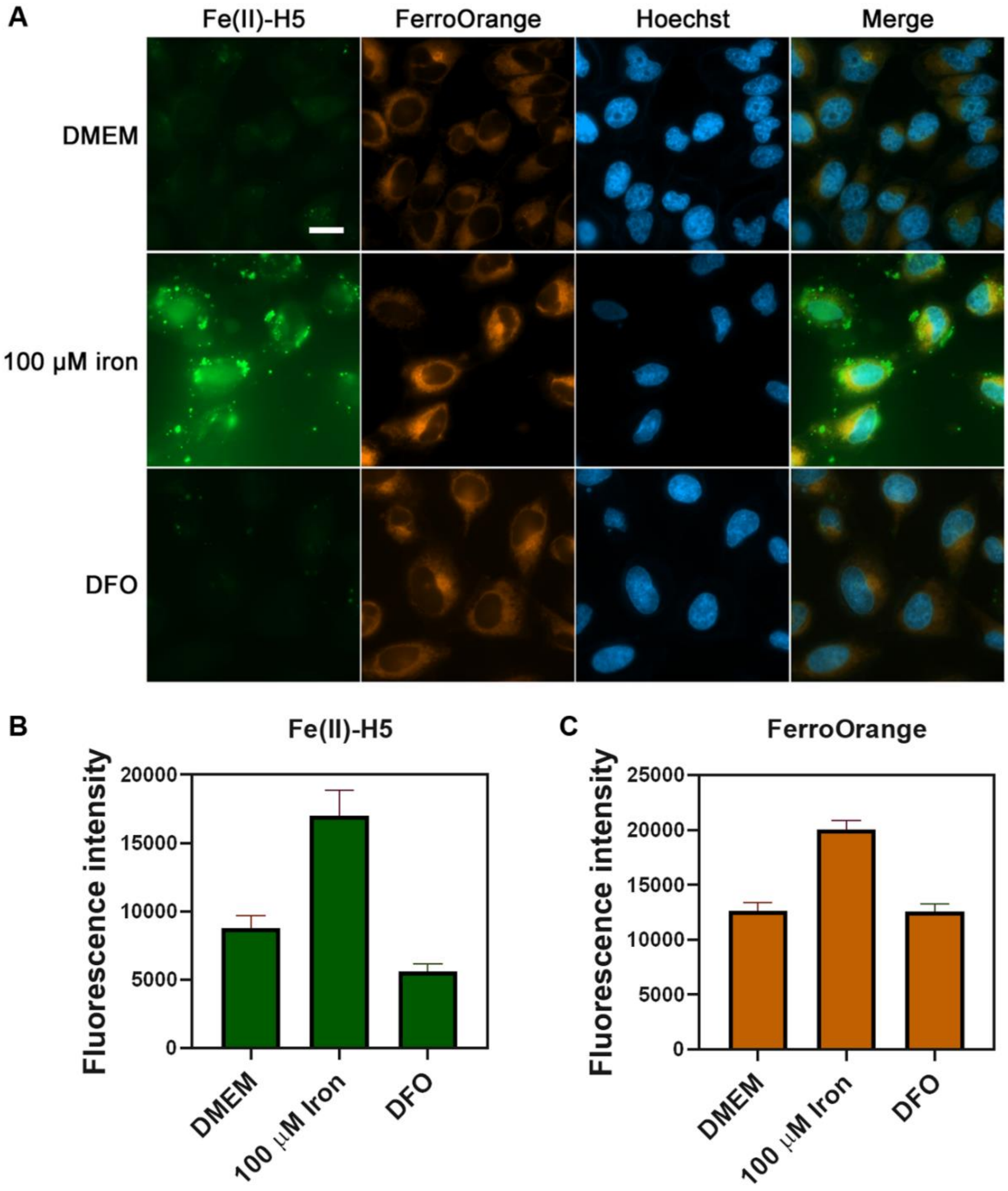

Fig. S29. A comparison between Fe(II)-H5 DNAzyme sensor and the commercial Fe^{2+} sensor FerroOrange. (A) Increase in fluorescence signals was observed for both sensors when adding iron (ferric ammonium citrate). Decrease in fluorescence signals was observed with Fe(II)-H5 DNAzyme sensor when treating the cells with the iron chelator DFO , but no significant difference was observed with FerroOrange when compared to DMEM group, which represents the intrinsic iron level. Images were taken with Zeiss Axio Observer, 60x oil objective. Scale bar: 20 μm. (B-C) Quantification for the fluorescence of Fe(II)-H5 DNAzyme (B) and FerroOrange (C) under different treatments. The fluorescence intensity represents the mean fluorescence intensity in a single cell. 5 cells per image and 4 images per group were quantified with ImageJ.

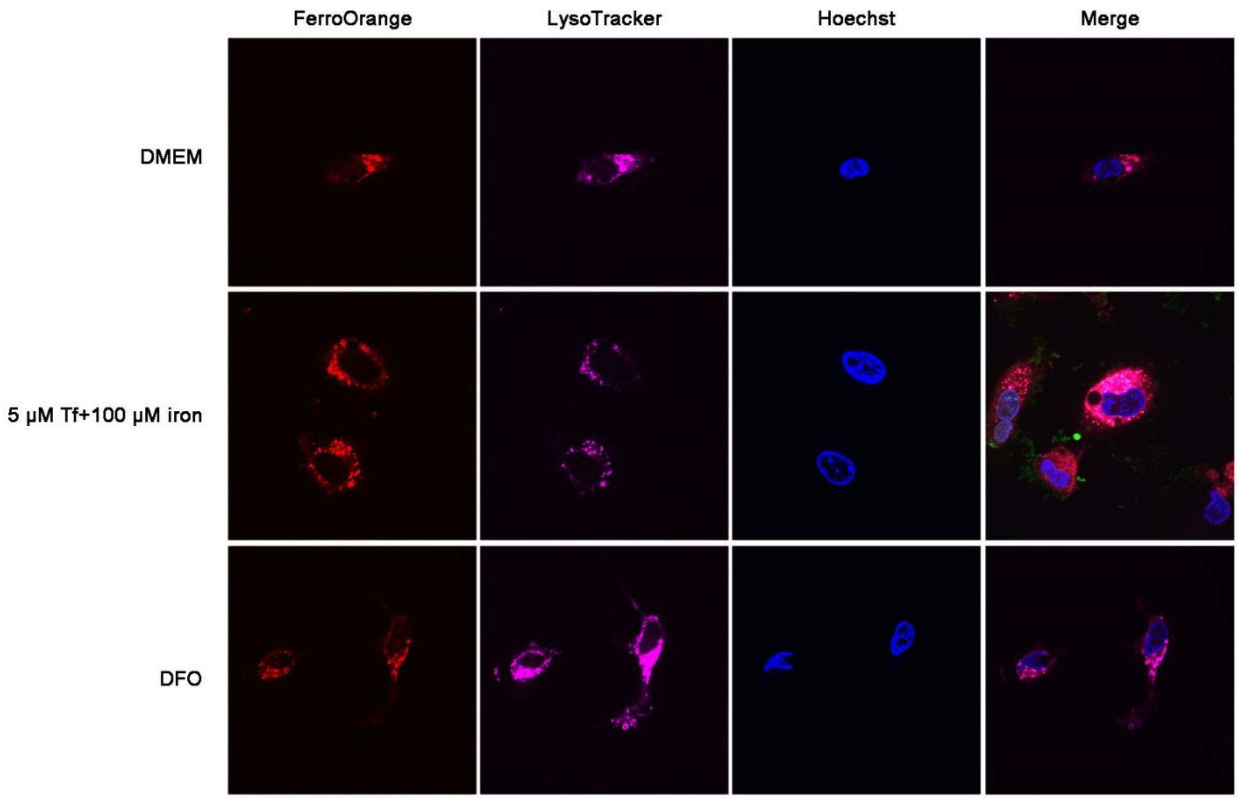

Fig. S30. Delivered commercial Fe^{2+} sensor FerroOrange with PEI. The sensor showed colocalization with LysoTracker, which is a similar pattern with the Fe(II)-H5 DNAzyme sensor. Images were taken with Nikon spinning disk confocal, 100x oil objective.

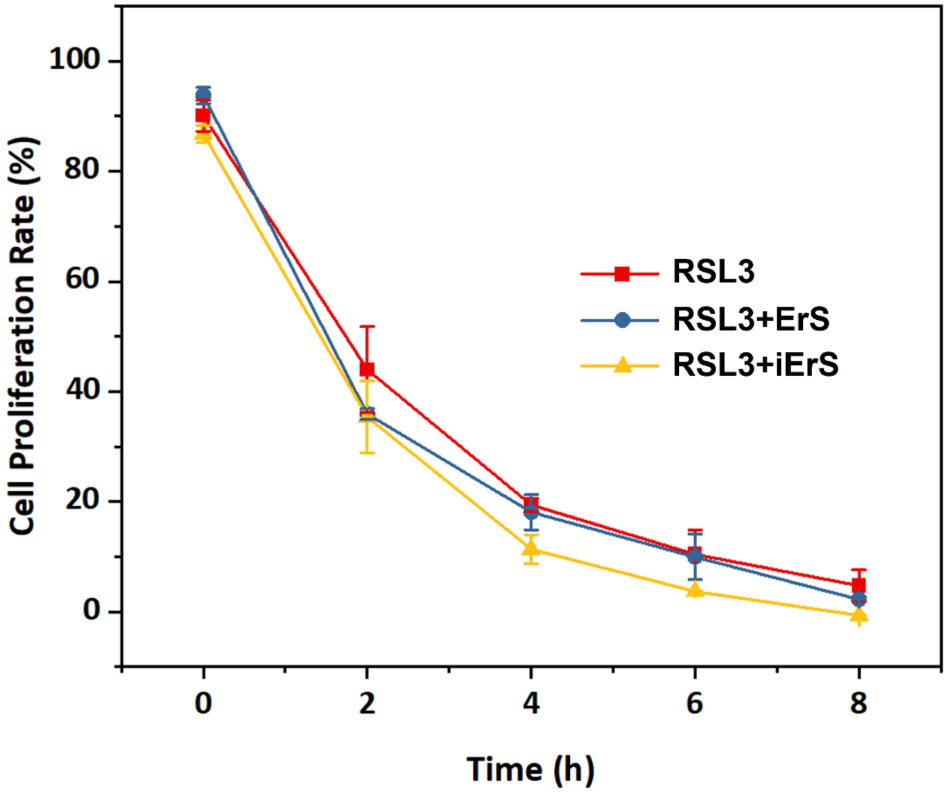

Fig. S31. MTT cytotoxicity assay of HepG2 cells treated with RSL3 from 0 hours to 8 hours. Measured when cells were treated without DNA (RSL3), with the active DNAzyme construct (RSL3 + ErS), or with the inactive DNAzyme construct (RSL3 + iErS).

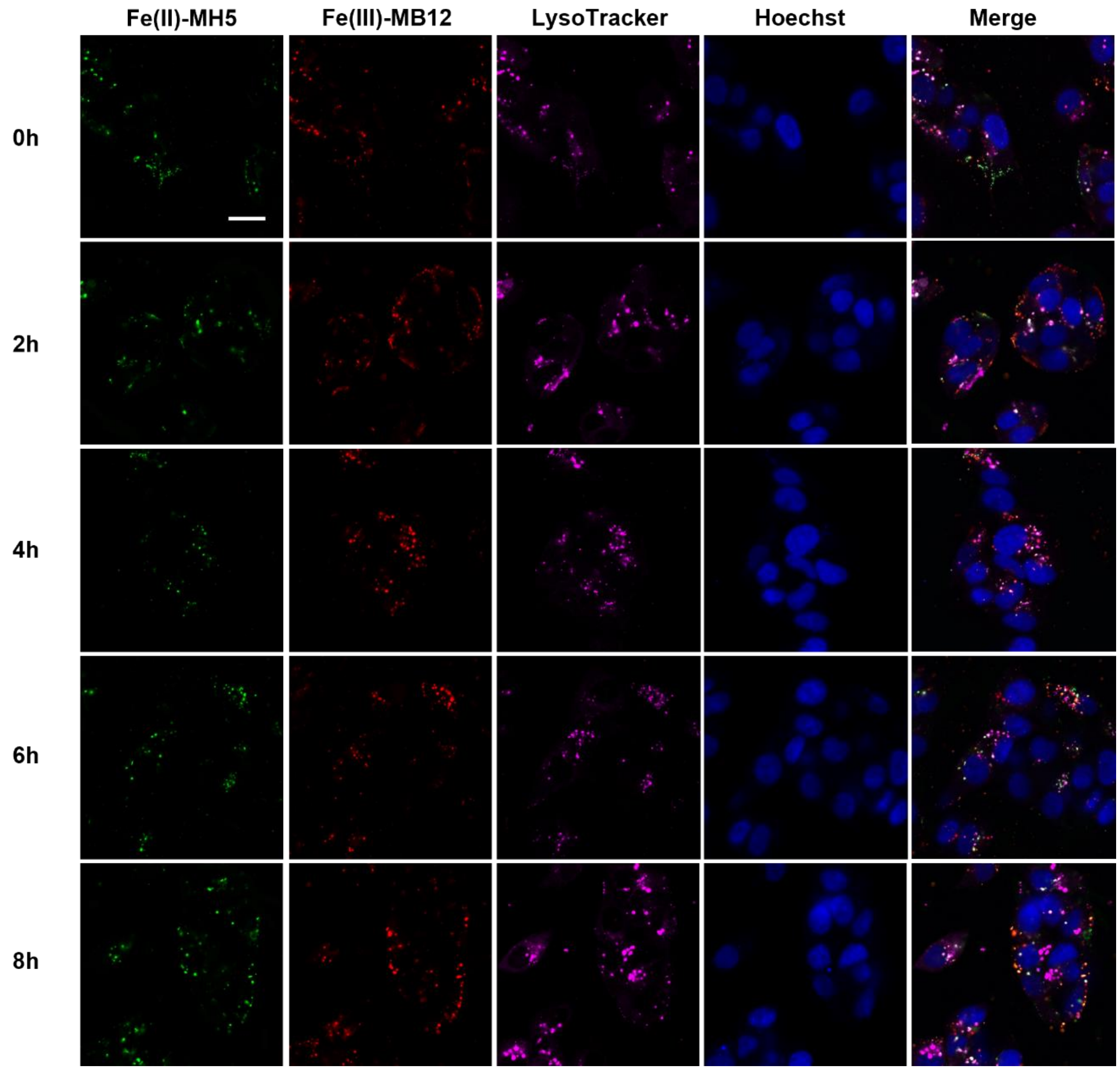

Fig. S32. Mutated DNAzyme sensor (iErS, Fe(II)-MH5, and Fe(III)-MB12), which cannot generate a signal due to the loss of activity, showed the low background signaling in the endosomal-lysosomal system during RSL3-induced ferroptosis. Same imaging and exposure conditions were used for both DNAzyme sensors and the mutated sensors. Scale bar: 20 μm.

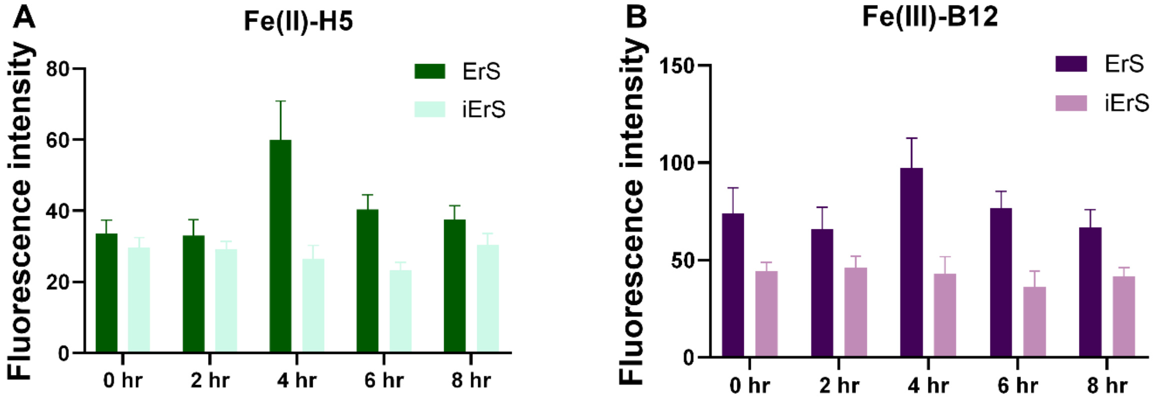

Fig. S33. Statistical analysis of fluorescence intensity of LysoTracker-labeled regions in Fig. 3. An increase of Fe^{2+} (A) and Fe^{3+} (B) from 0-4 hrs and a decrease for both Fe^{2+} (A) and Fe^{3+} (B) from 4-8 hrs were observed. One LysoTracker enriched region per cell, five regions per picture, and four pictures were quantified for each group.

Table S1.

Assigned letters for different Fe^{2+} selected pools. "R" indicates incorporation of counter selection before each positive selection, starting from round 3, and "+GSH" indicates incorporation of 1mM reduced glutathione.

Selection Condition	Number of Sequenced Clones	Letter Code
Fe^{2+}	34	E
Fe^{2+}+GSH	40	F
Fe^{2+}-R	36	G
Fe^{2+}+GSH-R	39	H

Table S2.

Assigned letters to different Fe^{3+} selected pools. Random regions of 35 or 50 nucleotides are indicated, and "NN" denotes selections carried out without negative selection.

Selection Condition	Number of Sequenced Clones	Letter Code
N35	39	A
N50	40	B
N35 (NN)	39	C
N50 (NN)	39	D

Table S3.

Pearson's correlation coefficient analysis for the colocalization between Fe^{2+} and Fe^{3+} signals, and LysoTracker, respectively. ErS: active DNAzyme sensors, iErS: point mutated inactive DNAzyme sensors.

ErS	Fe(II)-H5	Fe(III)-B12
Endo	61.8%	58.8%
Tf 5uM	65.4%	58.7%
Tf 50uM	76.0%	69.5%
DFO	54.7%	43.7%

iErS	Fe(II)-H5	Fe(III)-B12
Endo	54.8%	56.7%
Tf 5uM	50.1%	55.7%
Tf 50uM	55.5%	54.4%
DFO	75.1%	60.7%

Table S4.

List of DNA sequences.

Name	Sequence (5’-3’)
**Fe^{2+} In Vitro Selection**	
Fe^{2+} IDT Template	GGAAGGAATCGTACGATTCC-**N50**-CGTGATGCCTCTACCTC
Fe^{2+} Full Length Pool	GACTGGTATCAATCTCACGTATrAGGAAGGAATCGTACGATTCC-**N50**-CGTGATGCCTCTACCTC
Fe^{2+} P1	GTA TCA ATC TCA CGT ATA GGA AGG AAT CGT AC
Fe^{2+} P2	CGT GAT GCC TCT ACC TC
Fe^{2+} P2-iSp	(AAC)5 **-Sp-**C**18-** CGT GAT GCC TCT ACC TC
Fe^{2+} P3	GAC TGG TAT CAA TCT CAC GTA TrA
**Fe^{3+} In Vitro Selection**	
Fe^{3+} IDT Template	CCGGACCTCCTTCAG-**N35/50**-GACTCGTGCGAGTCTCCCTAACTGAAGTAAG-SpC3
Fe^{3+} Full Length Pool	GATACATAGCATCTTACTTCAGTTArGGGAGACTCGCACGAGTC- **N35/50**-CTGAAGGAGGTCCGGTC
Fe^{3+} P1	GCATCTTACTTCAGTTAGGGAGACTCGCACG
Fe^{3+} P2	GACCGGACCTCCTTCAG
Fe^{3+} P2-iSp	GAC(AAC)4- **Sp-C18**- GACCGGACCTCCTTCAG
Fe^{3+} P3	GATACATAGCATCTTACTTCAGTTArG
**Full-Length “cis” DNAzymes**	
Fe(II)-H5	GACTGGTATCAATCTCACGTATAGGAAGGAATCGTACGTTCCGTTATGGTTCTTTCTCCTAGCCAGACTGTTATGTGTGATACGGCAAACTTCGTGATGCCTCTACCTC
Fe(III)-B12	GATACATAGCATCTTACTTCAGTTAGGGAGACTCGCACGAGTCCCTTATCGGGGAATTCAATGTGCGCGTTTGCGGCACCTAAACGCTCTTAGCTGAAGGAGGTCCGGTC
**Catalytic Beacon DNAzyme Sensors**	
Fe(III)-B12-E	GCG GCA TGC GCG TTT GCG GCA CCT AAA CGC TCC TAA TAG AG/3IAbRQSp/
Fe(III)-B12-iE	GCG GCA TGC GCG TTT GCG GCA CCT AAA CGC CCC TAA TAG AG/3IAbRQSp/
Fe(III)-B12-rS	/5Alex647N/ CTC TAT TA rG GGA GAC TCG CAT GCC GC /3IAbRQSp/
Fe(II)-H5-E	/5IABkFQ/ TGG ATA TCT CCT AGC CAG ACT GTT ATG TGT GAT ACG GCA AAC TTC GTG ATG CCT CTA CGG GTC CG
Fe(II)-H5-iE	/5IABkFQ/ TGG ATA TCT CCT GGC CAG ACT GTT ATG TGT GAT ACG GCA AAC TTC GTG ATG CCT CTA CGG GTC CG
Fe(II)-H5-rS	/5IABkFQ/ CGG ACC CGT ATC AAT CTC ACG TAT rAGG ATA TCC A /3AlexF488N/

Table S5.

Fe^{2+}-dependent DNAzyme in vitro selection condition for selections E and F.

Selection round	Incubation time (min)	$[Fe^{2+}]$ (μM)
1	60	500
2	60	500
3	40	500
4	30	250
5	10	100
6	5	50
7	5	10
8	5	10
9	5	5

Table S6.

Fe^{2+}-dependent DNAzyme *in vitro* selection condition for selections G and H. From round 3 before each positive selection one counter selection step was introduced. In counter selection steps, DNA sequences that were active in a mixture of 1 mM Mn^{2+}, Co^{2+}, Zn^{2+}, Cd^{2+}, and Pb^{2+} were removed. No counter selection was introduced before the first two rounds of the selection.

Selection round	Incubation time (min)	$[Fe^{2+}]$ (μM)
1	60	500
2	60	500
3-R	40	500
4-R	30	250
5-R	30	250
6-R	10	100
7-R	5	50
8-R	5	50
9-R	5	25

Table S7.

Fe^{3+}-dependent DNAzyme *in vitro* selection conditions. In one condition a negative selection step was carried out for 24 h before positive selections. In the other condition, after round three, the negative selection discontinued (round 4NN to 9NN).

Selection Round	Incubation time (min)	$[Fe^{3+}]$ (μM)
	Negative selection	
1-4	60	50
5	15	50
6	3	50
7-9	3	5
	No negative Selection	
4-5-NN	60	50
6-NN	10	50
7-NN	3	50
8-9-NN	3	5

REFERENCES AND NOTES

1. D. Galaris, A. Barbouti, K. Pantopoulos, Iron homeostasis and oxidative stress: An intimate relationship. *Biochim. Biophys. Acta Mol. Cell Res.* **1866**, 118535 (2019).

2. N. Kim, H. J. Lee, Redox-active metal ions and amyloid-degrading enzymes in Alzheimer's disease. *Int. J. Mol. Sci.* **22**, 7697 (2021).

3. E. L. Que, D. W. Domaille, C. J. Chang, Metals in neurobiology: Probing their chemistry and biology with molecular imaging. *Chem. Rev.* **108**, 1517–1549 (2008).

4. K. P. Carter, A. M. Young, A. E. Palmer, Fluorescent sensors for measuring metal ions in living systems. *Chem. Rev.* **114**, 4564–4601 (2014).

5. J. M. Braughler, L. A. Duncan, R. L. Chase, The involvement of iron in lipid peroxidation. Importance of ferric to ferrous ratios in initiation. *J. Biol. Chem.* **261**, 10282–10289 (1986).

6. S. J. Dixon, K. M. Lemberg, M. R. Lamprecht, R. Skouta, E. M. Zaitsev, C. E. Gleason, D. N. Patel, A. J. Bauer, A. M. Cantley, W. S. Yang, B. Morrison, B. R. Stockwell, Ferroptosis: An iron-dependent form of nonapoptotic cell death. *Cell* **149**, 1060–1072 (2012).

7. B. R. Stockwell, J. P. Friedmann Angeli, H. Bayir, A. I. Bush, M. Conrad, S. J. Dixon, S. Fulda, S. Gascón, S. K. Hatzios, V. E. Kagan, K. Noel, X. Jiang, A. Linkermann, M. E. Murphy, M. Overholtzer, A. Oyagi, G. C. Pagnussat, J. Park, Q. Ran, C. S. Rosenfeld, K. Salnikow, D. Tang, F. M. Torti, S. V. Torti, S. Toyokuni, K. A. Woerpel, D. D. Zhang, Ferroptosis: A regulated cell death nexus linking metabolism, redox biology, and disease. *Cell* **171**, 273–285 (2017).

8. A. Ashraf, J. Jeandriens, H. G. Parkes, P.-W. So, Iron dyshomeostasis, lipid peroxidation and perturbed expression of cystine/glutamate antiporter in Alzheimer's disease: Evidence of ferroptosis. *Redox Biol.* **32**, 101494 (2020).

9. N. Yan, J. Zhang, Iron metabolism, ferroptosis, and the links with Alzheimer's disease. *Front. Neurosci.* **13**, 1443 (2020).

10. Z. Shen, J. Song, B. C. Yung, Z. Zhou, A. Wu, X. Chen, Emerging strategies of cancer therapy based on ferroptosis. *Adv. Mater.* **30**, e1704007 (2018).

11. T. Xu, W. Ding, X. Ji, X. Ao, Y. Liu, W. Yu, J. Wang, Molecular mechanisms of ferroptosis and its role in cancer therapy. *J. Cell. Mol. Med.* **23**, 4900–4912 (2019).

12. A. Spolaor, P. Vallelonga, J. Gabrieli, G. Cozzi, C. Boutron, C. Barbante, Determination of Fe^{2+} and Fe^{3+} species by FIA-CRC-ICP-MS in Antarctic ice samples. *J. Anal. At. Spectrom.* **27**, 310–317 (2012).

13. Q. Hu, Simultaneous separation and quantification of iron and transition species using LC-ICP-MS. *Am. J. Anal. Chem.* **2**, 675–682 (2011).

14. N. Mironova-Ulmane, M. Polakov, A. Pavlenko, T. Zvagule, M. Eglite, E. Churbakova, N. Kurjane, T. Kärner, "The Optical And Epr Spectra of Fe2+ and Fe3+ Ions in the Blood of the

Chernobyl Clean-Up Worker" in *World Congress on Medical Physics and Biomedical Engineering 2006*, R. Magjarevic, J. H. Nagel, Eds. (Springer, 2007), *IFMBE Proceedings*, pp. 2096–2098.

15. S. Sasaki, Fe^{2+} and Fe^{3+} ions distinguishable by x-ray anomalous scattering: Method and its application to magnetite. *Rev. Sci. Instrum.* **66**, 1573 (1995).

16. R. J. Ogg, J. W. Langston, E. M. Haacke, R. G. Steen, J. S. Taylor, The correlation between phase shifts in gradient-echo MR images and regional brain iron concentration. *Magn. Reson. Imaging* **17**, 1141–1148 (1999).

17. O. Dietrich, J. Levin, S.-A. Ahmadi, A. Plate, M. F. Reiser, K. Bötzel, A. Giese, B. Ertl-Wagner, MR imaging differentiation of Fe^{2+} and Fe^{3+} based on relaxation and magnetic susceptibility properties. *Neuroradiology* **59**, 403–409 (2017).

18. L. H. P. Vroegindeweij, L. Bossoni, A. J. W. Boon, J. H. P. Wilson, M. Bulk, J. Labra-Muñoz, M. Huber, A. Webb, L. van der Weerd, J. G. Langendonk, Quantification of different iron forms in the aceruloplasminemia brain to explore iron-related neurodegeneration. *NeuroImage Clin.* **30**, 102657 (2021).

19. W. Breuer, S. Epsztejn, Z. I. Cabantchik, Iron acquired from transferrin by K562 cells is delivered into a cytoplasmic pool of chelatable iron(II). *J. Biol. Chem.* **270**, 24209–24215 (1995).

20. O. Kakhlon, Z. I. Cabantchik, The labile iron pool: Characterization, measurement, and participation in cellular processes1 1This article is part of a series of reviews on “Iron and Cellular Redox Status.” The full list of papers may be found on the homepage of the journal. *Free Radic. Biol. Med.* **33**, 1037–1046 (2002).

21. M. Kruszewski, Labile iron pool: The main determinant of cellular response to oxidative stress. *Mutat. Res.* **531**, 81–92 (2003).

22. M. A. Smith, P. L. R. Harris, L. M. Sayre, G. Perry, Iron accumulation in Alzheimer disease is a source of redox-generated free radicals. *Proc. Natl. Acad. Sci. U.S.A.* **94**, 9866–9868 (1997).

23. H. Y. Au-Yeung, J. Chan, T. Chantarojsiri, C. J. Chang, Molecular imaging of labile iron(II) pools in living cells with a turn-on fluorescent probe. *J. Am. Chem. Soc.* **135**, 15165–15173 (2013).

24. A. T. Aron, M. O. Loehr, J. Bogena, C. J. Chang, An endoperoxide reactivity-based FRET probe for ratiometric fluorescence imaging of labile iron pools in living cells. *J. Am. Chem. Soc.* **138**, 14338–14346 (2016).

25. T. Hirayama, H. Tsuboi, M. Niwa, A. Miki, S. Kadota, Y. Ikeshita, K. Okuda, H. Nagasawa, A universal fluorogenic switch for Fe(II) ion based on N-oxide chemistry permits the visualization of intracellular redox equilibrium shift towards labile iron in hypoxic tumor cells. *Chem. Sci.* **8**, 4858–4866 (2017).

26. T. Hirayama, M. Niwa, S. Hirosawa, H. Nagasawa, High-throughput screening for the discovery of iron homeostasis modulators using an extremely sensitive fluorescent probe. *ACS Sens.* **5**, 2950–2958 (2020).

27. A. T. Aron, M. C. Heffern, Z. R. Lonergan, M. N. Vander Wal, B. R. Blank, B. Spangler, Y. Zhang, H. M. Park, A. Stahl, A. R. Renslo, E. P. Skaar, C. J. Chang, In vivo bioluminescence imaging of labile iron accumulation in a murine model of *Acinetobacter baumannii* infection. *Proc. Natl. Acad. Sci. U.S.A.* **114**, 12669–12674 (2017).

28. R. K. Muir, N. Zhao, J. Wei, Y.-H. Wang, A. Moroz, Y. Huang, Y.-C. Chen, R. Sriram, J. Kurhanewicz, D. Ruggero, A. R. Renslo, M. J. Evans, Measuring dynamic changes in the labile iron pool in vivo with a reactivity-based probe for positron emission tomography. *ACS Cent. Sci.* **5**, 727–736 (2019).

29. T. Hirayama, A. Miki, H. Nagasawa, Organelle-specific analysis of labile Fe(II) during ferroptosis by using a cocktail of various colour organelle-targeted fluorescent probes. *Metallomics* **11**, 111–117 (2019).

30. R. R. Breaker, G. F. Joyce, A DNA enzyme that cleaves RNA. *Chem.Biol.* **1**, 223–229 (1994).

31. Y. Li, R. R. Breaker, Deoxyribozymes: New players in the ancient game of biocatalysis. *Curr. Opin. Struct. Biol.* **9**, 315–323 (1999).

32. J. Li, W. Zheng, A. H. Kwon, Y. Lu, In vitro selection and characterization of a highly efficient Zn(II)-dependent RNA-cleaving deoxyribozyme. *Nucleic Acids Res*. **28**, 481–488 (2000).

33. Y. Lu, New transition metal-dependent DNAzymes as efficient endonucleases and as selective metal biosensors. *Chemistry* **8**, 4588–4596 (2002).

34. M. Cepeda-Plaza, E. L. Null, Y. Lu, Metal ion as both a cofactor and a probe of metal-binding sites in a uranyl-specific DNAzyme: A uranyl photocleavage study. *Nucleic Acids Res.* **41**, 9361–9370 (2013).

35. K. Hwang, P. Hosseinzadeh, Y. Lu, Biochemical and biophysical understanding of metal ion selectivity of DNAzymes. *Inorganica Chim. Acta*. **452**, 12–24 (2016).

36. R. J. Lake, Z. Yang, J. Zhang, Y. Lu, DNAzymes as activity-based sensors for metal ions: Recent applications, demonstrated advantages, current challenges, and future directions. *Acc. Chem. Res.* **52**, 3275–3286 (2019).

37. H. E. Ihms, Y. Lu, "In Vitro Selection of Metal Ion-Selective DNAzymes" in *Ribozymes. Methods in Molecular Biology*, J. S. Hartig, Ed. (Humana Press, 2012), vol. 848, pp. 297–316.

38. J. Li, Y. Lu, A highly sensitive and selective catalytic DNA biosensor for lead ions. *J. Am. Chem. Soc.* **122**, 10466–10467 (2000).

39. J. Liu, Y. Lu, Improving fluorescent DNAzyme biosensors by combining inter- and intramolecular quenchers. *Anal. Chem.* **75**, 6666–6672 (2003).

40. J. Liu, A. K. Brown, X. Meng, D. M. Cropek, J. D. Istok, D. B. Watson, Y. Lu, A catalytic beacon sensor for uranium with parts-per-trillion sensitivity and millionfold selectivity. *Proc. Natl. Acad. Sci. U.S.A.* **104**, 2056–2061 (2007).

41. J. Liu, Y. Lu, A DNAzyme catalytic beacon sensor for paramagnetic Cu^{2+} ions in aqueous solution with high sensitivity and selectivity. *J. Am. Chem. Soc.* **129**, 9838–9839 (2007).

42. J. Liu, Y. Lu, Rational design of "turn-on" allosteric DNAzyme catalytic beacons for aqueous mercury ions with ultrahigh sensitivity and selectivity. *Angew. Chem. Int. Ed. Engl.* **46**, 7587–7590 (2007).

43. T. Lan, K. Furuya, Y. Lu, A highly selective lead sensor based on a classic lead DNAzyme. *Chem. Commun. (Camb)* **46**, 3896–3898 (2010).

44. C. E. McGhee, K. Y. Loh, Y. Lu, DNAzyme sensors for detection of metal ions in the environment and imaging them in living cells. *Curr. Opin. Biotechnol.* **45**, 191–201 (2017).

45. Z. Yang, K. Y. Loh, Y.-T. Chu, R. Feng, N. S. R. Satyavolu, M. Xiong, S. M. Nakamata Huynh, K. Hwang, L. Li, H. Xing, X. Zhang, Y. R. Chemla, M. Gruebele, Y. Lu, Optical control of metal ion probes in cells and zebrafish using highly selective DNAzymes conjugated to upconversion nanoparticles. *J. Am. Chem. Soc.* **140**, 17656–17665 (2018).

46. Y. Lin, Z. Yang, R. J. Lake, C. Zheng, Y. Lu, Enzyme-mediated endogenous and bioorthogonal control of a DNAzyme fluorescent sensor for imaging metal ions in living cells. *Angew. Chem. Int. Ed. Engl.* **58**, 17061–17067 (2019).

47. E. M. McConnell, I. Cozma, Q. Mou, J. D. Brennan, Y. Lu, Y. Li, Biosensing with DNAzymes. *Chem. Soc. Rev.* **50**, 8954–8994 (2021).

48. S. Xing, Y. Lin, L. Cai, P. N. Basa, A. K. Shigemoto, C. Zheng, F. Zhang, S. C. Burdette, Y. Lu, Detection and quantification of tightly bound Zn^{2+} in blood serum using a photocaged chelator and a DNAzyme fluorescent sensor. *Anal. Chem.* **93**, 5856–5861 (2021).

49. W. Zhou, M. Vazin, T. Yu, J. Ding, J. Liu, In vitro selection of chromium-dependent DNAzymes for sensing chromium(III) and chromium(VI). *Chemistry* **22**, 9835–9840 (2016).

50. W. Zhou, R. Saran, P.-J. J. Huang, J. Ding, J. Liu, An exceptionally selective DNA cooperatively binding two Ca^{2+} ions. *Chembiochem.* **18**, 518–522 (2017).

51. P.-J. J. Huang, J. Liu, Rational evolution of Cd^{2+-}specific DNAzymes with phosphorothioate modified cleavage junction and Cd^{2+} sensing. *Nucleic Acids Res.* **43**, 6125–6133 (2015).

52. P. Bruesehoff, J. Li, A. J. Augustine 3rd, Y. Lu, Improving metal ion specificity during in vitro selection of catalytic DNA. *Comb. Chem. High Throughput Screen.* **5**, 327–335 (2002).

53. N. Carmi, L. A. Shultz, R. R. Breaker, In vitro selection of self-cleaving DNAs. *Chem. Biol.* **3**, 1039–1046 (1996).

54. S. W. Santoro, G. F. Joyce, A general purpose RNA-cleaving DNA enzyme. *Proc. Natl. Acad. Sci. U.S.A.* **94**, 4262–4266 (1997).

55. S. W. Santoro, G. F. Joyce, K. Sakthivel, S. Gramatikova, C. F. Barbas 3rd, RNA cleavage by a DNA enzyme with extended chemical functionality. *J. Am. Chem. Soc.* **122**, 2433–2439 (2000).

56. K. E. Nelson, P. J. Bruesehoff, Y. Lu, In vitro selection of high temperature Zn_2^+-dependent DNAzymes. *J. Mol. Evol.* **61**, 216–225 (2005).

57. R. Saran, J. Liu, A silver DNAzyme. *Anal. Chem.* **88**, 4014–4020 (2016).

58. C. E. McGhee, Z. Yang, W. Guo, Y. Wu, M. Lyu, C. J. DeLong, S. Hong, Y. Ma, M. G. McInnis, K. S. O'Shea, Y. Lu, DNAzyme-based lithium-selective imaging reveals higher lithium accumulation in bipolar disorder patient-derived neurons. *ACS Cent. Sci.* **7**, 1809–1820 (2021).

59. S.-F. Torabi, P. Wu, C. E. McGhee, L. Chen, K. Hwang, N. Zheng, J. Cheng, Y. Lu, In vitro selection of a sodium-specific DNAzyme and its application in intracellular sensing. *Proc. Natl. Acad. Sci. U.S.A.* **112**, 5903–5908 (2015).

60. S.-F. Torabi, Y. Lu, Identification of the same Na^+-specific DNAzyme motif from two in vitro selections under different conditions. *J. Mol. Evol.* **81**, 225–234 (2015).

61. D. J. R. Lane, A. M. Merlot, M. L.-H. Huang, D.-H. Bae, P. J. Jansson, S. Sahni, D. S. Kalinowski, D. R. Richardson, Cellular iron uptake, trafficking and metabolism: Key molecules and mechanisms and their roles in disease. *Biochim. Biophys. Acta* **1853**, 1130–1144 (2015).

62. D. Richardson, P. Ponka, E. Baker, The effect of the iron(III) chelator, desferrioxamine, on iron and transferrin uptake by the human malignant melanoma cell. *Cancer Res.* **54**, 685–689 (1994).

63. R. C. Hider, X. Kong, Iron speciation in the cytosol: An overview. *Dalton Trans.* **42**, 3220–3229 (2013).

64. R. S. Ohgami, D. R. Campagna, A. McDonald, M. D. Fleming, The Steap proteins are metalloreductases. *Blood* **108**, 1388–1394 (2006).

65. K. Pantopoulos, S. K. Porwal, A. Tartakoff, L. Devireddy, Mechanisms of mammalian iron homeostasis. *Biochemistry* **51**, 5705–5724 (2012).

66. Q.-Q. Zhao, J.-L. Chen, T.-F. Lv, C.-X. He, G.-P. Tang, W.-Q. Liang, Y. Tabata, J.-Q. Gao, N/P ratio significantly influences the transfection efficiency and cytotoxicity of a polyethylenimine/chitosan/DNA complex. *Biol. Pharm. Bull.* **32**, 706–710 (2009).

67. P. A. Longo, J. M. Kavran, M.-S. Kim, D. J. Leahy, Transient mammalian cell transfection with polyethylenimine (PEI). *Methods Enzymol.* **529**, 227–240 (2013).

68. R. V. Benjaminsen, M. A. Mattebjerg, J. R. Henriksen, S. M. Moghimi, T. L. Andresen, The possible "proton sponge " effect of polyethylenimine (PEI) does not include change in lysosomal pH. *Mol. Ther.* **21**, 149–157 (2013).

69. B. Chazotte, Labeling lysosomes in live cells with LysoTracker. *Cold Spring Harb. Protoc.* **2011**, pdb.prot5571 (2011).

70. FerroOrange Labile ferrous ion detecting probe | Goryo Chemical Inc. *FerroOrange Labile Ferr. Ion Detect. Probe Goryo Chem. Inc.*; https://goryochemical.com/en/research-reagent/4703/.

71. H. Feng, B. R. Stockwell, Unsolved mysteries: How does lipid peroxidation cause ferroptosis? *PLOS Biol.* **16**, e2006203 (2018).

72. W. S. Yang, B. R. Stockwell, Synthetic lethal screening identifies compounds activating iron-dependent, nonapoptotic cell death in oncogenic-RAS-harboring cancer cells. *Chem. Biol.* **15**, 234–245 (2008).

73. R. Shintoku, Y. Takigawa, K. Yamada, C. Kubota, Y. Yoshimoto, T. Takeuchi, I. Koshiishi, S. Torii, Lipoxygenase-mediated generation of lipid peroxides enhances ferroptosis induced by erastin and RSL3. *Cancer Sci.* **108**, 2187–2194 (2017).

74. X. Sui, R. Zhang, S. Liu, T. Duan, L. Zhai, M. Zhang, X. Han, Y. Xiang, X. Huang, H. Lin, T. Xie, RSL3 drives ferroptosis through GPX4 inactivation and ROS production in colorectal cancer. *Front. Pharmacol.* **9**, 1371 (2018).

75. T. Mosmann, Rapid colorimetric assay for cellular growth and survival: Application to proliferation and cytotoxicity assays. *J. Immunol. Methods* **65**, 55–63 (1983).

76. J.-L. Liu, Y.-G. Fan, Z.-S. Yang, Z.-Y. Wang, C. Guo, Iron and Alzheimer's disease: From pathogenesis to therapeutic implications. *Front. Neurosci.* **12**, 632 (2018).

77. H. Oakley, S. L. Cole, S. Logan, E. Maus, P. Shao, J. Craft, A. Guillozet-Bongaarts, M. Ohno, J. Disterhoft, L. Van Eldik, R. Berry, R. Vassar, Intraneuronal β-amyloid aggregates, neurodegeneration, and neuron loss in transgenic mice with five familial Alzheimer's disease mutations: potential factors in amyloid plaque formation. *J. Neurosci.* **26**, 10129–10140 (2006).

78. J. H. Roh, Y. Huang, A. W. Bero, T. Kasten, F. R. Stewart, R. J. Bateman, D. M. Holtzman, Sleep-wake cycle and diurnal fluctuation of amyloid-β as biomarkers of brain amyloid pathology. *Sci. Transl. Med.* **4**, 150ra122 (2012).

79. D. G. Smith, R. Cappai, K. J. Barnham, The redox chemistry of the Alzheimer's disease amyloid β peptide. *Biochim. Biophys. Acta* **1768**, 1976–1990 (2007).

80. J. Everett, E. Céspedes, L. R. Shelford, C. Exley, J. F. Collingwood, J. Dobson, G. van der Laan, C. A. Jenkins, E. Arenholz, N. D. Telling, Evidence of redox-active iron formation following aggregation of ferrihydrite and the Alzheimer's disease peptide β-amyloid. *Inorg. Chem.* **53**, 2803–2809 (2014).

81. J. Everett, E. Céspedes, L. R. Shelford, C. Exley, J. F. Collingwood, J. Dobson, G. van der Laan, C. A. Jenkins, E. Arenholz, N. D. Telling, Ferrous iron formation following the co-aggregation of ferric iron and the Alzheimer's disease peptide β-amyloid (1–42). *J. R. Soc. Interface* **11**, 20140165 (2014).

82. M. Gao, P. Monian, Q. Pan, W. Zhang, J. Xiang, X. Jiang, Ferroptosis is an autophagic cell death process. *Cell Res.* **26**, 1021–1032 (2016).

83. G. O. Latunde-Dada, Ferroptosis: Role of lipid peroxidation, iron and ferritinophagy. *Biochim. Biophys. Acta Gen. Subj.* **1861**, 1893–1900 (2017).

84. F. Kuang, J. Liu, D. Tang, R. Kang, Oxidative damage and antioxidant defense in ferroptosis. *Front. Cell Dev. Biol.* **8**, 586578 (2020).

85. J. M. G. van Bergen, X. Li, J. Hua, S. J. Schreiner, S. C. Steininger, F. C. Quevenco, M. Wyss, A. F. Gietl, V. Treyer, S. E. Leh, F. Buck, R. M. Nitsch, K. P. Pruessmann, P. C. M. van Zijl, C. Hock, P. G. Unschuld, Colocalization of cerebral iron with amyloid β in mild cognitive impairment. *Sci. Rep.* **6**, 35514 (2016).

86. N. D. Telling, J. Everett, J. F. Collingwood, J. Dobson, G. van der Laan, J. J. Gallagher, J. Wang, A. P. Hitchcock, Iron biochemistry is correlated with amyloid plaque morphology in an established mouse model of Alzheimer's disease. *Cell Chem. Biol.* **24**, 1205–1215.e3 (2017).

87. J. Everett, J. Brooks, F. Lermyte, P. B. O'Connor, P. J. Sadler, J. Dobson, J. F. Collingwood, N. D. Telling, Iron stored in ferritin is chemically reduced in the presence of aggregating Aβ(1-42). *Sci. Rep.* **10**, 10332 (2020).

88. C. A. Schneider, W. S. Rasband, K. W. Eliceiri, NIH Image to ImageJ: 25 years of image analysis. *Nat. Methods* **9**, 671–675 (2012).

89. S. Bolte, F. P. Cordelières, A guided tour into subcellular colocalization analysis in light microscopy. *J. Microsc.* **224**, 213–232 (2006).

90. N. R. Markham, M. Zuker, UNAFold: Software for nucleic acid folding and hybridization. *Methods Mol. Biol.* **453**, 3–31 (2008).

91. S. W. Taylor, D. B. Chase, M. H. Emptage, M. J. Nelson, J. H. Waite, Ferric ion complexes of a DOPA-containing adhesive protein from *Mytilus edulis*. *Inorg. Chem.* **35**, 7572–7577 (1996).

92. D. S. Hwang, H. Zeng, A. Masic, M. J. Harrington, J. N. Israelachvili, J. H. Waite, Protein- and metal-dependent interactions of a prominent protein in mussel adhesive plaques. *J. Biol. Chem.* **285**, 25850–25858 (2010).

93. H. Zeng, D. S. Hwang, J. N. Israelachvili, J. H. Waite, Strong reversible Fe^{3+}-mediated bridging between dopa-containing protein films in water. *Proc. Natl. Acad. Sci. U.S.A.* **107**, 12850–12853 (2010).
